# A Systematic Approach to Dissection of the Equine Brain–Evaluation of a Species-Adapted Protocol for Beginners and Experts

**DOI:** 10.3389/fnana.2020.614929

**Published:** 2020-12-18

**Authors:** Maya-Lena Bitschi, Zoltán Bagó, Marco Rosati, Sven Reese, Lutz S. Goehring, Kaspar Matiasek

**Affiliations:** ^1^Section of Clinical and Comparative Neuropathology, Centre for Clinical Veterinary Medicine, Ludwig Maximilians University, Munich, Germany; ^2^Austrian Agency for Health and Food Safety Ltd. (AGES), Institute for Veterinary Disease Control, Mödling, Austria; ^3^Department of Veterinary Sciences, Institute of Anatomy, Histology & Embryology, Ludwig Maximilians University, Munich, Germany; ^4^Division of Medicine and Reproduction, Centre for Clinical Veterinary Medicine, Equine Hospital, Ludwig Maximilians University, Munich, Germany

**Keywords:** neuroanatomy, neuropathology, guideline, central nervous system, equine, horse, necropsy, brain atlas

## Abstract

Introduction of new imaging modalities for the equine brain have refocused attention on the horse as a natural model for ethological, neuroanatomical, and neuroscientific investigations. As opposed to imaging studies, strategies for equine neurodissection still lack a structured approach, standardization and reproducibility. In contrast to other species, where adapted protocols for sampling have been published, no comparable guideline is currently available for equids. Hence, we developed a species-specific slice protocol for whole brain vs. hemispheric dissection and tested its applicability and practicability in the field, as well as its neuroanatomical accuracy and reproducibility. Dissection steps are concisely described and depicted by schematic illustrations, photographs and instructional videos. Care was taken to show the brain in relation to the raters' hands, cutting devices and bench surface. Guidance is based on a minimum of external anatomical landmarks followed by geometric instructions that led to procurement of 14 targeted slabs. The protocol was performed on 55 formalin-fixed brains by three groups of investigators with different neuroanatomical skills. Validation of brain dissection outcomes addressed the aptitude of slabs for neuroanatomical studies as opposed to simplified routine diagnostic purposes. Across all raters, as much as 95.2% of slabs were appropriate for neuroanatomical studies, and 100% of slabs qualified for a routine diagnostic setting. Neither autolysis nor subfixation significantly affected neuroanatomical accuracy score, while a significant negative effect was observed with brain extraction artifacts. Procedure times ranged from 14 to 66 min and reached a mean duration of 23.25 ± 7.93 min in the last of five trials in inexperienced raters vs. 16 ± 2.83 min in experts, while acceleration of the dissection did not negatively impact neuroanatomical accuracy. This protocol, derived analogously to the consensus report of the International Veterinary Epilepsy Task Force in dogs and cats, allows for systematic, quick and easy dissection of the equine brain, even for inexperienced investigators. Obtained slabs feature virtually all functional subcompartments at suitable planes for both diagnostic and neuroscientific investigations and complement the data obtained from imaging studies. The instructive protocol and brain dissection videos are available in Supplementary Material.

## Introduction

With domestication dating back to ~3.500 BC, the domestic horse (*Equus caballus*) has become a close companion to human beings through farm work, war, sports, and leisure. With its complex gyrified (Zilles et al., 2013; Cozzi et al., 2014) and voluminous brain, its distinct cognitive skills and predictive behavior in a controlled environment (Brubaker and Udell, 2016; Roberts et al., 2017), its accessibility for neurological examination and neurophysiological testing (Pickles, 2019; Rijckaert et al., 2019), its compliance to perform controlled exercise and its long lifespan, the horse has regained attention as a natural model for ethological, neuroanatomic, and neuroscientific studies (Cozzi et al., 2014; Roberts et al., 2017; Johnson et al., 2019).

Murine disease models, surely, are most prevalent (Ehret et al., 2017) due to their easy handling, rapid reproduction, and genetic and environmental standardizability in a laboratory setting. However, regenerative capacities of the central nervous system between rodents and larger mammalian species differ significantly, and rodents' relatively short lifespans barely allow for modeling of longevity-associated phenomena, such as in neurodegeneration (Morton and Howland, 2013). Moreover, the rodent brain and skull architecture barely reflects human neuroanatomy from a topofunctional point of view (Morton and Howland, 2013; Potschka et al., 2013). Small brain volumes render certain interventional and diagnostic maneuvers, such as collection of cerebrospinal fluid (CSF) (Lim et al., 2018) and electroencephalography (EEG) (Potschka et al., 2013), more difficult and increase procedure-related morbidities.

Beyond these considerations, horses share a susceptibility as accidental hosts for multiple anthropozoonotic pathogens that affect the nervous system, such as Hendra and Nipah virus (HeV, NiV), West Nile virus (WNV), Japanese encephalitis virus (JEV), Ilheus virus (ILHV), St. Louis encephalitis virus (SLEV), Powassan virus (POWV), tick borne encephalitis virus (TBEV), Western equine encephalitis virus (WEEV), Eastern equine encephalitis virus (EEEV), Venezuelan equine encephalitis virus (VEEV), Rabies virus (RV), and Borna disease virus-1 (BoDV-1) (Richt et al., 2000; Furr and Reed, 2007c; Carrera et al., 2018; Kumar et al., 2018; Barba et al., 2019; Liesche et al., 2019). Therefore, the horse serves as an indicator species for regional risk of infection and sometimes mirrors similar brain pathologies upon contagion as human patients (David and Abraham, 2016; Kumar et al., 2018; Liesche et al., 2019; Niller et al., 2020).

To understand the pathobiology of neurological diseases and to translate assumptions across species, it is a prerequisite to accurately identify the localization, distribution, and functional and topographic relationship of brain pathologies in the respective species (Nitzsche et al., 2015). To date, the specifics of equine neuroanatomy are featured primarily in topographical literature (Yoshikawa, 1968; Sisson et al., 1975; Nickel et al., 2004; Furr and Reed, 2007b) and studies on specific syndromes describing well-confined brain areas, such as the cerebellar roof and tracts in shivers (Valberg et al., 2015), the hippocampus in BoDV-1 (Joest, 1911) and the cerebellum, brain stem and spinal tracts in case of equine degenerative myeloencephalopathy (EDM) and other types of neuroaxonal dystrophy (Siso et al., 2003; Finno et al., 2011, 2016).

With the implementation of advanced neuroimaging methodologies, neuroanatomy in the field of equine neurology has become relevant for clinicians again, and our functional understanding has steadily increased (Manso-Diaz et al., 2015; Pease et al., 2017). Therefore, imaging has already enabled and supported important clinical-diagnostic (Audigie et al., 2004; Cavalleri et al., 2013; Holmes, 2014), neuroanatomical (Chaffin et al., 1997; Johnson et al., 2019; Schmidt et al., 2019) and neurodevelopmental (Scola et al., 2018) studies in this species. As in other generic groups, magnetic resonance imaging (MRI) scans in particular have proven to be the most sensitive intravital imaging modality (Hecht and Adams, 2010; Holmes, 2014).

Brain imaging templates and atlases rendered via MRI, including diffusion-weighted-imaging (DWI) and fluid-attenuated-inversion-recovery (FLAIR) sequences, have enabled unprecedented mapping and measurement of white matter (WM), gray matter (GM), CSF, and subcortical brain structures (Stuckenschneider et al., 2014; Johnson et al., 2019). The neuroanatomical resemblance has been nicely demonstrated in comparison to tissue studies (Stuckenschneider et al., 2014; Kimberlin et al., 2016; Johnson et al., 2019; Schmidt et al., 2019).

Therefore, researchers can be adequately guided to target affected areas on postmortem follow-up (Stuckenschneider et al., 2014; Schmidt et al., 2019). In spite of this, clinical scanners might provide evidence of brain lesion in only 30% of neurological cases (Manso-Diaz et al., 2015). In particular, failure is likely to occur in slowly progressing neurodegenerative diseases that are accompanied by sparse signal changes and poor contrast enhancement, such as cerebellar cortical degeneration in Arabian horses, which remains unseen until brain atrophy causes increased subarachnoid space (Cavalleri et al., 2013).

While macroanatomical changes coming along with blood brain barrier disruption or critical fluid shifts may easily be diagnosed by medical imaging, subtle tissue changes must await histopathology for definitive diagnosis (Annese, 2012; Cavalleri et al., 2013).

Histological examination, on the other hand, can shed light on a disorder only if the affected area is presented on the slide and cells have been sufficiently preserved. Thus, histology contends with a high risk of sampling bias and artifact (Annese, 2012; Taqi et al., 2018), while MRI studies seemingly provide a gap-free view of the in depth composition of an entire tissue. Prelocalization by MRI could possibly allow postmortem visualization of the lesion or area of interest if predefined external landmarks are preserved, a Cartesian coordinate system (x; y; z) (Nitzsche et al., 2015; Johnson et al., 2019) may be applied, that implements postmortem deformation and shrinkage by fixation, and if the inclination of the blade is guided by a dissection aid adaptable to the geometry of the individual brain. These prerequisites cannot be easily met in a diagnostic lab with personnel heterogeneous in their neuroanatomical skills and dexterity with concomitant time pressure due to high caseloads.

In this study, we aim to provide a freely available, robust, practicable and transferable guide for systematic trimming and sampling of fixed equine brain tissue. This protocol allows sampling of virtually all major functional circuits, vascular territories and pathoclistic^1^ target areas even without specific neuroanatomical knowledge by the applicant. The introduced protocol takes advantage of experiences from the consensus report of the International Veterinary Epilepsy Task Force (IVETF) for sampling epileptic dog and cat brains (Matiasek et al., 2015) after adaption to equine species-specific methods. Thorough neuronavigation is warranted by referring to simple anatomical landmarks supplemented by geometric instructions for blade localization and the plane of section.

By this guidance, brain regions affected by neurological diseases or foci of scientific interest are expected to be reliably and reproducibly traced and provided for histological inspection in a suitable plane, corresponding to the three-dimensional histoarchitecture of specific key areas such as hippocampus. Notably, a detailed knowledge of included areas by the pathologist in the field is not necessary. The rater can be guided remotely to sample the target area simply by referring to the specific slab number.

Moreover, based on this systematic approach, both, population average-based histological data and imaging data could complement each other for the creation of multimodal equine brain atlases and still preserving the optimal slice orientation for histology and histomorphometry.

## Materials and Methods

### Case Selection

The investigation enrolled a cohort of 55 individuals, including mares, geldings and stallions, of various breeds delivered for postmortem examination to the Institute of Veterinary Pathology, LMU, Munich, and the Austrian Agency for Health and Food Safety Ltd. (AGES), Mödling, for causes unrelated to the purpose of this study. Cases were non-selectively collected in a sequential manner if the entire brain tissue was available for examination and if physical preservation allowed for appropriate histoprocessing. Cases were excluded if preservation of the brain was inadequate or if gross lesions interfered with application of all steps of the dissection protocol in both hemispheres.

The study did not lead to a different approach nor to procurement of other or larger volumes of tissue compared to routine autopsy. As the requested diagnostic examinations could be sufficiently performed on the sampled material, the procedures were exempt from Institutional Animal Care and Use Committee review as confirmed by the Ethics Commission of the Centre for Veterinary Clinical Medicine of the LMU Munich (AZ 199-04-02-2020).

### Equipment

**Figure 1:**
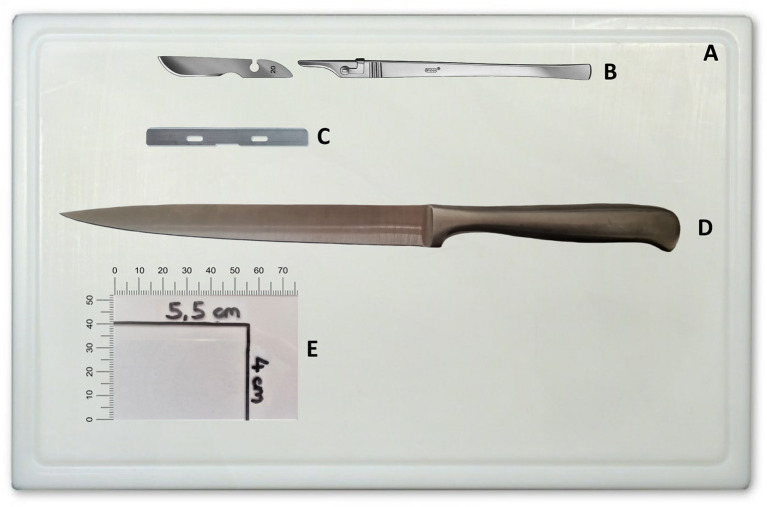
Instruments required for implementation of the protocol. **(A)** Cutting board. **(B)** Scalpel handle and blade. **(C)** Microtome blade. **(D)** Long knife. **(E)** Microscope slide as template/ruler labeled with maximum slab size (herein 4 × 5.5 cm). Depiction of scalpel handle and blade permitted by C. Bruno Bayha GmbH, Tuttlingen, Germany.

The equipment used in this study is the standard equipment ubiquitously available in pathological facilities and is listed in Supplementary Table 1 (Data Sheet 1). Instruments used for conducting the protocol are depicted in Figure 1.

### Gross Procedures

After measurement (≤100kg) or calculation (>100kg) of the dead body weight (DBW), carcasses underwent routine dissection for post-mortem examination (Rooney, 1971; Frank et al., 2015). Following superficial dissection and evisceration, the central nervous system was removed. Thereby, an extensive craniectomy-durotomy-encephalectomy approach was chosen after separation of the head by decapitation at the atlanto-occipital articulation. The exposed brain was evaluated for evidence of autolysis graded as follows: 0: fresh, 1: no macroscopic evidence of autolysis, 2: mild autolysis or 3: moderate autolysis. Marked autolysis (grade 4) and decomposition (grade 5) were considered exclusion criteria. Adult brains were immediately immersed in 10% neutral-buffered formalin [after Lillie (Lillie, 1954)], while those of fetuses and neonates were fixed in zinc formalin [modified by the authors MLB, MR and KM after Fortier and Hould (Fortier and Hould, 2013)]. The tissue to fixative ratio was strictly held at 1:10 (Furr and Reed, 2007a). Brains were left in the fixatives at room temperature for at least 7 days (for details of formulation see Supplementary Table 2, Data Sheet 1).

Just before further processing, brains were removed from the fixative, and excess was allowed to drip-off and was wiped-off using paper towels before the whole brain weight (BW) and brain volume (BV) were recorded. The latter was calculated based on water displacement in a standardized setting. Handling and transport of all specimens corresponded to institutional biosecurity recommendations, and brain dissection was performed at a ventilated pathology bench.

### Development and Introduction of the Protocol

The dissection protocol was elaborated based on collective institutional experiences in equine neuropathology and standardized in analogy to the International Veterinary Epilepsy Task Force (IVETF) guideline for dissection of canine and feline brains (Matiasek et al., 2015). Procedures were adapted to anatomical specificities of the equine brain, such as gyration, brain ratios, orientation and angulation of specific structures and regions (e.g., hippocampus) (Yoshikawa, 1968; Sisson et al., 1975; Nickel et al., 2004; Furr and Reed, 2007b). Thereby, perpendicular depiction of anatomical structures was heeded. The objective was to provide a robust and easy-to-perform protocol, even for inexperienced raters, to ensure reproducible and adequate sampling of virtually all functional subcompartments of the equine brain for both subsequent diagnostic (neuropathological) and neuroscientific investigations.

To facilitate the application, anatomic images and videos were created from six equine subjects in advance of the study. The dissection protocol is introduced in detail in the supplemental attachments (Data Sheets 3–5; Supplementary Videos 1–3).

### Introduction of the Participants

Applicability and aptitude of the protocol *in praxi* were tested in three groups of raters (*n* = 11; all right handed), ranging from undergraduate students (group I; *n* = 4) to personnel with either basic (group II, *n* = 5) or profound (group III, *n* = 2) experience in macroanatomy of the equine brain. All participants were introduced to the approach immediately before conduction of the procedure at the bench. Instruction was assisted by illustrated booklets (Data Sheets 3–5) and instructional videos (Supplementary Videos 1–3).

**Figure 2:**
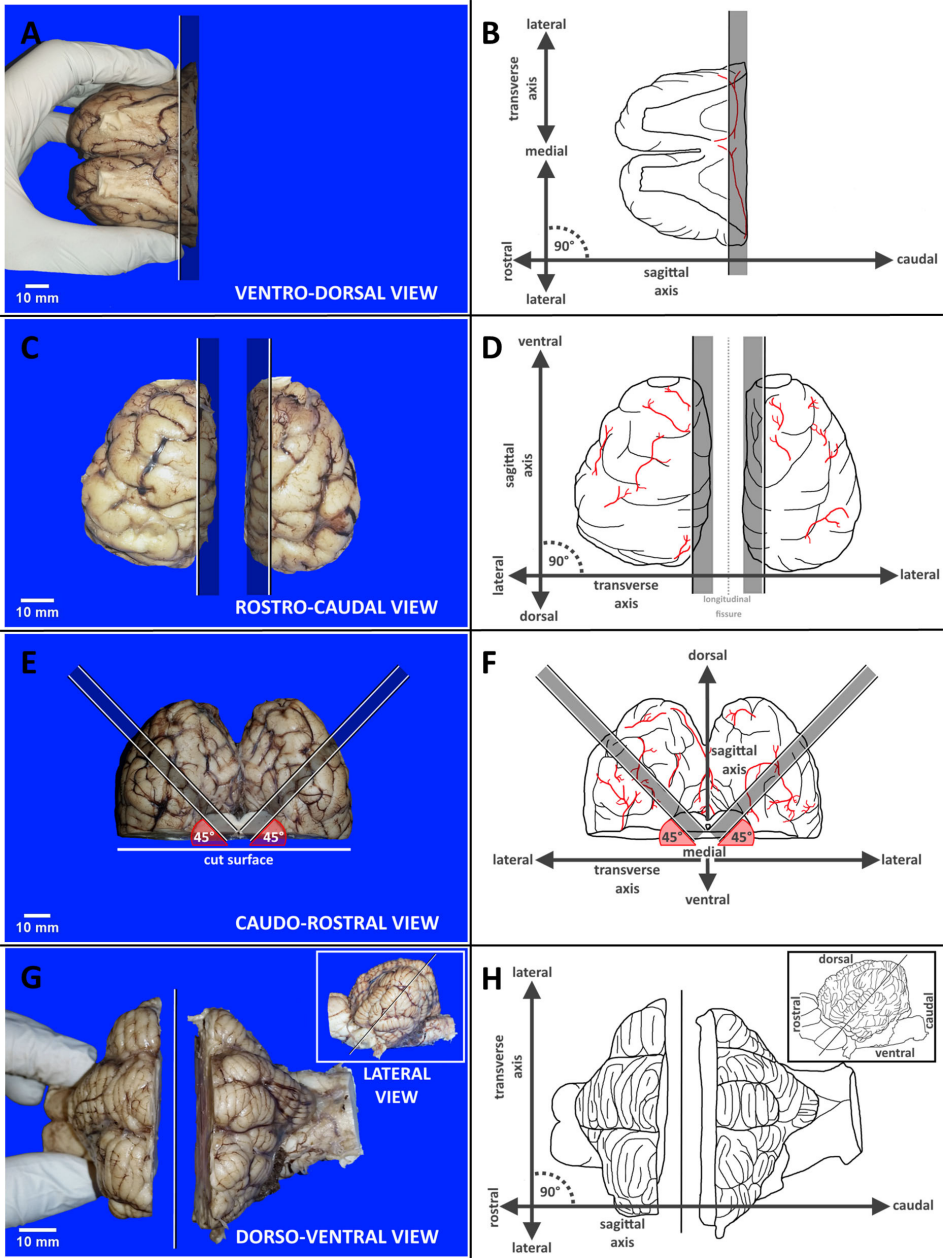
Panel exemplarily demonstrates the different methodologies applied in each brain block from A to D by reference photographs accompanied by schematic illustration with labeled axes supporting orientation. **(A,B)** Transverse dissection of block A. **(C,D)** Sagittal dissection of block B. **(E,F)** Tilted dissection of block C. **(G,H)** Transversely tilted dissection of block D. Black and white lines indicate cutting lines with graying bars highlighting indicated slabs. Red angles target blade inclination. Vessels are shown in red.

First, the investigators were familiarized with the geometric planes and orientations, primary external neuroanatomical landmarks and the equipment required for dissection. Each dissection step was concisely described and depicted by photographs showing the respective region of the brain in relation to the raters' hands and cutting devices. The methodology applied in each brain block is exemplarily illustrated in Figure 2. Essential steps and caveats were listed stepwise in a table and were accompanied by schematic illustrations of important landmarks. Wherever possible, the nomenclature of the current Nomina Anatomica Veterinaria was applied (Nav, 2017). Steps were numbered in the order of their recommended performance, from 1 to 21. Orientation of the cuts was either transverse (TS), sagittal (SAG), laterally tilted (TILT) or transversely tilted (TS-TILT). Positioning and inclination of the blade and direction of the cuts were illustrated using color-labeled anatomical landmarks, while subsequent cuts were explained in simple geometrical terms wherever possible. For simplification of tissue handling, the brains were initially divided into 4 blocks by plain transverse sections. Precise instructions on how to handle and prepare the brain slabs were based on this 4-block-concept. If sliced according to the landmarks, slice to slice distance (equaling thickness of the brain slabs) varied slightly according to the individual brain volumes and dimensions of the hemispheres (see Discussion).

### Implementation of the Protocol

Due to the restricted number of donated brains, the order of procedures was fixed as follows: Each participant performed dissections of 5 brains (trial 1–5), comprising 3 bihemispheric (1st, 4th, and 5th brain) and 2 hemispheric (2nd and 3rd brain, each left and right hemisphere) approaches. Raters were requested to start their first attempt no later than 30 min after self-instruction in the prescribed order and at a pace of their convenience. They were free to reconsult the script during the procedure whenever needed. Participants were observed by the instructor without active interference. Total procedure time (TPT) in minutes, defined as time lapse between first positioning of the blade to the harvest of the last slab, was recorded for each rater. This time only encompassed orientation and hands-on performance. The time needed for macroscopic tissue examination and completion of a questionnaire was excluded from procedure time. For the questionnaire, raters were offered the immediate possibility to mention shortcomings in the comprehensibility and conduction of the protocol and to provide recommendations for further improvement. Moreover, assessment of each unit (block A to D) regarding identifiability of landmarks (1: easy; 2: fair, 3: moderate, 4: not possible) and subjective difficulties in implementation (1: easy, 2: fair, 3: moderate, 4: difficult) were recorded.

### Complementary Parameters

On gross examination, state, preservation and macroscopic changes of the fixed brains were recorded by the instructor. This also included the degree of fixation (1: complete; 2: partial, centrally delayed; 3: poor overall). Measures for artificial damage and lesions were graded as follows: 1: no macroscopic alterations; 2: negligible alterations (e.g., incision marks originating from encephalectomy), 3: moderate alterations (e.g., partial avulsion of regions and structures) and 4: severe alterations (e.g., full avulsion of regions and structures).

### Histoprocessing

Depending on the stage of fixation, a standardized panel of 14 brain slabs was post-fixed in the same types of fixative listed above for further 3–7 days. Specimens were subsequently sent for automatic tissue processing and paraffin embedding. Formalin-fixed paraffin-embedded (FFPE) tissue blocks were then cut in 5–7 μm slice thickness using a rotary microtome. Slides were stained with routine hematoxylin and eosin (H&E) and other neurohistological stains as diagnostically required.

### Brain Slab Validation

The outcome of brain dissections was evaluated by the study leads with respect to neuroanatomical matching and quality of the slides. Evaluation addressed the aptitude for neuroanatomical studies as opposed to simplified routine diagnostic purposes. Analysts were blinded for rater and trial when reading slabs and slides. Neuroanatomical accuracy with respect to levels, structures and orientation of the slide was assessed by matching 36 anatomical and histological landmarks (criteria are listed in Table 1). For each item, sampling accuracy was semiquantitatively scored as either full match (1), partially featured (0.5) or not evident at all (0). Subsequently, whole slab accuracy for detailed neuroanatomical studies was graded as excellent (>80% of landmarks fully featured on the slide), sufficient (70–80% of landmarks fully featured on the slide) or insufficient (<70% of landmarks fully featured on the slide). For visual depiction, verification criteria were further classified in 3 colors: green if 93.1–100% of criteria reached score 1, yellow if 87.3–93% of criteria reached score 1 and red if <87% of criteria reached score 1. Slabs were considered problematic if the relative mean score per slab was <90% within one of the groups. The dissection mode of the 1st slab was categorized in 3 groups: (1) if mainly hippocampus was presented on the slab, (2) if amygdala was the dominant structure of the ventral aspect of the slab, (3) if both structures were available or (4) if none of the criteria were present due to pre-existing tissue artifacts, obliquity or asymmetry of dissection. Apart from the inclusion of marker areas and landmarks, accuracy was further evaluated by assessment of (1) slide symmetry of right vs. left hemisphere and (2) achievement of prescribed cutting angles. Classification of symmetry was considered as either good (1), moderate (0.5) or not present (0) and deviation from prescribed cutting angle as either correct (1), mild deviation (0.5) or severe deviation (0). Slabs per group were considered problematic if <70% of slabs reached a score of 1.

**Table 1:** Demonstrates the 36 macro- and microscopic verification criteria regarding all 14 slabs for evaluation of neuroanatomical accuracy.

**Block**	**Slab**	**Criterion**	**Anatomical and histological landmarks**
A	1	C1	Hippocampal (syn. fornical) commissure
A	1	C2	Hippocampal temporoventral body (TVB)
A	1	C3	Piriform cortex and amygdaloid nucleus
A	1	C4	Rostral cerebral crus (crurocapsular transition)
A	1	C5	Mammillary bodies
A	2	C6	Parietooccipital cingulate gyrus
	2	C7	Rostral (anterior) ventral nucleus
A	2	C8	Tail of caudate nucleus
A	2	C9	Optic tract (postchiasmatic segment)
A	3	C10	Accumbens nucleus
A	3	C11	Body of caudate nucleus
A	3	C12	Globus pallidus
A	3	C13	Putamen
A	3	C14	Internal capsule (rostral part)
B	4	C15	Subcortical white matter of marginal gyrus and of precruciate cingulate gyrus
B	5	C16	Perpendicular section of rostral composite gyrus
B	5	C17	Betz cells (microscopic) of frontal motor cortex
C	6	C18	Lateral geniculate nucleus
C	6	C19	Hippocampal temporoventral body (TVB)
C	7	C20	Occipital vertex of hippocampus
C	7	C21	Occipital apex of parahippocampal gyrus
C	8	C22	Subcortical white matter of marginal gyrus (occipital part) and of cingulate gyrus (cinguloparahippocampal transition)
C	8	C23	Occipital alveus of hippocampus
C	8	C24	Splenial sulcus
D	9	C25	Rostral colliculi
D	9	C26	Intercrural fossa
D	10	C27	Caudal and middle cerebellar peduncles
D	10	C28	Transverse fibers of pons
D	10	C29	Lingula of vermis
D	10	C30	Cerebellar roof nuclei (syn. deep cerebellar nuclei)
D	11	C31	Medial plane of rostral vermis
D	12	C32	Decussation of pyramis
D	13	C33	Cuneate and gracile nuclei
D	13	C34	Vagal and hypoglossal nuclei
D	14	C35	Mid sagittal uvula
D	14	C36	Area postrema

### Statistical Analysis

Analyses were performed using IBM SPSS Statistics and GGraph commercial software (Version 26; IBM Corp., Armonk, New York, USA) and were based on uni- and bivariate statistics. Data for each correlation was analyzed for normality using a Shapiro-Wilk test or Kolmogorov-Smirnov test. Independence was evaluated by Chi-squared test. Correlations were performed using a Pearson correlation for normally distributed parametric data and a Kendall's rank (Kendall-Tau-b) correlation coefficient, as well as Mann-Whitney *U*-test, for non-normally distributed non-parametric data. Somers' D was used as a measure of agreement between pairs of ordinal variables. For identification of significant findings, data were analyzed using one-way ANOVA followed by Dunnett's *post-hoc* test to identify pairs with significant differences. Comparison of related samples was performed by Wilcoxon test. The extent of variability in relation to the mean of the population was assessed by the coefficient of variation. As most values were not normally distributed, data are presented as the mean ± SD (standard deviation) or median and interquartile range (IQR, 25th and 75th percentiles). Percentages describe discrete data. For visual depiction of non-parametric data, box and whisker plots were used. Values of *p* ≤ 0.05 were considered significant.

## Results

### Demographics

The investigated cohort included 17 mares (30.9%), 26 geldings (47.3%), and 12 stallions (21.8%) aged between 2 months ante partum (abortion) and 25 years. Seven individuals were adults of unknown age. Breeds encompassed Warmbloods (*n* = 37, 67.3%), Ponies (*n* = 9, 16.4%), Thoroughbreds (*n* = 3, 5.5%), Arabian horses (*n* = 2, 3.6%), and Draft horses (*n* = 2, 3.6%). Two animals (*n* = 2, 3.6%) belonged to other equine species. Tissue preservation was graded as fresh (*n* = 15, 29.1%), without macroscopic evidence of autolysis (*n* = 20, 36.4%), mild autolysis (*n* = 15, 27.3%), and moderate autolysis (*n* = 4, 7.3%). Details regarding the cases are shown in Supplementary Table 3 (Data Sheet 2).

**Figure 3:**
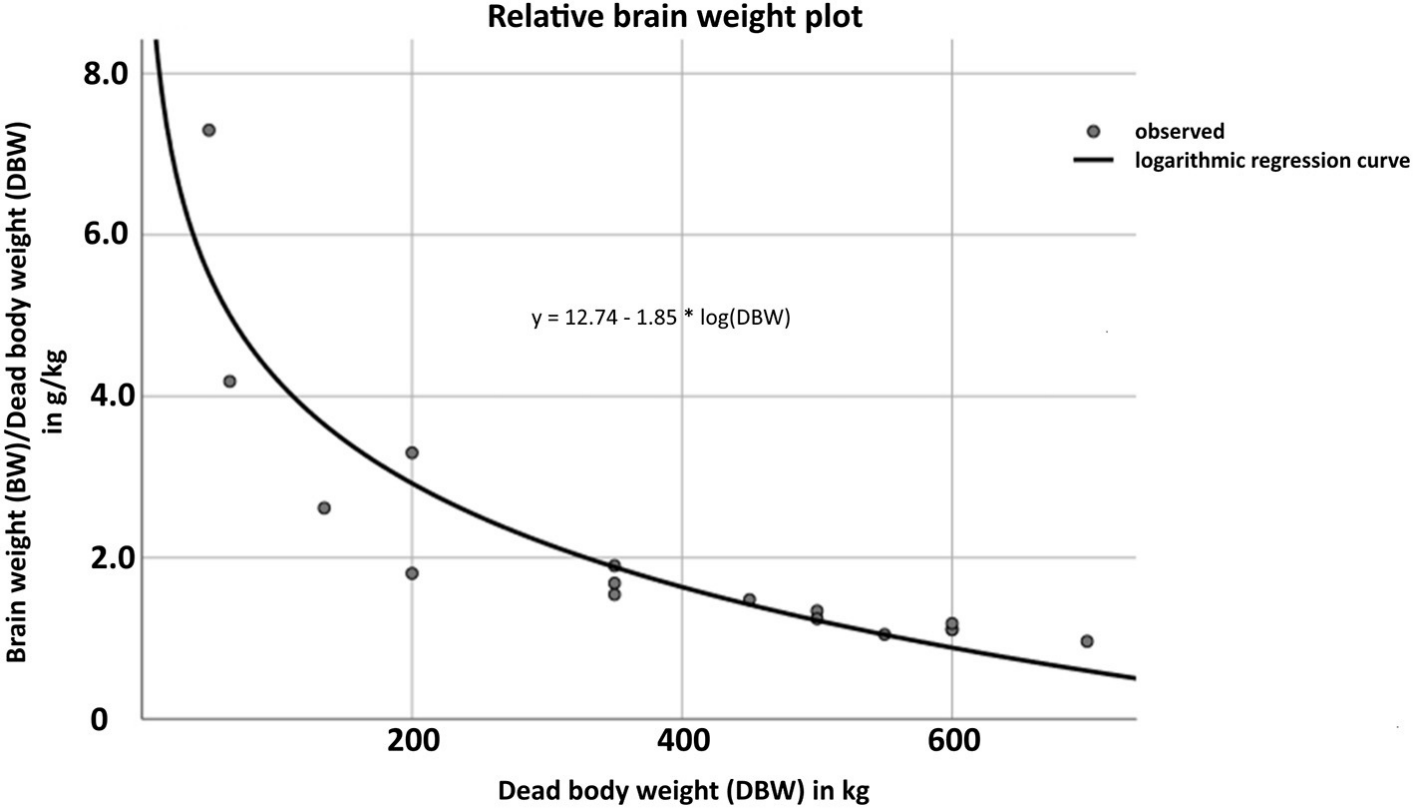
Demonstration of the logarithmic, non-linear relationship (logarithmic regression curve) between dead body weight (DBW) in kg (x-axis) and brain weight (BW) post fixation in g (y-axis) according to the formula *y* = 12.74 – 1.85 * log(DBW). Details of individual cases are shown in Supplementary Table 4 (Data Sheet 2).

Weight and volume measurements were available for 22 cases (*n* = 22, 40%). The median dead body weight (DBW) was 275 kg (*n* = 22, IQR: 43.7–512.5), while median drip-off weight of whole brains post fixation (BW) was 558 g (*n* = 22, IQR: 353–665.3), and median brain volume (BV) was 625 cm^3^ (*n* = 22, IQR: 395–745). Based on these data, the relationship between BW and DBW was evaluated and presented in a non-linear, logarithmic fashion (Figure 3). Respective details of individual cases are shown in Supplementary Table 4 (Data Sheet 2).

### Dissection Outcome

**Figure 4:**
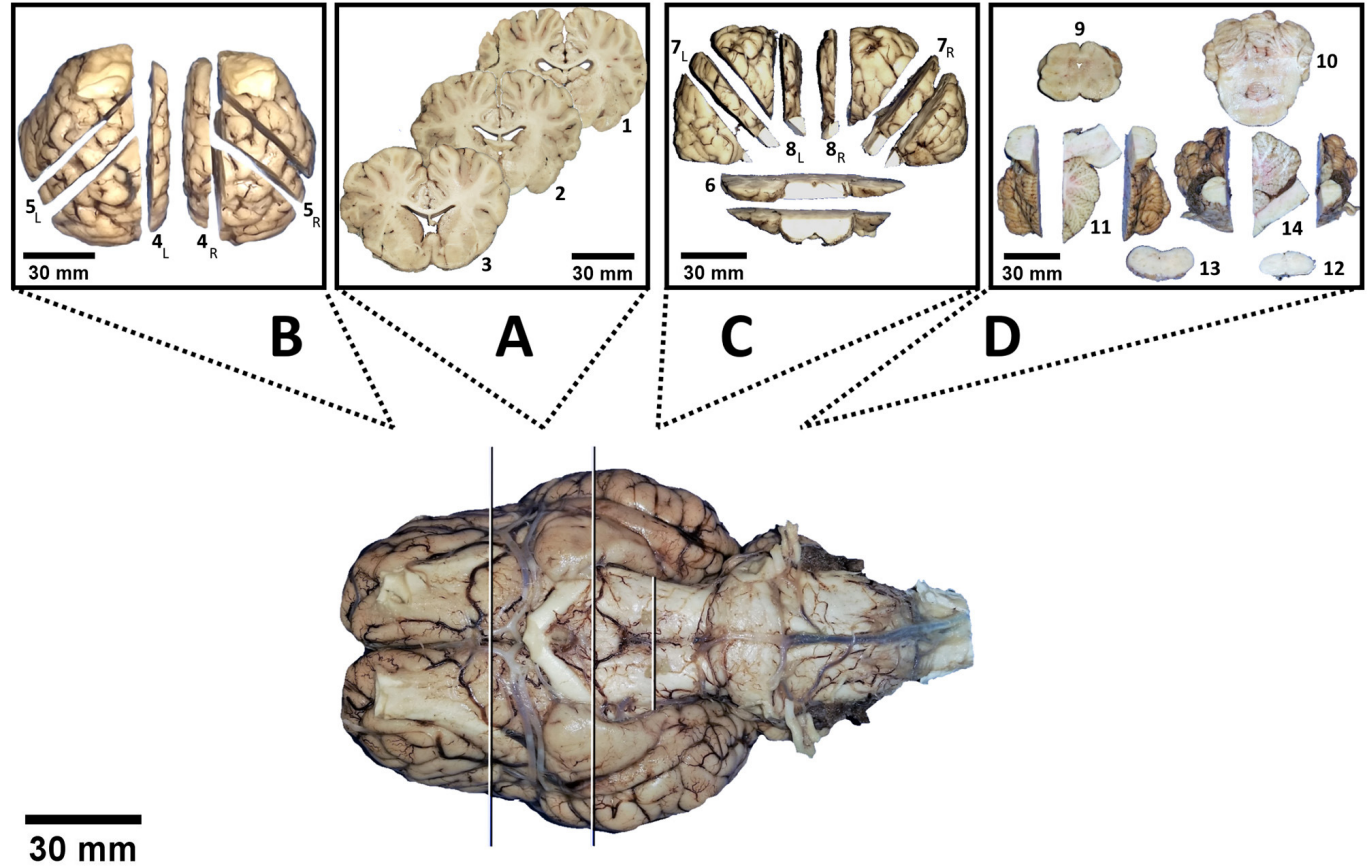
Virtual division of the brain into 4 blocks (white and black lines). Following dissection according to the protocol, each block delivers a certain amount of slabs depicted in a separate box: No. 1 to 3 out of block **(A)**, No. 4 to 5 out of block **(B)**, No. 6 to 8 out of block **(C)** and No. 9 to 14 out of block **(D)**. Subscript letters indicate distinctions between left (L) and right (R) hemispheres.

Application of the bihemispheric protocol delivered 14 brain slabs of 4 main brain blocks: slabs No. 1 through 3 out of block A, No. 4 and 5 out of block B, No. 6 through 8 out of block C, and No. 9 through 14 out of block D (Figure 4). In contrast, hemispheric dissection delivered 28 slabs in total (14 per hemisphere).

### Conduction Time

**Figure 5:**
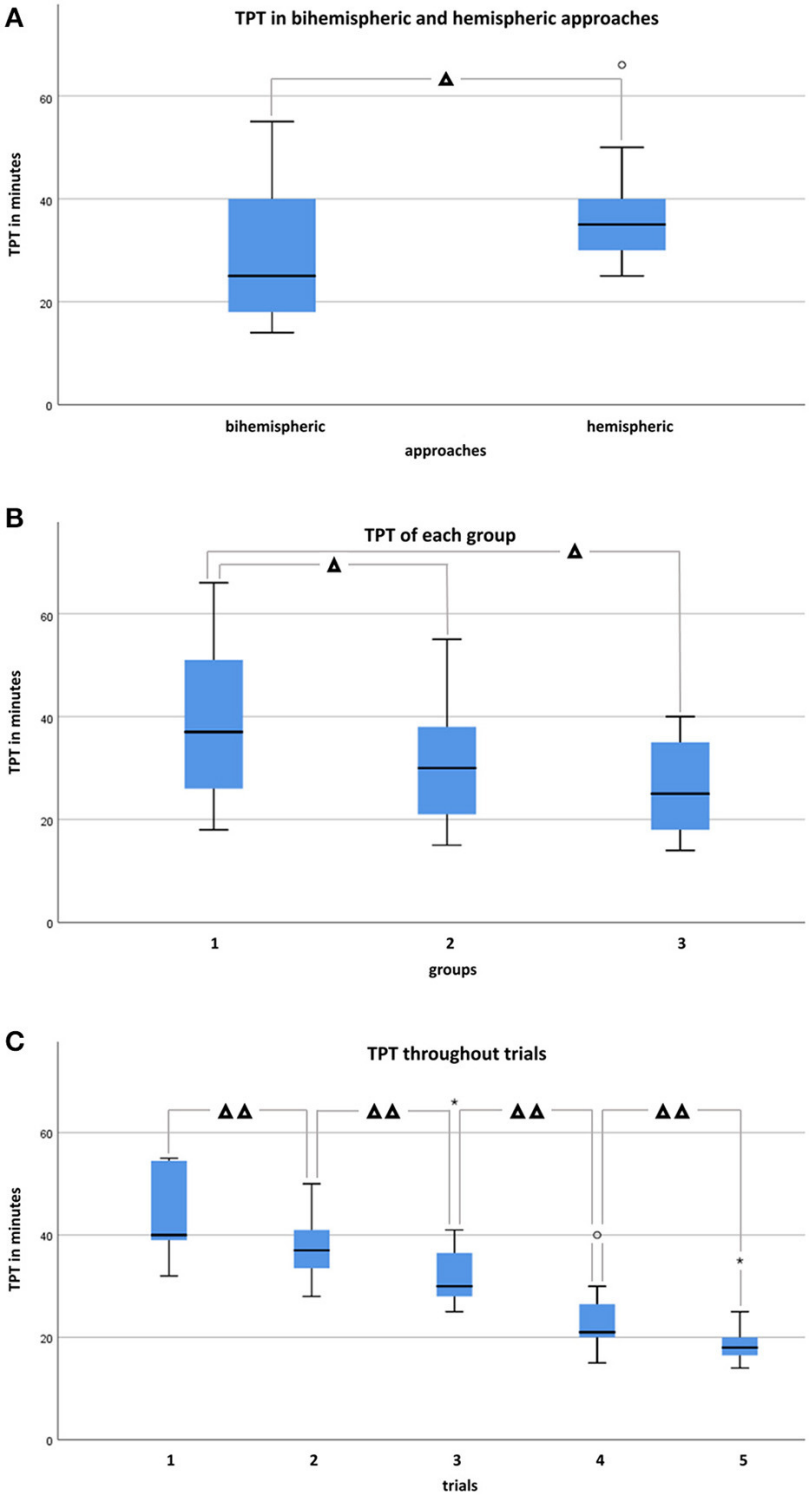
Demonstrates confrontation of total procedure times (TPT) in minutes in box and whisker plots between bihemispheric and hemispheric approaches, levels, and trials. **(A)** TPT in bihemispheric (*n* = 33) and hemispheric (*n* = 22) tailored brains throughout groups. Mann-Whitney-*U*-test identified a statistically significant difference between bihemispheric and hemispheric approaches if considered for all groups (*p* < 0.05), but significance was not achieved if groups were considered individually (group I, *n* = 20, *p* = 0.296; group II, *n* = 25, *p* = 0.060; group III, *n* = 10, *p* = 0.386). **(B)** TPT throughout approaches and trials of each group. Experienced raters performed faster than the less experienced group (One-way ANOVA, *post-hoc* Dunnett-T) if group III (*n* = 10) and I (*n* = 20), as well as group II (*n* = 25) and I (*n* = 20) were compared to each other (*p* < 0.05), but not (*p* = 0.999) if comparison was performed between group III (*n* = 10) and II (*n*= 25). **(C)** TPT throughout groups significantly decreased (Kendall-Tau-b = −0.663, *p* < 0.001) from trials 1 (*n* =11) to 5 (*n* = 11). °Indicates individual Outliers and *Extremes. Significant differences are indicated with Δ for *p* < 0.05 and with ΔΔ for *p* < 0.001.

Total procedure time (TPT) throughout trials and groups ranged between 14 and 55 min (median 25, IQR: 18–40) for bihemispheric (*n* = 33) and 25–66 min (median 35, IQR: 29.5–40.25) for sequential hemispheric (*n* = 22) dissections. There was no significant difference in TPT between bihemispheric and hemispheric approaches within the individual skill groups [group I (Mann-Whitney-*U, p* = 0.296); group II (Mann-Whitney-*U, p* = 0.060); group III (Mann-Whitney-*U, p* = 0.386)]. In contrast, throughout all raters, dissection of the whole brain went significantly faster (Figure 5A; Mann-Whitney-*U, p* < 0.05). The time difference between cutting of left and right hemisphere throughout groups was significant (Wilcoxon-test, *p* < 0.001), with the right hemisphere being tailored faster than the left. In accordance with individual experiences, median TPT throughout the trials was 37 min in group I (IQR: 25–51.5), 30 min in group II (IQR: 20.5–38), and 25 min in group III (IQR: 17.3–35). TPT trended down with increasing skill of the investigator (Figure 5B; Kendall-Tau-b = −0.286, *p* < 0.05; One-way ANOVA, *p* < 0.05). However, an effect of neuroanatomical skill on velocity of trimming was significant between groups III and I (*post-hoc* Dunnett-T = −12.350, *p* < 0.05) and groups II and I (*post-hoc* Dunnett-T = −8.130, *p* < 0.05), where experienced raters performed faster than less experienced raters. All raters, independent of their onset skill, level became faster upon repetition of the protocol in consecutive sessions (Figure 5C; Kendall-Tau-b = −0.663, *p* < 0.001). Relative saving in TPT between the first (range 32–55 min, median 40, IQR: 38–55) and the last trial (range 14–35 min, median 18, IQR: 15–21) was 25.45 min on average, independent of the time span between the trials. Notably, acceleration across raters did not negatively impact anatomical accuracy (Pearson = −0.332, *p* < 0.05). For details of individual TPTs, see Supplementary Table 5 (Data Sheet 2).

### Brain Slab Accuracy

**Figure 6:**
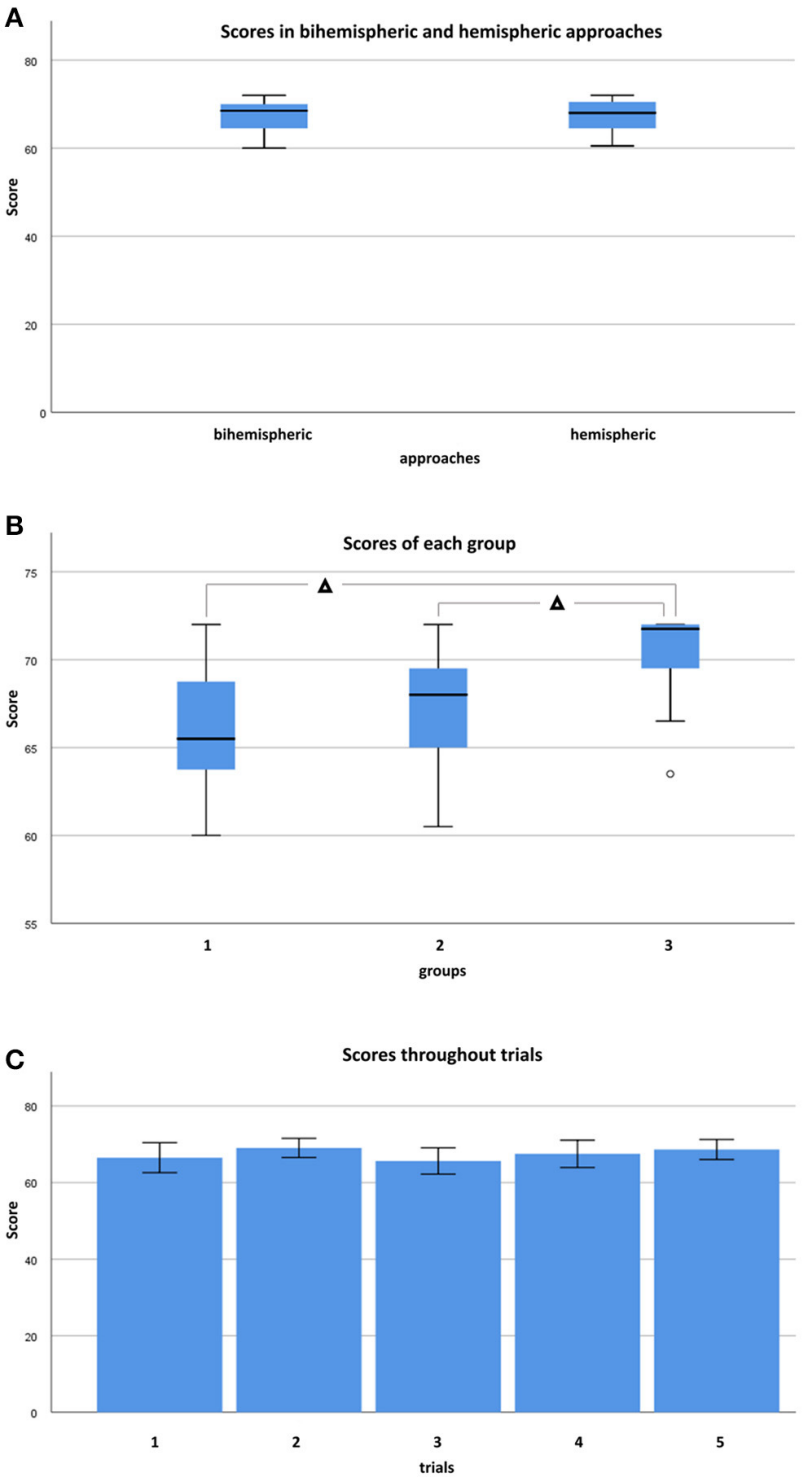
Demonstrates confrontation of scores in box and whisker plots between bihemispheric and hemispheric approaches, levels, and trials. **(A)** Scores in bihemispheric (*n* = 33) and hemispheric (*n* = 22) tailored brains throughout groups. Mann-Whitney-*U*-test found no statistically significant difference between bihemispheric and hemispheric approaches if considered for all groups (*p* = 0.776). **(B)** Scores throughout approaches and trials of each group. Experienced raters scored higher than less experienced raters (One-way ANOVA, *post-hoc* Dunnett-T) if group III (*n* = 10) and II (*n* = 25), as well as group III (*n* = 10) and group I (*n* = 20) were compared to each other (*p* < 0.05), but not (*p* = 0.429) if comparison was performed between group II (*n* = 25) and group I (*n* = 20). **(C)** Almost consistent scores from trials 1 (*n* = 11) to 5 (*n* = 11) without significant differences among skill groups (Kendall-Tau-b = 0.074, *p* = 0.470). °Indicates individual Outliers. Significant differences are indicated with Δ for *p* < 0.05.

Across all raters, neuroanatomical accuracy score per brain was never below 60 out of 72 (maximum) points. Median accuracy throughout trials and raters was 68 points (IQR: 64.5–70.5). Inexperienced raters in group I achieved median scores of 65.5 points (IQR: 63.6–68.9), more experienced raters in group II achieved median scores of 68 points (IQR: 64.8–69.5), and experienced investigators in group III scored a median of 71.8 points (IQR: 68.8–72). Total median scores throughout trials and groups were 68.5 (IQR: 64.25–70.5) for bihemispheric and 68 (IQR: 64.6–70.5) for sequential hemispheric dissections. Therefore, differences in scoring between bihemispheric and hemispheric dissections did not reach a level of significance (Figure 6A; Mann-Whitney-*U, p* = 0.776). Neuroanatomical accuracy scores were not significantly different between left and right hemisphere (Wilcoxon-test, *p* = 0.832). However, scores were significantly higher in groups with higher levels of neuroanatomical skill (Kendall-Tau-b = 0.328, *p* < 0.05). If groups were compared to each other (One-way ANOVA, *p* < 0.05), scores were significantly higher in group III vs. II (*post-hoc* Dunnett-T = 2.93, *p* < 0.05) and in group III vs. I (*post-hoc* Dunnett-T = 4.00, *p* < 0.05), but not in group II vs. I (*post-hoc* Dunnett-t = 1.07, *p* = 0.429) participants (Figure 6B). Increasing experience with the protocol from the first (trial 1) to the last (trial 5) dissected brain had no significant influence on attained scores throughout different skill groups (Figure 6C; Kendall-Tau-b = 0.074, *p* = 0.470).

Intrarater scores ranged from 0 to 8.67%. The variation coefficient was not significantly dependent on the skill group (Kendall-Tau-b = −0.306, *p* = 0.234). For details of individual scores, see Supplementary Table 6 (Data Sheet 2).

**Figure 7:**
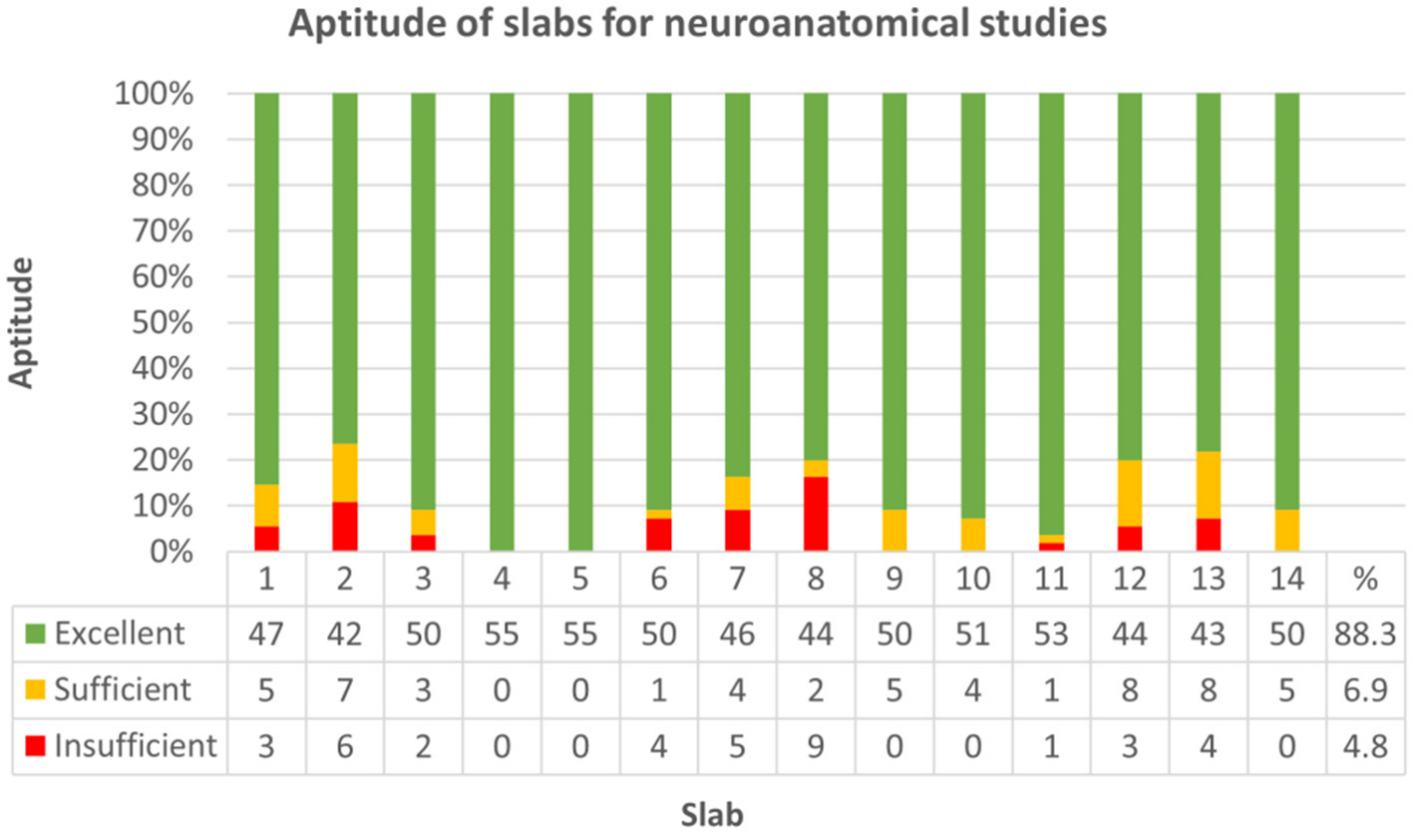
Demonstrates aptitude of slabs for neuroanatomical studies by reference to 36 criteria (see Table 1) across all groups. Strikes of anatomical landmarks were scored as either full match (1), partly featured (0.5) or not evident at all (0), and slabs were graded as excellent (green; >80% of landmarks fully featured on the slide), sufficient (yellow; 70–80% of landmarks fully featured on the slide) or insufficient (red; <70% of landmarks fully featured on the slide) for neuroanatomical studies. 680 out of 770 slabs (88.3%) were considered excellent, 53/770 (6.9%) sufficient and 37/770 (4.8%) failed to show the minimum panel of anatomical structures at the cut surface.

**Figure 8:**
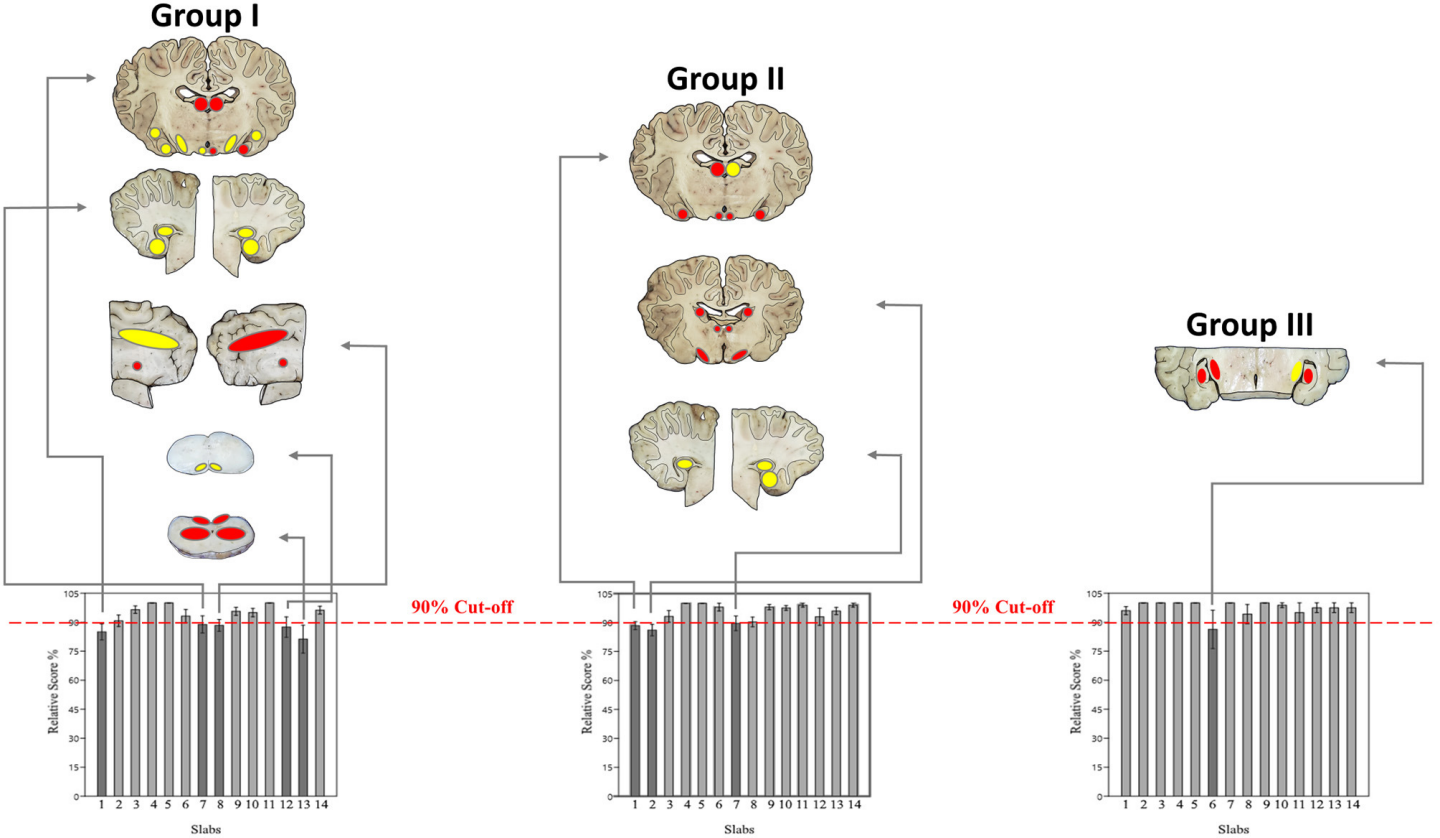
Demonstrates the most problematic slabs per group with a mean relative score <90% (dark gray). Matching of macroscopic and microscopic verification criteria for evaluation of slabs for neuroanatomical accuracy (Table 1) were classified in 3 colors: green if 93.1–100% of criteria were scored with 1 (slabs not shown), yellow if 87.3–93% of criteria were scored with 1 and red if <87% of criteria were scored with 1. Concerning each group, the following slabs underscored 90%: slabs 1, 7, 8, 12, and 13 in group I; slabs 1, 2, and 7 in group II; slab 6 in group III.

If sorted for slabs, 680/770 (88.3%) were considered excellent, 53/770 (6.9%) sufficient, and 37/770 (4.8%) failed to show the minimum panel of anatomical structures at the cut surface (Figure 7; Supplementary Table 7, Data Sheet 2). Concerning each individual group, slabs 1, 7, 8, 12, and 13 in group I, slabs 1, 2, and 7 in group II and slab 6 in group III remained under a mean relative score of 90% and were therefore considered the most problematic in the respective groups (Figure 8). Ranges of scores for each slab per group in absolute and relative numbers are shown in Supplementary Table 8 (Data Sheet 2).

Regarding the dissection mode of the 1st slab, on left hemispheric slabs, the hippocampal type was the most frequent form [21/55 (38.2%)], followed by the amygdaloid type [20/55 (36.4%)] and hybrid type [10/55 (18.2%)]. On right hemispheric slabs, the predominant form was the amygdaloid type [23/55 (41.8%)], followed by the hybrid type [18/55 (32.7%)] and hippocampal type [10/55 (18.2%)]. Whereas, in hemispheric approaches [22/55 (40%)], hippocampal, amygdaloid, and hybrid type were equally represented, in bihemispheric approaches [33/55 (60%)], the amygdaloid type was the most frequent form [31/66 (47%)], followed by the hippocampal type [19/66 (28.8%)] and hybrid type [16/66 (24.2%)]. In 4 brains (18.2%) of hemispheric approaches, none of the criteria were present.

**Figure 9:**
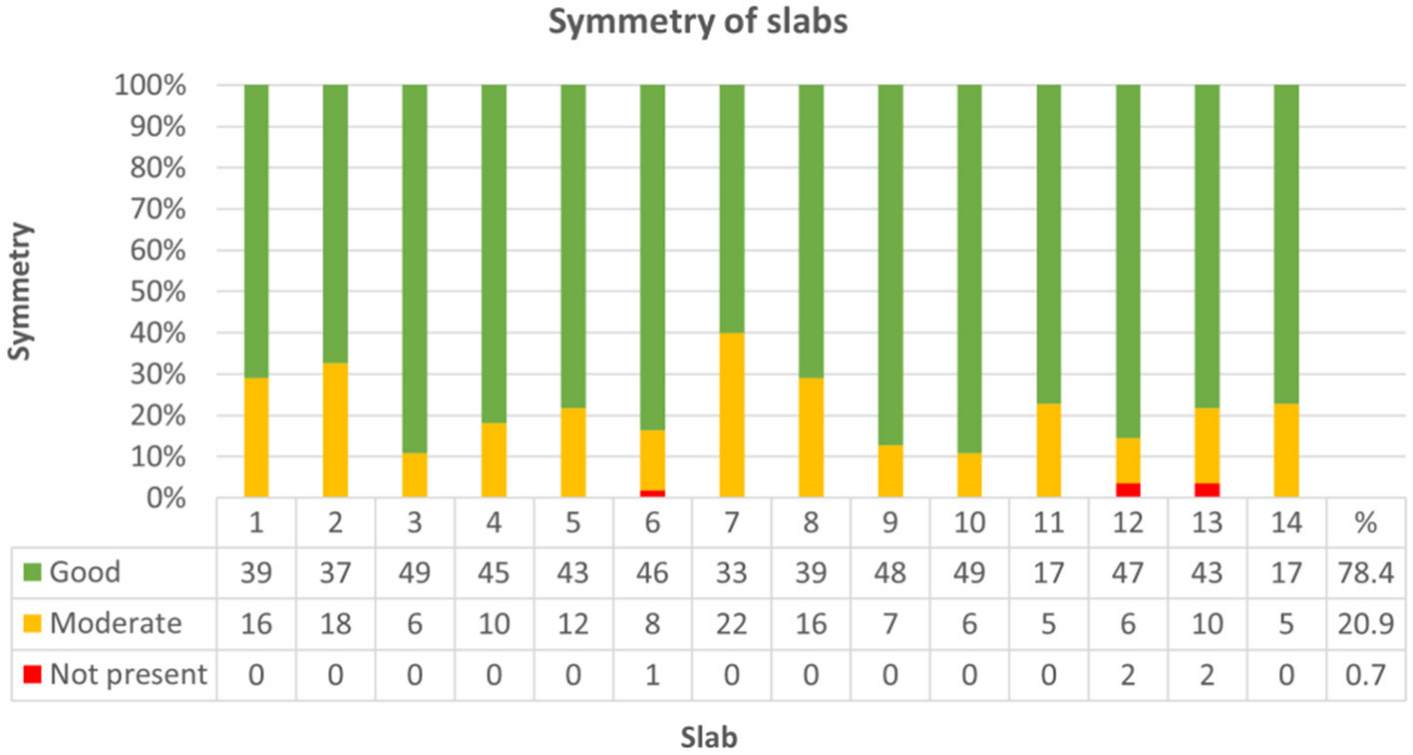
Demonstrates classification of slabs regarding symmetry across all groups. Symmetry was graded as follows: good (1), moderate (0.5) or not present (0). In 552 out of 704 slabs (78.4%) symmetry was good (green), moderate (yellow) in 147/704 (20.9%), and not present (red) in 5/704 (0.7%) of slabs.

Throughout groups, symmetry was assessed in 704 of 770 slabs. Resembling sagittal midline slabs, slab no. 11 and 14 were excluded from this analysis. Symmetry did not significantly differ between bihemispheric and hemispheric dissection (Chi-Square = 0.394, *p* = 0.821). There was a weak correlation between higher levels of neuroanatomical skill and grade of symmetry (Kendall-Tau-b = 0.082, *p* < 0.05) and a moderate correlation between symmetry and higher neuroanatomical scores (Kendall-Tau-b = 0.143, *p* < 0.001). Increasing experience with the protocol from the first trial (1) to the last trial (5) had no significant influence on symmetry of slabs in any of the groups (Kendall-Tau-b = 0.027, *p* = 0.431). Symmetry was graded as good in 78.4% (552/704) of slabs, as moderate in 20.9% (147/704) and as not present in 0.7% (5/704) of slabs (Figure 9; Supplementary Table 9, Data Sheet 2). Concerning each individual group, most problematic slabs regarding symmetry were slabs 4, 7, and 8 in group I, slabs 1, 2, and 7 in group II and slab 7 in group III (Supplementary Table 10, Data Sheet 2).

**Figure 10:**
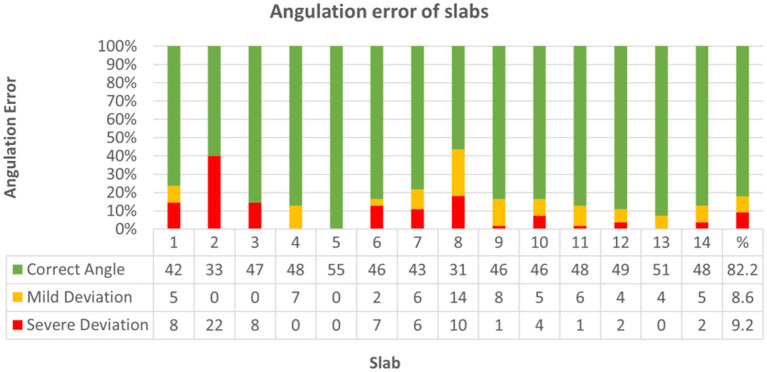
Demonstrates classification of slabs regarding angulation error from prescribed cutting angle across all groups. Angulation was graded as follows: correct angle (1), mild deviation (0.5) or severe deviation (0). In 633 out of 770 slabs (82.2%) cutting angle was correct (green), mildly deviated (yellow) in 66/770 (8.6%), and severely deviated (red) in 71/770 (9.2%) slabs.

Deviation did not significantly differ between bihemispheric and hemispheric dissection (Chi-Square = 2.17, *p* = 0.338). Correct inclination mildly correlated with neuroanatomical skill (Kendall-Tau-b = 0.088, *p* < 0.05) and strongly correlated with higher scores (Kendall-Tau-b = 0.497, *p* < 0.001). Increasing experience with the protocol from the first trial (1) to the last trial (5) had no significant influence on deviation from correct angle throughout skill groups (Kendall-Tau-b = 0.056). Throughout all groups, cutting angle was correct in 82.2% (633/770), mildly deviated in 8.6% (66/770), and severely deviated in 9.2% (71/770) of slabs (Figure 10; Supplementary Table 11, Data Sheet 2). Concerning each individual group, most problematic slabs regarding deviation from the prescribed cutting angle were slabs 7 and 8 in group I, slabs 2 and 8 in group II and slab 2 in group III (Supplementary Table 12, Data Sheet 2).

### Effects of Tissue Preservation on Neuroanatomical Accuracy

**Figure 11:**
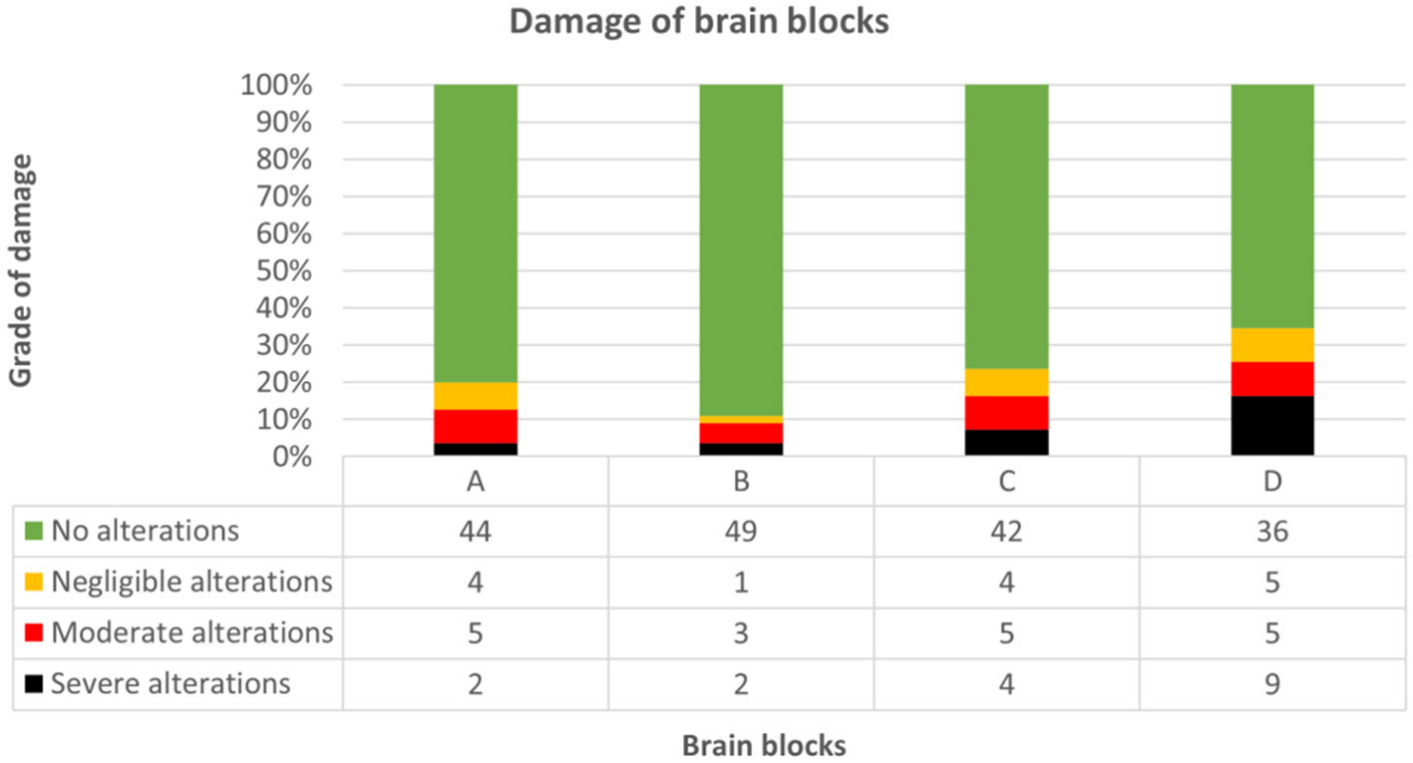
Demonstrates grade of damage concerning each brain block throughout groups. Block D was most prone to artifacts [19/55 (34.5%)], followed by blocks C [13/55 (23.6%)], A [11/55 (20%)] and B [6/55 (11%)]. Pre-existing defects ranged from no (green), to negligible (yellow), moderate (red) and severe (black) alterations (for details see “complementary parameters”).

Regarding tissue preservation, neither autolysis (Kendall-Tau-b = 0.400, *p* = 0.192) nor subfixation (Kendall-Tau-b = 0.133, *p* = 0.435) impacted neuroanatomical accuracy score significantly. Pre-existing defects of brain tissue attending neurodissection were evident in 34.5% (19/55) of brains, with block D being most prone to artifacts [slabs 9–14; 19/55 (34.5%)] followed by blocks C [slabs 6–8; 13/55 (23.6%)], A [slabs 1–3; 11/55 (20%)] and B [slabs 4–5; 6/55 (11%)] (Figure 11). In general, brain extraction artifacts were seen in 10/20 (50%) brains of group I, 12/25 (48%) of group II, and 3/10 (30%) of group III.

According to expectations, there was a strong correlation between higher grades of damage and lower scores (Kendall-Tau-b = 0.87, *p* < 0.05). Although pre-damage extinguished one or more markers of orientation in any of the blocks, significantly lower scores caused by missing reference points were only observed in block C (Kendall-Tau-b = −0.299, *p* < 0.05), in contrast to blocks A (Kendall-tau-b = −0.198, *p* = 0.075), B (Kendall-tau-b = −0.190, *p* = 0.091) and D (Kendall-tau-b = −0.134, *p* = 0.217). If damage was mild, there was a positive correlation between skill level and neuroanatomical accuracy (Kendall-Tau-b = 0.173, *p* < 0.001). However, advanced brain damage leveled out the positive influence of neuroanatomical skills (moderate damage: Kendall-Tau-b = −0.056, *p* = 0.650; severe damage: Kendall-Tau-b = 0.019, *p* = 0.865; high-grade damage: Kendall-Tau-b = −0.006, *p* = 0.955).

### Rater Data Assessment

**Figure 12:**
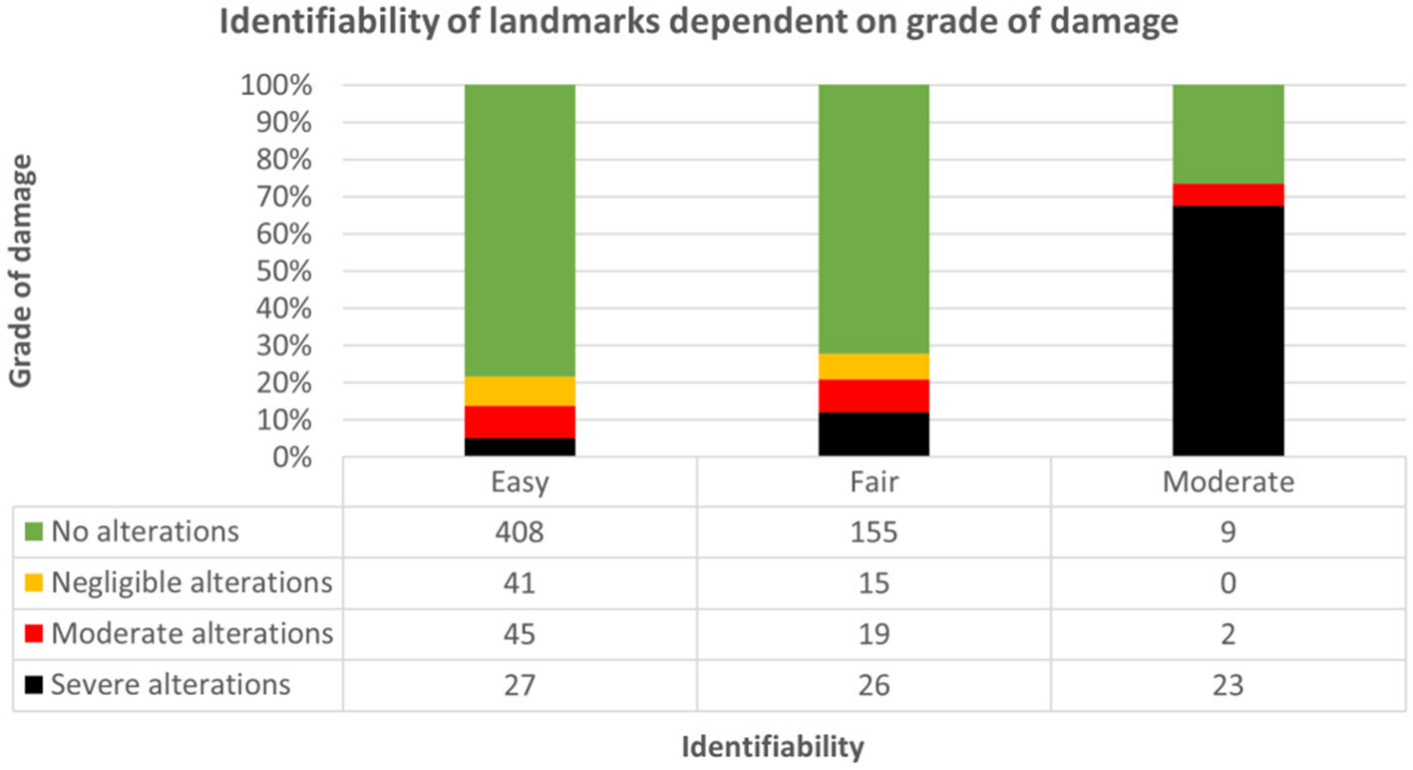
Demonstrates dependence of identifiability of landmarks (1: easy; 2: fair; 3: moderate; 4: not possible) on grade of damage throughout groups and approaches. Identifiability was easiest in slabs without alterations [green; 408/521 (78.3%)], followed by moderate [red; 45/521 (8.6%)], negligible [yellow; 41/521 (7.9%)] and severe [black; 27/521 (5.2%)] alterations.

According to the questionnaire taken by participants following every dissection, identifiability of relevant landmarks was easiest in brains without macroscopic alterations in groups I (70.3%), II (82%), and III (80.7%). However, orientation of the blade was possible in brains even with severe alterations and impossible in none of the altered brains (Figure 12; Supplementary Table 13, Data Sheet 2). Therefore, the method of dissection (bihemispheric vs. hemispheric) was not pivotal for identifiability (Somers' D = 0.034, *p* = 0.370). The lower the grade of damage, the better the identifiability of landmarks (Somers' D = 0.294, *p* = < 0.001); however, the relationship between grade of damage and identifiability of landmarks was significant only in blocks A (Somers' D = 0.330, *p* < 0.05) and D (Somers' D = 0.320, *p* < 0.05). Increasing subjective difficulties with implementation of the protocol were significantly related to increasing grade of tissue damage in groups I (Somers' D = 0.197, *p* < 0.05) and II (Somers' D = 0.141, *p* < 0.05) and therefore decreasing identifiability of landmarks in group I (Somers' D: 0.443, *p* < 0.001) and group II (Somers' D: 0.591, *p* < 0.001), whereas members of group III did not perceive difficulties regarding application of the protocols.

Subjective difficulties did not reflect the neuroanatomical accuracy in groups I and II (group I = Kendall-Tau-b: −0.041, *p* = 0.469; group II = Kendall-Tau-b: −0.087, *p* = 0.100). The comfort levels increased with the training effect in group I (Somers' D: −0.127, *p* < 0.05), but not in group II (Somers' D: −0.096, *p* = 0.132). Subjective difficulties were similar for application of bihemispheric and hemispheric protocols (Somers' D = 0.008, *p* = 0.819).

## Discussion

### Ambition of the Protocol

Unfortunately, for inexperienced researchers and diagnosticians, no standardized or revised guidelines for systematic and reproducible dissection of the equine brain has been available prior to this study. Empirically, people tend to use transverse sections as traditional planes, which might impede concise evaluation and histomorphometry of regions that are presented in tangential cuts, such as major aspects of the hippocampus and motor cortex or those with peculiar planarity, such as the cerebellar cortex.

Only by consequent use of the same scheme researchers are able to acquire a macroscopical and histological pattern recognition for normal and abnormal fields. Moreover, only by transferable protocols it is possible to compare architectural and quantitative tissue data of the large gyrified equine brain reliably between different studies and laboratories.

The protocol presented herein has been elaborated to facilitate macroscopic evaluation and procurement of standardized target areas in equids. This study proved that by application of this protocol, investigators can sample virtually every functional subsystem of the equine brain at optimal angles independent of their neuroanatomical skills. Hence, the landmarks for orientation of sections have been kept as simple as possible.

### Study Design

To facilitate handling throughout the different modes of encephalectomy and tissue availability, the protocol presented herein was developed for bihemispheric and hemispheric (left & right) dissection (see Data Sheets 3–5 and Supplementary Videos 1–3). All steps are illustrated in a video instruction of 14 min (bihemispheric) and 10 min (hemispheric, each) duration (see Supplementary Videos 1–3). Equipped with these aids, the examiners who volunteered for this study all felt prepared to proceed to the hands-on experiments.

### Time Requirement for Implementation

After no more than 5 repeats, all raters throughout experience levels were able to sample all major brain areas in ~20 min or less. On average, they saved 26 min from the first to the fifth dissected brain. With more samples treated the same way, investigators will be able to decrease their performance time to that of the videos or even below. It remains to be seen, however, whether this only reflects the time to cut the brain in this standardized manner, while thorough macroscopic examination of the entire brain and of the slabs requires time on its own. Most notably, procedural acceleration had no negative effects on the neuroanatomical accuracy of sampling across all raters and therefore reflects quickly increasing experience and straightforward practicability.

Surprisingly, although all investigators were right-handed, an anticipated faster and more accurate dissection of the left hemisphere due to easier handling was absent. Dissection of the right hemisphere was performed significantly faster upon steady sampling quality. This time difference could not be explained by brain asymmetry due to right forebrain predominance in equids as previously reported (Larose et al., 2006; Austin and Rogers, 2007; Farmer et al., 2010, 2018; Johnson et al., 2019).

Instead, this unexpected outcome was likely due to predefined chronology of the study protocol, in which the dissection of the left hemisphere always preceded that of the right. A sequential order was required, as restricted availability of donated brains rendered a randomized order less feasible.

### Diagnostic Validity

Aptitude of slabs for routine diagnostics in this study was 100%, as clinically relevant areas were always present on either the front or back side of the slab of either the left or right hemisphere. Missing macroscopic lesions down to a diameter of 3–5 mm for post-mortem routine diagnostic workup is therefore very unlikely. However, without consideration of preliminary reports accompanying the submission form, brain pathologies with histological changes might be missed if the protocol is followed without actively seeking anticipated lesions. Hence, reasonable sampling under consideration of potentially affected brain regions, optimally accompanied by *in vivo* or *ex vivo* imaging studies (Stuckenschneider et al., 2014; Johnson et al., 2019; Schmidt et al., 2019), comprises the extension of standard sampling with additional homo- and heterotopic slabs. The diagnostic outcome of involved cases is beyond the scope of this study.

### Neuroanatomical Accuracy

Working down the checklist of selected anatomical hot spots, neuroanatomical accuracy was excellent in 88.3% of slabs and incomplete in only 4.8%, where <70% of landmarks were fully featured on the slide. As might be expected, experienced raters achieved the highest neuroanatomical scores with a median of 71.8 points (IQR: 68.8–72). However, following the protocol, people with no or limited experience still obtained excellent results with a median of 65.5 (IQR: 63.6–68.9) and 68 (IQR: 64.8–69.5), respectively. Therefore, the performance of beginners and those with some previous encounters only differed insignificantly.

### Influencing Factors

Among external factors, only pre-damage had a clear impact on neuroanatomical accuracy by obscuring external landmarks. Neither postmortem changes nor incomplete fixation diminished the neuroanatomical outcome in any of the groups, even though the candidates themselves felt subjectively uncomfortable in these situations, and histological preservation might be compromised.

### Fixation

To avoid the latter, penetration of fixative into tissue of these large animals may be accelerated (Furr and Reed, 2007a) by *ad hoc* dissection into the three blocks plus transverse section of the mid cerebellum (cut No. 1-TS−5-TS) in the necropsy hall or, even better, after superficial fixation for 24–48 h, to prevent cusping of gray matter at the cut surface. In fetuses and neonates with higher water content, brain tissue becomes easier to handle and cut when using zinc formalin for fixation (personal communication with Steffen Albrecht^2^) (Fortier and Hould, 2013).

### Heterogenity of Investigated Material

Coincidentally, the inexperienced group I dealt the most with brains with disruptive changes, so their anatomical performance may even be underestimated. Unfortunately, the limited number of donated brains precluded a systematic evaluation of damage scores equally distributed throughout the groups. In the same vein, the study mirrored the field situation regarding variability of brain sizes. For that reason, prescriptions of slice thickness must take into consideration the distances between landmarks. Hence, the thickness of obtained brain slabs ranged between 4 and 5 mm in foals to 10 mm in draft horses. An appropriate adaption with equidistant serial sections allows for proper presentation of all internal target zones as depicted in the brochure throughout the different sizes of the animals and, consequently, of their brains.

### Fixed Brain Weight/Dead Body Weight Ratio

As an interesting side finding, the relationship between body weight and brain weight was non-linear logarithmic relationship. Therefore, results support previous evaluations across species by confirming their hypothesis of brain-body weight interrelation for the first time within a cohort of equids (Jerison, 1973; Cozzi et al., 2014; Minervini et al., 2016).

### Institutional Implementation of the Protocol

Naturally, brain size has an effect on subsequent sampling for histology. Each institution will therefore trim the slabs of their regions of interest in accordance with their equipment with microtomes, cuvettes and glass slides with cover slips.

If an institution decides to implement the protocol shown herein, sampling of specific target areas for neuropathological or neuroscientific purposes can be performed easily and reproducibly, even if the person on the bench has no preexisting neuroanatomical knowledge.

The application of this protocol also allows for remote selection of regions of interest by external specialists that likewise are aware of the procedure, labeling and number of the slabs. Consequently, less experienced investigators can easily sample brain slabs triaged by experts and convey them for diagnostic and scientific purposes.

### Perspective and Forecast

As an incentive, the protocol is equally applicable for other polygyral mammals, such as bovids and new world camelids (data not shown). It is currently implemented in the Clinical & Comparative Neuropathology Laboratory, LMU Munich, for comparative lesion mapping in equine vs. ruminant vs. human brains.

Taken together, we strongly recommend researchers to take advantage of this practicable instruction for equine brain dissection in the field. However, it remains a task for future studies to define more accurately clear landmarks for angulation perpendicularity of planes for brain slabs and imaging slides and to create coregistered multimodal brain atlases in this species and to optimize imaging planes for measuring brain regions in correspondence to their specific histoarchitecture such as established for hippocampal scans in epileptic dogs and cats (Rusbridge et al., 2015).

## Data Availability Statement

The original contributions presented in the study are included in the article/Supplementary Material, further inquiries can be directed to the corresponding author.

## Ethics Statement

The animal study was reviewed and approved by Ethics Commission of the Centre for Veterinary Clinical Medicine of the LMU Munich. Written informed consent was obtained from the owners for the participation of their animals in this study.

## Author Contributions

KM and SR: conceptualization and design. LG, ZB, and KM: acquisition of material. M-LB, MR, ZB, KM, and SR: formal analysis, data acquisition and processing. SR: statistical analysis. ZB, LG, and KM: resources. M-LB: visualization. M-LB and KM: writing—original draft preparation. ZB, MR, LG, and SR: writing—review and editing. KM: supervision. All authors have read and agreed to the submitted version of the manuscript.

## Conflict of Interest

ZB was employed by the Austrian Agency for Health and Food Safety Ltd. The remaining authors declare that the research was conducted in the absence of any commercial or financial relationships that could be construed as a potential conflict of interest.

## Footnotes

^1^According to Oscar and Cécile Vogts' concept of selective vulnerability of different brain regions.

^2^Department of Pathology, The Montreal Childrens Hospital, McGill University, Montreal, Canada.

## Supplementary Material

High-resolution versions of the Supplementary Videos can be requested by email to the corresponding author: kaspar.matiasek@neuropathologie.de.
