# Supplementary material for: A Systematic Approach to Dissection of the Equine Brain–Evaluation of a Species-Adapted Protocol for Beginners and Experts
Source: Front Neuroanat. 2020 Dec 18;14:614929. doi: 10.3389/fnana.2020.614929 (PMC7775367; doi:10.3389/fnana.2020.614929)
Supplement: Supplementary file 1 [file Data_Sheet_1.pdf]

Supplementary Tables

Supplementary Table 1. Applied equipment for storage, measurement of brain weight, brain volume and time, as well as histoprocessing and final examination.

Application area	Denotation	Model/Manufacturer
Dead body weight (DBW) measurement	Scale	Panter BBA330-CC150, Mettler-Toledo GmbH, Albstadt, Germany
Brain storage	Containers with lid	EO 0351, Wolf Plastics Verpackungen GmbH, Kammern i. Liesingtal, Austria
Brain weight measurement	Scale	10.000 g x 1 g, Aevobas
Brain volume measurement	Measuring cup	3000 ml, 40814, Brand GmbH + Co. KG, Wertheim, Germany
Dissection	Customary cutting board	not specified
	Long knife (300 mm blade)	not specified
	Microtome blade	FEAT207500006_U, Feather Safety Razor Co. Ltd., Japan
	Scalpel handle	503, C. Bruno Bayha GmbH, Tuttlingen, Germany
	Scalpel blade	320, C. Bruno Bayha GmbH, Tuttlingen, Germany
	Microscope slide labeled with maximum slab size (herein 4 x 5.5 cm)	Large (03-0024, R. Langenbrinck GmbH, Emmerdingen, Germany)
Time measurement	Digital timer	38.2022, TFA Dostmann GmbH & Co. KG, Wertheim, Germany
Photo and video documentation	Camera	Samsung Galaxy S5 Duos G900FD
Graphical editing	Image processing software	paint.net<br>Windows Movie Maker
Postfixation	Metal tissue cassettes	Leica Biosystems, Nussloch, Germany
Tissue processing	Automatic tissue processor	14042280100 RevJ, Leica Biosystems, Nussloch, Germany
Paraffin embedding	Paraplast PLUS	39502004, Leica Biosystems, Nussloch, Germany
Cutting of FFPE	Rotary microtome	HM 315, Thermo Fisher Scientific, Waltham, MA, US
Stretching of cut tissue	Tissue float bath	1052, Gesellschaft für Labortechnik mbH, Burgwedel, Germany

Mounting	Microscope slides	Small (11250, Engelbrecht GmbH, Edermünde, Germany) Medium (03-0226, R. Langenbrinck GmbH, Emmerdingen, Germany)<br>Large (03-0024, R. Langenbrinck GmbH, Emmerdingen, Germany)
Coating of medium and large slides	Silanization	In-house production with A3648, Sigma-Aldrich Chemie GmbH, Munich, Germany
Embedding of stained tissue slides	Coverslips	e.g., K12440 (depending on matching size of microscope slides), Engelbrecht GmbH, Edermünde, Germany
Embedding of stained tissue slides	Histofluid mounting medium	19350, Engelbrecht GmbH, Edermünde, Germany
Microscopic examination	Zeiss Axioplan microscope	Carl Zeiss Microscopy, NY, United States

Supplementary Table 2. Formulations of deployed solutions for tissue fixation.

**Solution**	**Formulation**	**Manufacturer**
1 l phosphate-buffered formalin	6.5 g Di-sodium hydrogen phosphate	131679.1211, PanReac AppliChem, Darmstadt, Germany
	4.0 g Sodium dihydrogen phosphate monohydrate	131965.1211, PanReac AppliChem, Darmstadt, Germany
	100 ml Formaldehyde 37%	FO-10000-37-1, SAV LP GmbH, Flintsbach a. Inn, Germany
	900 ml H_2O dest	in-house production
1 l zinc formalin	3 g Zinc sulfate	8883, Merck KGaA, Darmstadt, Germany
	8 g Sodium chloride	131659.1211, PanReac AppliChem
	100 ml Formaldehyde 37%	FO-10000-37-1, SAV LP GmbH, Flintsbach a. Inn, Germany
	900 ml H_2O dest	in-house production
