# Supplementary material for: A Systematic Approach to Dissection of the Equine Brain–Evaluation of a Species-Adapted Protocol for Beginners and Experts
Source: Front Neuroanat. 2020 Dec 18;14:614929. doi: 10.3389/fnana.2020.614929 (PMC7775367; doi:10.3389/fnana.2020.614929)
Supplement: Supplementary file 2 [file Data_Sheet_2.pdf]

Supplementary Tables

Supplementary Table 3. Age, sex, breed and evidence of autolysis (AL) of each subject included in the study in chronological order [Age (d: days, w: weeks, m: months, y: years, -: premature/aborted); Sex (Fe: female entire, Mi: male intact (stallion), Mn: male neutered (gelding)); Breed (AH: arabian horse, DH: draft horse, OE: other equid, PO: pony, TB: thoroughbred, WB: warmblood); AL (0: fresh, 1: no macroscopic evidence of autolysis, 2: mild autolysis, 3: moderate autolysis)]

**Subject**	**Age**	**Sex**	**Breed**	**AL**
1	11y	Mi	Oldenburg horse (WB)	2
2	4y	Mn	Noriker horse (DH)	1
3	8w	Mi	Warmblood (WB)	1
4	4w	Fe	Icelandic horse (WB)	2
5	6y	Fe	Przewalski´s horse (OE)	1
6	19y	Mn	Pony (PO)	1
7	25y	Mn	Hanoverian horse (WB)	1
8	23y	Fe	Haflinger (PO)	2
9	12y	Mn	Dutch warmblood (WB)	0
10	11y	Mi	Friesian (WB)	2
11	5y	Mn	Shire Horse (DH)	0
12	12y	Mn	Warmblood (WB)	0
13	17y	Mn	Shetland pony (PO)	2
14	13y	Mn	Holsteiner horse (WB)	1
15	8y	Mn	German sport horse (WB)	2
16	9y	Fe	Warmblood (WB)	0
17	17y	Mn	Pony (PO)	2
18	4d	Mi	Warmblood (WB)	0
19	20y	Mn	Bavarian warmblood (WB)	0
20	Adult	Mn	Warmblood (WB)	0
21	3y	Mn	Warmblood (WB)	2
22	12.5y	Mn	Thoroughbred (TB)	0
23	23y	Mn	Zebra (OE)	3
24	13y	Fe	Warmblood (WB)	1
25	20.5y	Mn	German sport horse (WB)	2
26	Adult	Mn	Friesian (WB)	1
27	1.5y	Mi	Warmblood (WB)	1
28	6.5y	Mn	Westphalian horse (WB)	2
29	5d	Fe	Mini Shetland pony (PO)	1
30	7m	Fe	Trakehner (WB)	3
31	2d	Fe	Pura Raza Española (WB)	1
32	-4d	Mi	Warmblood (WB)	1
33	3y	Mn	Bavarian warmblood (WB)	0

34	1y	Mi	Trakehner (WB)	3
35	23y	Fe	German pony (PO)	2
36	1y	Fe	Wuerttemberg Warmblood (WB)	0
37	Adult	Fe	Warmblood (WB)	1
38	16y	Mn	Shetland pony (PO)	3
39	5y	Mn	Pura Raza Española (WB)	0
40	1.3y	Fe	Arabian horse (AH)	1
41	Adult	Mn	Warmblood (WB)	0
42	15y	Mn	Haflinger (PO)	2
43	8y	Mi	Wuerttemberg Warmblood (WB)	0
44	Adult	Mi	Haflinger (PO)	1
45	11y	Fe	Warmblood (WB)	0
46	25y	Mn	Arabian horse (AH)	1
47	-8w	Fe	Warmblood (WB)	2
48	-8w	Fe	Iberian horse (WB)	2
49	-4w	Mi	Thoroughbred (TB)	1
50	10y	Fe	Warmblood (WB)	1
51	7y	Mn	Warmblood (WB)	1
52	-4w	Mi	Trakehner (WB)	2
53	1.5d	Mi	Hanoverian horse (WB)	1
54	Adult	Fe	Warmblood (WB)	0
55	Adult	Mn	Thoroughbred (TB)	0

Supplementary Table 4. Demographics of 22 subjects: dead body weight (DBW) in kilograms (kg), brain weight (BW) post fixation in grams (g) and brain volume post fixation in cubic centimeter (cm^3)

**Subject**	**DBW (kg)**	**BW (g)**	**BV (cm^3)**
4	49.6	362	405
5	350	589	660
9	500	670	750
10	450	666	745
13	135	353	395
20	700	674	755
29	10.8	312	350
31	65	272	305
32	48	352	395
33	600	669	750
35	200	660	740
37	350	665	745
40	200	361	405
41	550	576	645
42	350	540	605
43	500	621	695
44	600	664	745

47	18.5	353	395
48	25	357	400
49	28	362	405
51	600	710	795
52	30.7	223	250

Supplementary Table 5. Total procedure times (TPT) in minutes throughout trials (T1 to T5) and groups (I to III). SD: standard deviation; IQR: interquartile range

Rater	Group	TPT (T1)	TPT (T2)	TPT (T3)	TPT (T4)	TPT (T5)	Mean±SD	Median (IQR)
1	I	55	40	28	24	18	33±14.7	28(21 to 47.5)
2	I	52	45	36	28	21	36.4±12.5	36(24.5 to 48.5)
3	I	55	37	37	21	19	33.8±14.6	37(20 to 46)
4	I	54	50	66	40	35	49±12.2	50(37.5 to 60)
5	II	40	38	41	25	18	32.4±10.3	38(21.5 to 40.5)
6	II	55	42	35	30	25	40.4±9.4	40(32.5 to 48.5)
7	II	40	32	28	21	18	27.8±8.8	28(19.5 to 36)
8	II	38	35	30	20	15	27.6±9.8	30(17.5 to 36.5)
9	II	32	28	30	17	15	24.4±7.8	28(16 to 31)
10	III	35	30	25	15	14	23.8±9.2	25(14.5 to 32.5)
11	III	40	35	25	20	18	27.6±9.6	25(19 to 37.5)

Supplementary Table 6. Scores throughout trials (T1 to T5) and groups (I to III). SD: standard deviation; IQR: interquartile range; CV: coefficient of variation

Rater	Group	Score (T1)	Score (T2)	Score (T3)	Score (T4)	Score (T5)	Mean ±SD	Median (IQR)	CV
1	I	60	69	61	71.5	72	66.7±5.8	69(60.5 to 71.7)	8.7%
2	I	65	70.5	68	62	64.5	66±3.3	65(63.3 to 69.3)	5.0%
3	I	64	68	64	63.5	68.5	65.5±2.4	64(63.8 to 68.3)	4.6%
4	I	65.5	71	63	65.5	68.5	66.7±3.1	65.5(64.3 to 69.8)	3.7%
5	II	64	65	64.5	71	69	66.7±3.1	65(64.3 to 70)	5.9%
6	II	65.5	69.5	60.5	63.5	69.5	65.7±3.9	65.5(62 to 69.5)	4.7%
7	II	68	64.5	66.5	66	69	66.8±1.7	66.5(65.3 to 68.5)	3.0%
8	II	64	68	68.5	68	69	67.5±2	68(66 to 68.8)	2.6%
9	II	72	70.5	67.5	70	69.5	69.9±1.6	70(68.5 to 71.3)	2.3%
10	III	71.5	71.5	66.5	69.5	63.5	68.5±3.5	69.5(65 to 71.5)	5.1%
11	III	72	72	72	72	72	72±0	72(72 to 72)	0.0%

Supplementary Table 7. Details regarding aptitude of slabs for neuroanatomical studies by reference to 36 criteria (see Table 1) across all groups. Strikes of anatomical landmarks were scored as either full match (1), partly featured (0.5) or not evident at all (0), and slabs were graded as excellent (> 80% of landmarks fully featured on the slide), sufficient (70-80% of landmarks fully featured on the slide) or insufficient (< 70% of landmarks fully featured on the slide) for neuroanatomical studies

**Slab**		**Aptitude for neuroanatomical studies**			**Total**
		excellent	sufficient	insufficient	
1	Total № of slabs	47	5	3	55
	%	85.5%	9.1%	5.5%	100.0%
2	Total № of slabs	42	7	6	55
	%	76.4%	12.7%	10.9%	100.0%
3	Total № of slabs	50	3	2	55
	%	90.9%	5.5%	3.6%	100.0%
4	Total № of slabs	55	0	0	55
	%	100.0%	0.0%	0.0%	100.0%
5	Total № of slabs	55	0	0	55
	%	100.0%	0.0%	0.0%	100.0%
6	Total № of slabs	50	1	4	55
	%	90.9%	1.8%	7.3%	100.0%
7	Total № of slabs	46	4	5	55
	%	83.6%	7.3%	9.1%	100.0%
8	Total № of slabs	44	2	9	55
	%	80.0%	3.6%	16.4%	100.0%
9	Total № of slabs	50	5	0	55
	%	90.9%	9.1%	0.0%	100.0%
10	Total № of slabs	51	4	0	55
	%	92.7%	7.3%	0.0%	100.0%
11	Total № of slabs	53	1	1	55
	%	96.4%	1.8%	1.8%	100.0%
12	Total № of slabs	44	8	3	55
	%	80.0%	14.5%	5.5%	100.0%
13	Total № of slabs	43	8	4	55
	%	78.2%	14.5%	7.3%	100.0%
14	Total № of slabs	50	5	0	55
	%	90.9%	9.1%	0.0%	100.0%
Total	Total № of slabs	680	53	37	770

	%	88.3%	6.9%	4.8%	100.0%

Supplementary Table 8. Comparison of absolute (A) and relative (R) scores and coefficient of variation (CV) between all slabs and groups. Most problematic slabs per group with a mean relative score < 90% are highlighted in dark gray

**Slab**	**Score group I**			**Score group II**			**Score group III**		
	A		R (%)	A		R (%)	A		R (%)
	Range	Mean±SD	Mean±SD	Range	Mean±SD	Mean±SD	Range	Mean±SD	Mean±SD
1	3-10	8.5±1.9	85±18.7	6-10	8.8±1.0	88.4±10.3	8.5-10	9.6±0.7	96±6.6
2	5-8	7.3±1.1	90.8±13.2	4-8	6.9±1.2	86.1±14.9	8- 8	8.0±0.0	100±0
3	7.5-10	9.7±0.9	96.5±8.6	5-10	9.3±1.5	93.2±15.2	10- 10	10.0±0.0	100±0
4	2-2	2.0±0.0	100±0	2-2	2.0±0.0	100±0	2-2	2.0±0.0	100±0
5	4-4	4.0±0.0	100±0	4-4	4.0±0.0	100±0	4-4	4.0±0.0	100±0
6	2-4	3.7±0.6	93.2±15.4	2-4	3.9±0.4	98±10	0-4	3.5±1.6	86.3±31.4
7	1-4	3.6±0.8	88.9±19.8	1-4	3.6±0.8	89.6±18.9	4-4	4.0±0.0	100±0
8	4-6	5.3±0.8	88.4±18.8	4-6	5.4±0.8	90.3±12.6	3-6	5.7±.0.9	94.2±15.7
9	3-4	3.8±0.4	95.7±9.3	3-4	3.9±0.3	98±6.9	4-4	4.0±0.0	100±0
10	6-8	7.6±0.8	95±9.7	6-8	7.8±0.5	97.6±6.2	7-8	7.9±0.3	98.8±3.8
11	2-2	2.0±0.0	100±0	1.5-2	2.0±0.1	99±5	1-2	1.9±0.3	95±15.8
12	0-2	1.8±0.5	87.5±23.6	0-2	1.9±0.4	93±22.3	1.5-2	2.0±0.2	97.5±7.9
13	0-4	3.3±1.3	81.3±32.3	3-4	3.8±0.4	96±9.4	3-4	3.9±0.3	97.5±7.9
14	3-4	3.9±0.4	96.3±9.2	3-4	4.0±0.2	99±5	3-4	3.9±0.3	97.5±7.9

Supplementary Table 9. Details regarding symmetry of slabs across all groups. Strikes of symmetry were scored as either good (1), moderate (0.5) or not present (0).

**Slab**		**Symmetry**			**Total**
		Good	Moderate	Not present	
1	Total № of slabs	39	16	0	55
	%	70.9%	29.1%	0.0%	100.0%
2	Total № of slabs	37	18	0	55
	%	67.3%	32.7%	0.0%	100.0%
3	Total № of slabs	49	6	0	55
	%	89.1%	10.9%	0.0%	100.0%
4	Total № of slabs	45	10	0	55
	%	81.8%	18.2%	0.0%	100.0%
5	Total № of slabs	43	12	0	55
	%	78.2%	21.8%	0.0%	100.0%
6	Total № of slabs	46	8	1	55
	%	83.6%	14.5%	1.8%	100.0%
7	Total № of slabs	33	22	0	55
	%	60.0%	40.0%	0.0%	100.0%
8	Total № of slabs	39	16	0	55
	%	70.9%	29.1%	0.0%	100.0%
9	Total № of slabs	48	7	0	55
	%	87.3%	12.7%	0.0%	100.0%
10	Total № of slabs	49	6	0	55
	%	89.1%	10.9%	0.0%	100.0%
11	Total № of slabs	17	5	0	22
	%	77.3	22.7%	0.0%	100.0%
12	Total № of slabs	47	6	2	55
	%	85,5%	10,9%	3,6%	100,0%
13	Total № of slabs	43	10	2	55
	%	78,2%	18,2%	3.6%	100,0%
14	Total № of slabs	17	5	0	22
	%	77,3%	22,7%	0.0%	100,0%
Total	Total № of slabs	552	147	5	704
	%	78.4%	20.9%	0.7%	100.0%

Supplementary Table 10. Symmetry comparison of absolute (A) and relative (R) numbers of slabs throughout groups I to III. Slab 11 and 14 could only be assessed for symmetry in hemispheric approaches. Most problematic slabs per group with an amount of < 70% of slabs with good symmetry are highlighted in dark gray

**Slab**	**Symmetry group I**						**Symmetry group II**						**Symmetry group III**					
	Good		Moderate		Not present		Good		Moderate		Not present		Good		Moderate		Not present	
	A	R (%)	A	R (%)	A	R (%)	A	R (%)	A	R (%)	A	R (%)	A	R (%)	A	R (%)	A	R (%)
1	14	70	6	30	0	0	17	68	8	32	0	0	8	80	2	20	0	0
2	14	70	6	30	0	0	15	60	10	40	0	0	8	80	2	20	0	0
3	18	90	2	10	0	0	21	84	4	16	0	0	10	100	0	0	0	0
4	13	65	7	35	0	0	24	96	1	4	0	0	8	80	2	20	0	0
5	16	80	4	20	0	0	20	80	5	20	0	0	7	70	3	30	0	0
6	14	70	6	30	0	0	25	100	0	0	0	0	7	70	2	20	1	10
7	13	65	7	35	0	0	15	60	10	40	0	0	5	50	5	50	0	0
8	12	60	8	40	0	0	19	76	6	24	0	0	8	80	2	20	0	0
9	17	85	3	15	0	0	21	84	4	16	0	0	10	100	0	0	0	0
10	17	85	3	15	0	0	22	88	3	12	0	0	10	100	0	0	0	0
11	6	75	2	25	0	0	8	80	2	20	0	0	3	75	1	25	0	0
12	14	70	5	25	1	5	23	92	1	4	1	4	10	100	0	0	0	0
13	15	75	3	15	2	10	20	80	5	20	0	0	8	80	2	20	0	0
14	7	87.5	1	12.5	0	0	7	70	3	30	0	0	3	75	1	25	0	0

Supplementary Table 11. Details regarding angulation error of slabs from prescribed cutting angle across all groups. Strikes of deviation were scored as either correct angle (1), mild deviation (0.5) or severe deviation (0), and slabs were graded as excellent (≥ 80% of slabs with correct angle) sufficient (70-80% of slabs with correct angle) or insufficient (< 70% of slabs with correct angle)

Slab		Angulation error			Total
		Correct angle	Mild deviation	Severe deviation	
1	Total № of slabs	42	5	8	55
	%	76.4%	9.1%	14.5%	100.0%
2	Total № of slabs	33	0	22	55
	%	60.0%	0.0%	40.0%	100.0%
3	Total № of slabs	47	0	8	55
	%	85.5%	0.0%	14.5%	100.0%
4	Total № of slabs	48	7	0	55
	%	87.3%	12.7%	0.0%	100.0%
5	Total № of slabs	55	0	0	55
	%	100.0%	0.0%	0.0%	100.0%
6	Total № of slabs	46	2	7	55
	%	83.6%	3.6%	12.7%	100.0%
7	Total № of slabs	43	6	6	55
	%	78.2%	10.9%	10.9%	100.0%
8	Total № of slabs	31	14	10	55
	%	56.4%	25.5%	18.2%	100.0%
9	Total № of slabs	46	8	1	55
	%	83.6%	14.5%	1.8%	100.0%
10	Total № of slabs	46	5	4	55
	%	83.6%	9.1%	7.3%	100.0%
11	Total № of slabs	48	6	1	55
	%	87.3%	10.9%	1.8%	100.0%
12	Total № of slabs	49	4	2	55
	%	89.1%	7.3%	3.6%	100.0%
13	Total № of slabs	51	4	0	55
	%	92.7%	7.3%	0.0%	100.0%
14	Total № of slabs	48	5	2	55
	%	87.3%	9.1%	3.6%	100.0%
Total	Total № of slabs	633	66	71	770

	%	82.2%	8.6%	9.2%	100.0%

Supplementary Table 12. Grading of absolute (A) and relative (R) numbers of slabs concerning angulation errors throughout groups and approaches. Most problematic slabs per group with an amount of < 70% of slabs with correct angle are highlighted in dark gray

**Slab**	**Angulation error group I**						**Angulation error group II**						**Angulation error group III**					
	Correct angle		Mild deviation		Severe deviation		Correct angle		Mild deviation		Severe deviation		Correct angle		Mild deviation		Severe deviation	
	A	R (%)	A	R (%)	A	R (%)	A	R (%)	A	R (%)	A	R (%)	A	R (%)	A	R (%)	A	R (%)
1	15	75	1	5	4	20	18	72	4	16	3	12	9	90	0	0	1	10
2	14	70	0	0	6	30	13	52	0	0	12	48	6	60	0	0	4	40
3	18	90	0	0	2	10	20	80	0	0	5	20	9	90	0	0	1	10
4	14	70	0	0	6	30	25	100	0	0	0	0	9	90	1	10	0	0
5	20	100	0	0	0	0	25	100	0	0	0	0	10	100	0	0	0	0
6	14	70	2	10	4	20	25	100	0	0	0	0	7	70	0	0	3	30
7	12	60	4	20	4	20	21	84	2	8	2	8	10	100	0	0	0	0
8	10	50	5	25	5	25	13	52	8	32	4	16	8	80	1	10	1	10
9	16	80	4	20	0	0	21	84	3	12	1	4	9	90	1	10	0	0
10	16	80	1	5	3	15	21	84	3	12	1	4	9	90	1	10	0	0
11	18	90	2	10	0	0	20	80	4	16	1	4	10	100	0	0	0	0
12	15	75	4	20	1	5	24	96	0	0	1	4	10	100	0	0	0	0
13	18	90	2	10	0	0	23	92	0	0	2	8	10	100	0	0	0	0
14	18	90	2	10	0	0	22	88	1	4	2	8	8	80	2	20	0	0

Supplementar Table 13. Correlation between grade of damage and identifiability of landmarks in absolute (A) and relative (R) numbers of slabs throughout groups and approaches. Grades of damage (1: no macroscopic alterations; 2: negligible alterations; 3: moderate alterations; 4: severe alterations)

**Grade of damage**	**Identifiability group I**						**Identifiability group II**						**Identifiability group III**	
	Easy		Fair		Moderate		Easy		Fair		Moderate		Easy	
	A	R (%)	A	R (%)	A	R (%)	A	R (%)	A	R (%)	A	R (%)	A	R (%)
1	104	70.3	80	71.4	3	15	191	82	75	72.8	6	42.9	113	80.7
2	23	15.5	9	8.0	0	0	6	2.6	6	5.8	0	0	12	8.6
3	18	12.2	5	4.5	0	0	21	9	14	13.6	2	14.3	6	4.3
4	3	2.0	18	16.1	17	85	15	6.4	8	7.8	6	42.9	9	6.4
