# Supplementary material for: A Systematic Approach to Dissection of the Equine Brain–Evaluation of a Species-Adapted Protocol for Beginners and Experts
Source: Front Neuroanat. 2020 Dec 18;14:614929. doi: 10.3389/fnana.2020.614929 (PMC7775367; doi:10.3389/fnana.2020.614929)
Supplement: Supplementary file 5 [file Data_Sheet_5.PDF]

Bihemispheric version

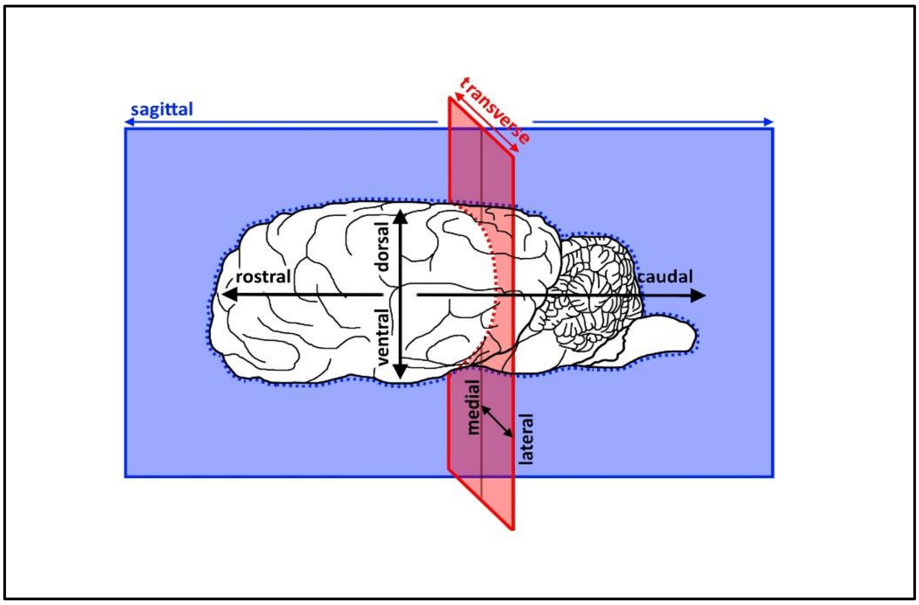

Supplementary Figure 1. Planes and orientation. Colours demonstrate sagittal (blue) and transverse (red) planes. The sections performed in the protocol are numbered from 1-21 in the order of implementation and termed according to the individual plane (e.g., 1-TS, 6-SAG, 8-TILT). Sagittal (SAG), lateral-tilted (TILT), transverse (TS) and transverse-tilted (TS-TILT).

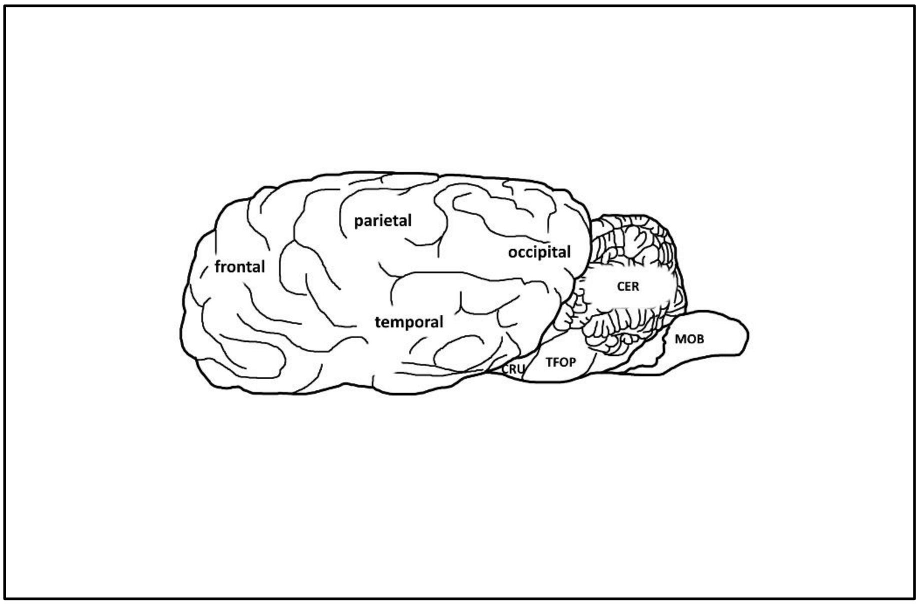

Supplementary Figure 2. Landmarks of the dorsolateral brain surface. Forebrain with frontal, parietal, temporal, occipital lobe. Midbrain with cerebral crus (CRU). Hindbrain with transverse fibres of pons (TFOP), cerebellum (CER) and medulla oblongata (MOB).

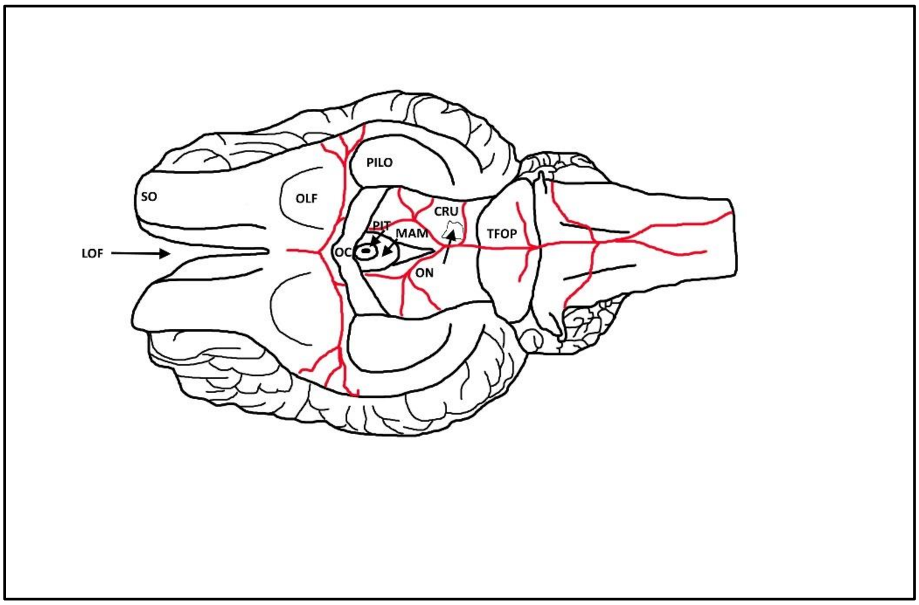

Supplementary Figure 3. Landmarks of the ventral brain surface. Cerebral crus (CRU), longitudinal fissure (LOF), mammillary bodies (MAM), optic chiasm (OC), olfactory tubercle (OLF), oculomotor nerve (ON), piriform lobe (PILO), pituitary stalk/infundibular recess (PIT), stria olfactoria (SO) and transverse fibres of pons (TFOP).

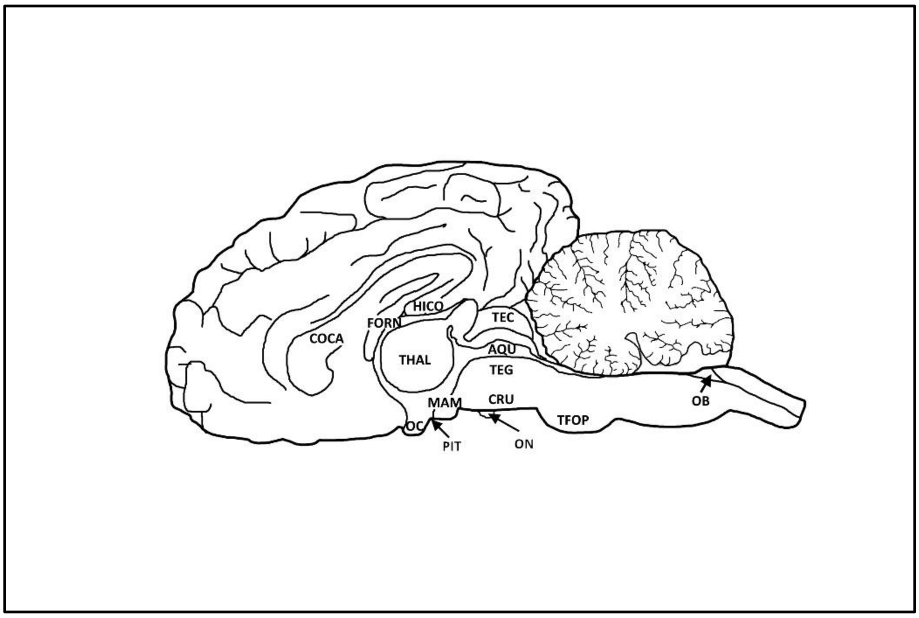

Supplementary Figure 4. Landmarks of the median brain surface. Mesencephalic aqueduct (AQU), corpus callosum (COCA), cerebral crus (CRU), fornix (FORN), hippocampal commissure (HICO), mammillary bodies (MAM), obex (OB), optic chiasm (OC), oculomotor nerve (ON), pituitary stalk/ infundibular recess (PIT), midbrain tectum (TEC), midbrain tegmentum (TEG), transverse fibres of pons (TFOP) and thalamus (THAL).

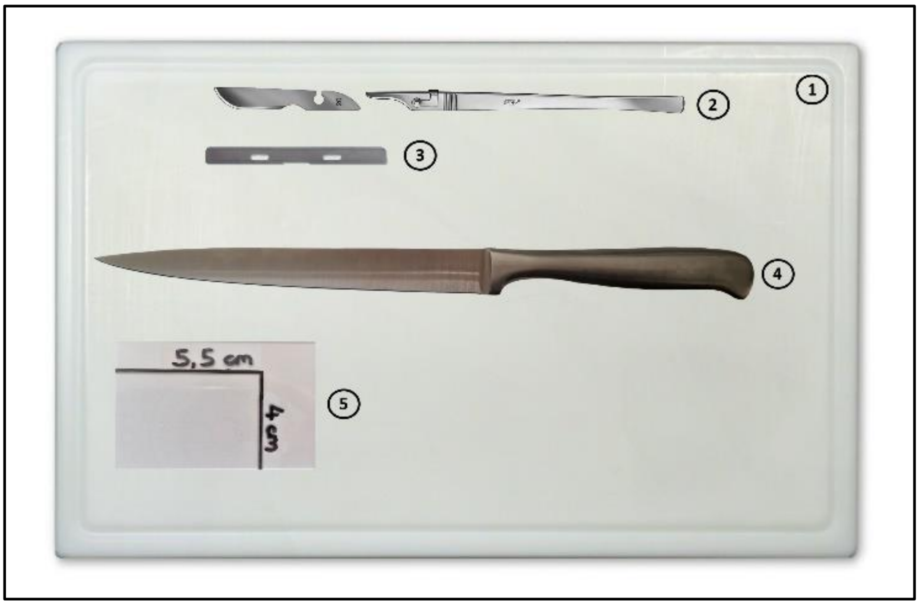

Supplementary Figure 5. Equipment for performance of the protocol. Cutting board (1), scalpel blade and handle (2), microtome blade (3), customary long blade (4), microscope slide labelled with maximum slab size herein 4 x 5.5cm (5)

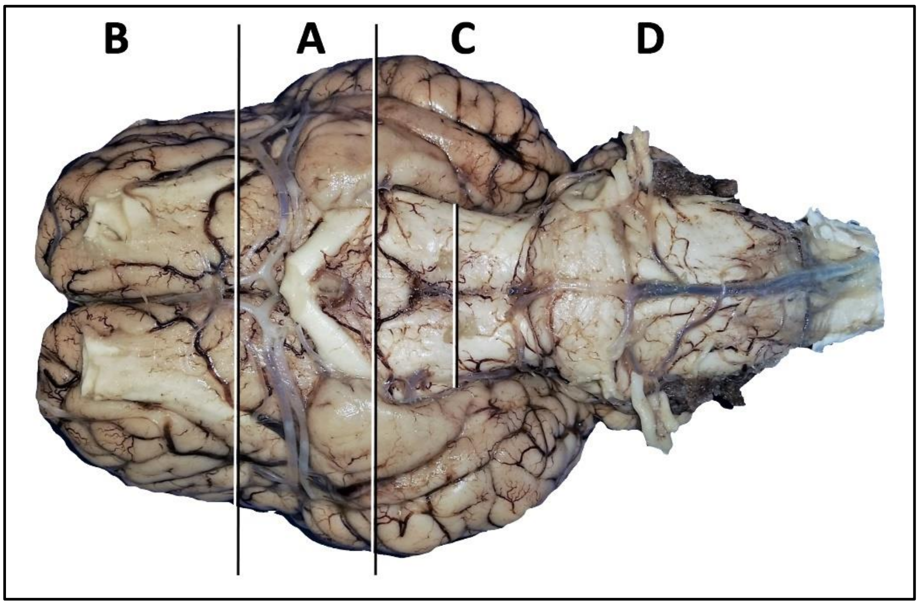

Supplementary Figure 6. Virtual division of the brain into 4 blocks (A to D) for simplification of tissue handling and obtainment of even cut surfaces for placement of the blocks on the worktop.

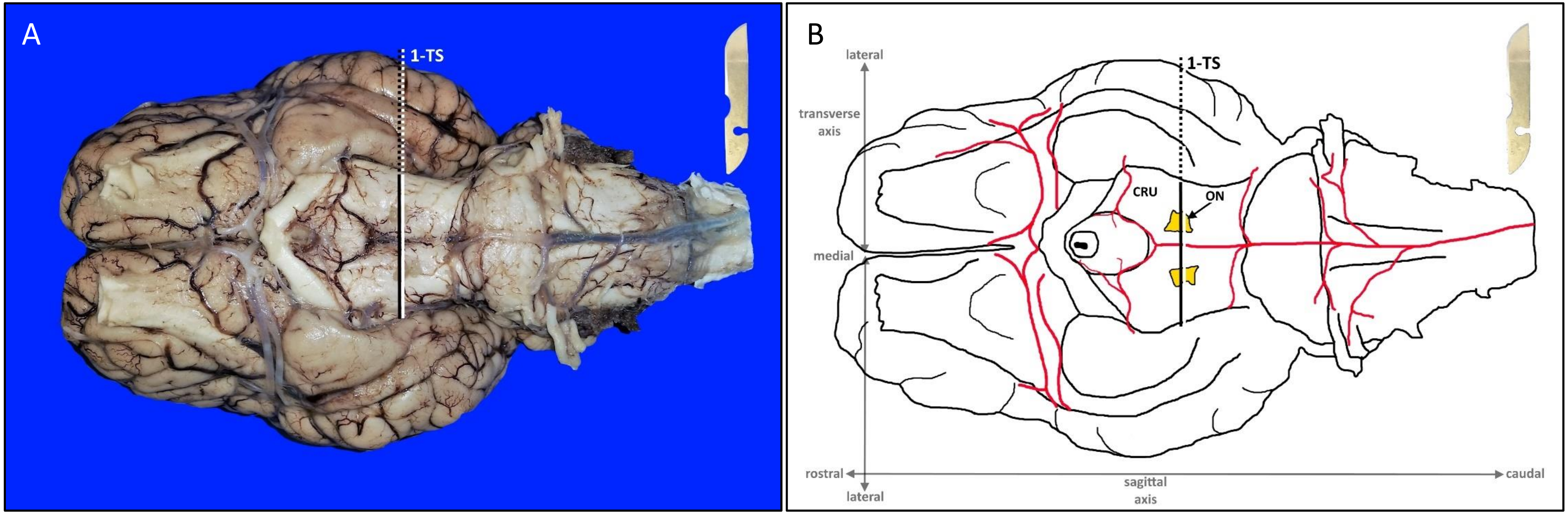

Supplementary Figure 7. Landmarks for 1-TS: cerebral crus (CRU), oculomotor nerve (ON). Transverse section (TS). Blood vessels (red), pertinent landmark (yellow). Scalpel blade. (A) Fixed brain. (B) Schematic illustration.

Supplementary Table 1 Instruction for performance of 1-TS (Supplementary Figure 7)

Step	Aim / harvest	Placement and preparation	View / specimen	Landmarks and cutting levels	Orientation of sections	Difficulty
**1-TS**	Transsection of the midbrain<br><br>Cave: do not cut into occipital lobe (use small blade)	Place the brain with its dorsal surface on the worktop and fix it between your thumb and forefinger to allow for ventrodorsal section	Ventral top view of the brain	Transverse line through cerebral crus at oculomotor nerve emergence	*2D knife axis:* laterolateral<br><br>*Plane:* transverse (TS)<br><br>*Inclination:* orthogonal to worktop<br><br>*Blade movement:* ventrodorsal	Easy

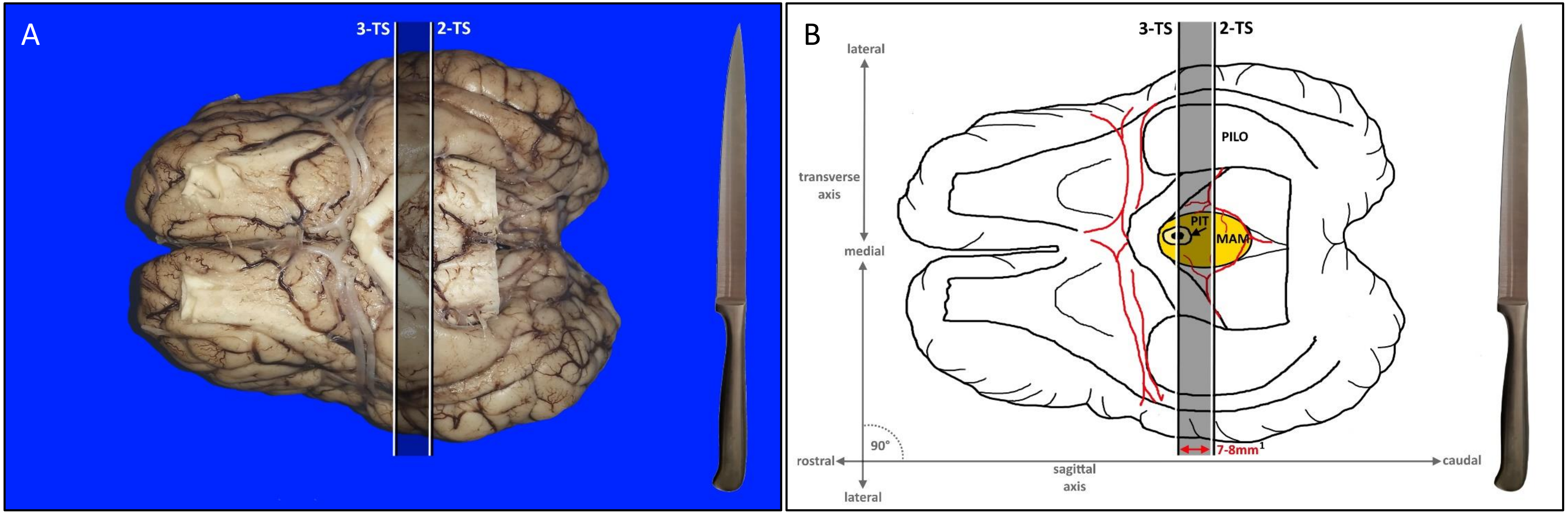

Supplementary Figure 8. Landmarks for 2-TS and 3-TS: mammillary bodies (MAM), piriform lobe (PILO), pituitary stalk/ infundibular recess (PIT). Transverse section (TS). Transverse section (TS). Blood vessels (red), pertinent landmark (yellow), requested slab (graying). Customary long blade. (A) Fixed brain. (B) Schematic illustration.

Supplementary Table 2 Instructions for obtainment of 1st slab out of Block A (Supplementary Figure 8)

**Step**	**Positioning and Preparation**	**View / specimen**	**Landmarks and cutting levels**	**Orientation of sections**	**Exposed structures**	**Difficulty**
**2-TS**	Place the block with its dorsal surface on the worktop and fix it between your thumb and forefinger to allow for ventrodorsal section.<br><br>→ hold brain in the way and direction of your convencience	Ventral top view of the brain	Transverse line through widest part of piriform lobe and mammillary bodies	*2D knife axis:* laterolateral<br><br>*Plane:* transverse (TS)<br><br>*Inclination:* orthogonal to worktop<br><br>*Blade movement:* ventrodorsal	• hippocampal commissure<br>• temporoventral body of hippocampus<br>• amygdaloid nucleus<br>• optic tract<br>• cerebral crus<br>• mammillary bodies<br>• piriform cortex	Easy

**3-TS**	Maintain placement as stated above	as above	Transverse line through pituitary stalk/infundibular recess<br><br>resp. 7-8mm[1] parallel and rostral to 2-TS	as above	• prehippocampal fornix<br>• caudate nucleus (tail)<br>• ventral anterior nucleus<br>• optic tract<br>• periventricular zone of the hypothalamus<br>• piriform cortex	Easy

[1] the distance (mm) between sections depends on animal size resp. brain size and ranges from 4-5mm (foal) to 10mm (draft horse)

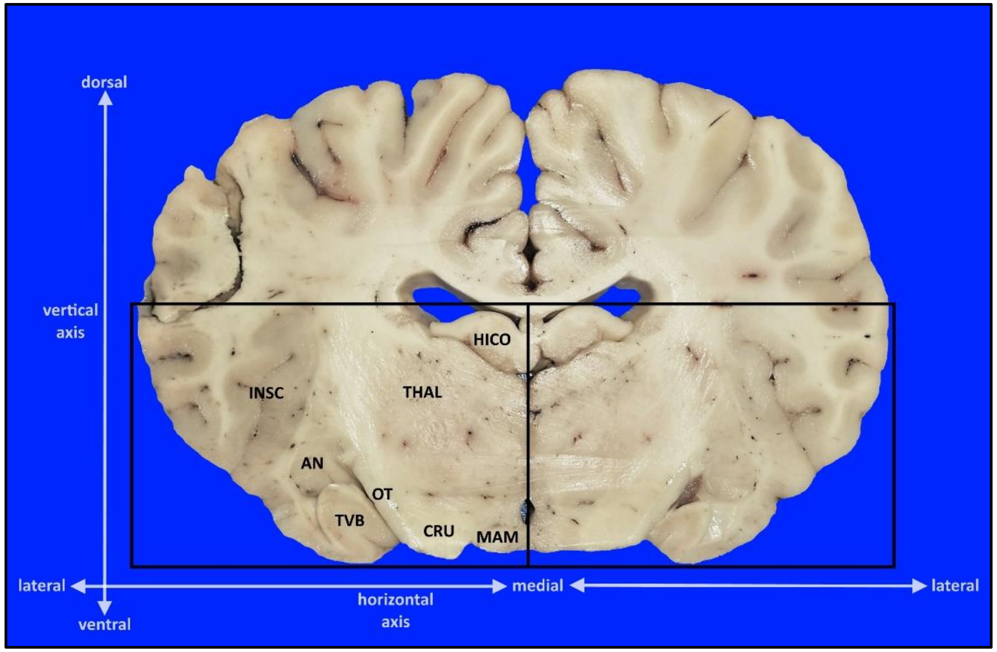

Supplementary Figure 8.1. Top view. 1st slab out of Block A. Proposed area for histoprocessing (black frame). Pertinent landmarks for sampling: amygdaloid nucleus (AN), cerebral crus (CRU), hippocampal commissure (HICO), insular cortex (INSC), mammillary bodies (MAM), optic tract (OT), thalamus (THAL), temporoventral body of hippocampus (TVB).

Supplementary Table 2.1 Trimming of 1st slab according to sampling recommendations

Placement of the slab	View / specimen	Landmarks and cutting levels	Target structures	Slab size	Difficulty
Rostral cutting surface[2]	Top view	Horizontal line above hippocampal commissure<br><br>Vertical line at midline for separation of hemispheres	• hippocampal commissure<br>• thalamus<br>• temporoventral body of hippocampus<br>• amygdaloid nucleus<br>• optic tract<br>• cerebral crus<br>• mammillary bodies<br>• piriform cortex	maximum 4cm x 5.5cm (herein)	Easy

[2] use the side/surface better exposing the target structures resp. the lesion

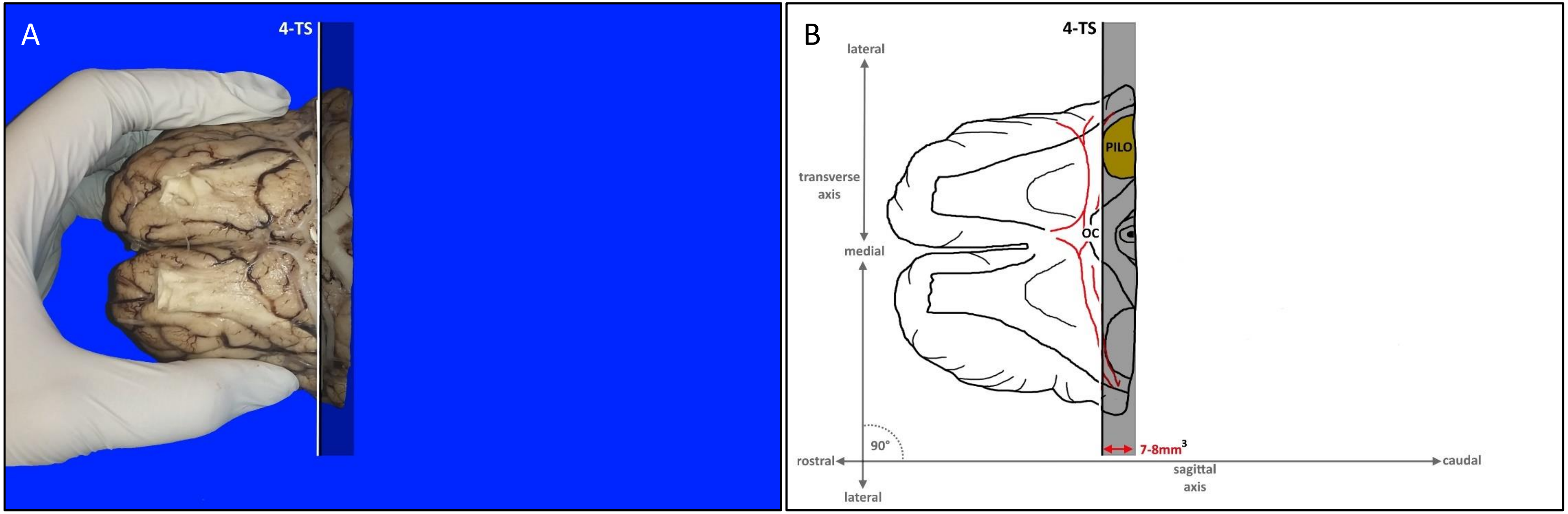

Supplementary Figure 9. Landmarks for 4-TS: optic chiasm (OC), piriform lobe (PILO). Transverse section (TS). Blood vessels (red), pertinent landmark (yellow), requested slab (graying). (A) Fixed brain. (B) Schematic illustration.

Supplementary Table 3 Instruction for obtainment of 2nd slab out of Block A (Supplementary Figure 9)

Step	Positioning and Preparation	View / specimen	Landmarks and cutting levels	Orientation of sections	Exposed structures	Difficulty
**4-TS**	Maintain placement as stated above	as above	Transverse line at the caudal border of the optic chiasm, rostral to piriform lobe<br><br>resp. 7-8mm[3] parallel and rostral to 3-TS	*2D knife axis:* laterolateral<br><br>*Plane:* transverse (TS)<br><br>*Inclination:* orthogonal to worktop<br><br>*Blade movement:* ventrodorsal	• prehippocampal fornix<br>• basal nuclei<br>• capsules<br>• rostral commissure<br>• septal nuclei<br>• prepiriform cortex	Easy

[3] the distance (mm) between sections depends on animal size resp. brain size and ranges from 4-5mm (foal) to 10mm (draft horse)

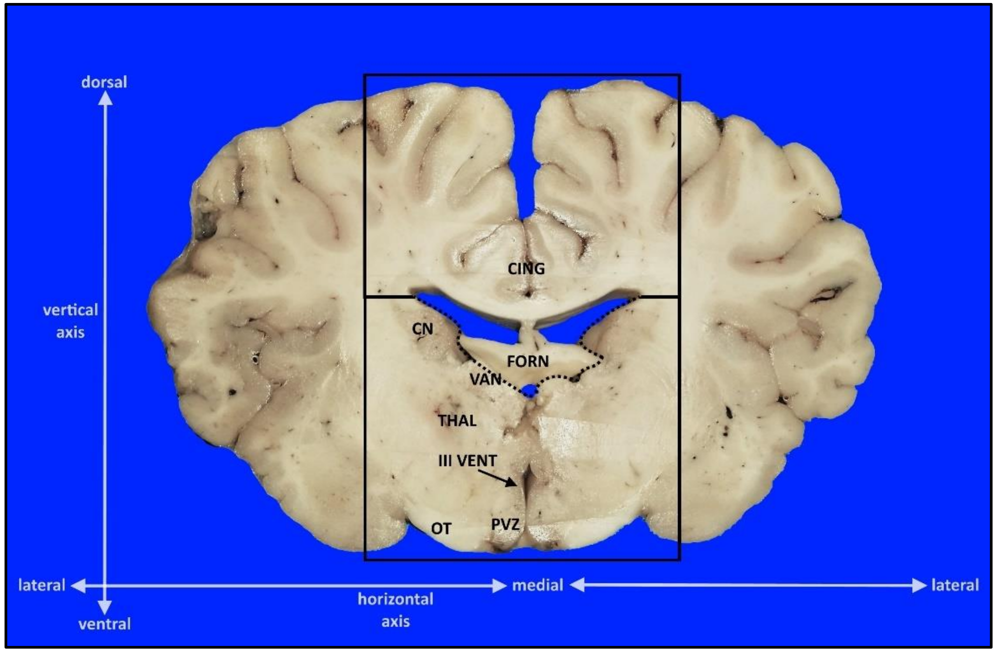

Figure 9.1. Top view. 2nd slab out of Block A. Proposed area for histoprocessing (black frame). Pertinent landmarks for sampling: 3rd ventricle (III VENT), cingulate gyrus (CING), tail of caudate nucleus (CN), fornix (FORN), optic tract (OT), periventricular zone (PVZ) of hypothalamus, thalamus (THAL), ventral anterior nucleus (VAN).

Supplementary Table 3.1 Trimming of 2nd slab according to sampling recommendations

Placement of the slab	View / specimen	Landmarks and cutting levels	Target structures	Slab size	Difficulty
Rostral cutting surface[4]	Top view	Horizontal line at lateral angle of lateral ventricles<br><br>Vertical line to adjust to maximum slab size	• corpus callosum<br>• ventral anterior nucleus<br>• caudate nucleus (tail)<br>• optic tract<br>• periventricular zone of hypothalamus	maximum 4cm x 5.5cm (herein)	Easy

[4] use the side/surface better exposing the target structures resp. the lesion

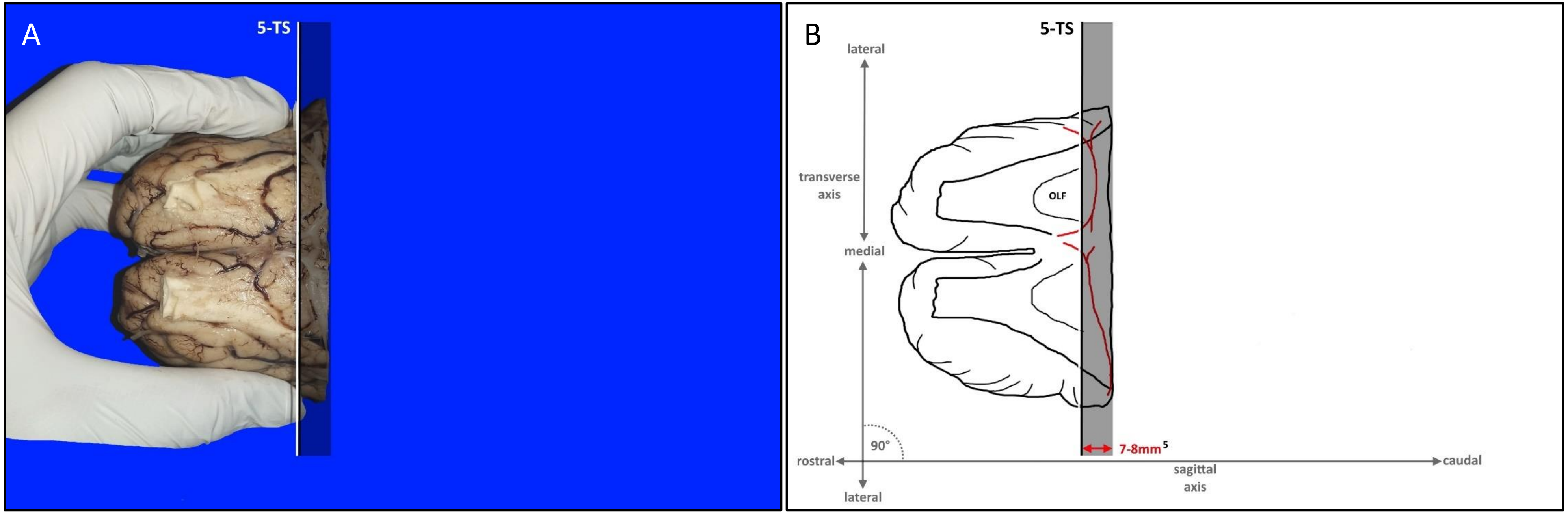

Supplementary Figure 10. Landmarks for 5-TS: olfactory tubercle (OLF). Transverse section (TS). Blood vessels (red), pertinent landmark (yellow), requested slab (graying). (A) Fixed brain. (B) Schematic illustration.

Supplementary Table 4 Instruction for obtainment of 3rd slab out of Block A (Supplementary Figure 10)

Step	Positioning and Preparation	View / specimen	Landmarks and cutting levels	Orientation of sections	Exposed structures	Difficulty
**5-TS**	Maintain placement as stated above	as above	Transverse line through pre-piriform cortex and olfactory tubercle rostral to optic chiasm<br><br>resp. 7-8mm[5] parallel and rostral to 4-TS	*2D knife axis:* laterolateral<br><br>*Plane:* transverse (TS)<br><br>*Inclination:* orthogonal to worktop<br><br>*Blade movement:* ventrodorsal	• prehippocampal fornix<br>• basal nuclei<br>• capsules<br>• rostral commissure<br>• septal nuclei<br>• frontotemporal cortex	Easy

[5] the distance (mm) between sections depends on animal size resp. brain size and ranges from 4-5mm (foal) to 10mm (draft horse)

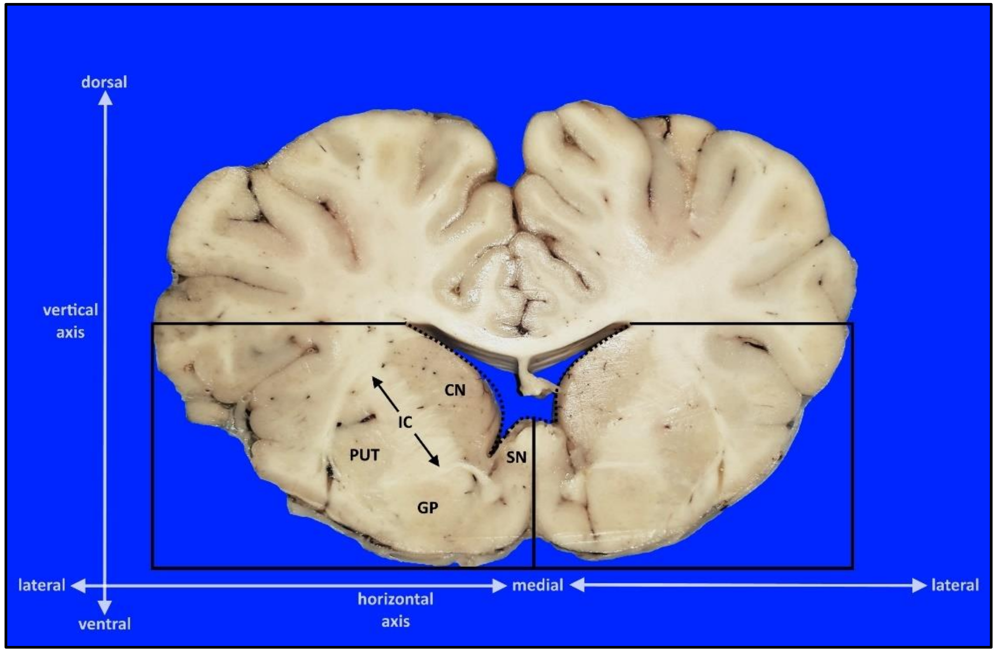

Supplementary Figure 10.1. Top view. 3rd slab out of Block A. Proposed area for histoprocessing (black frame). Pertinent landmarks for sampling: caudate nucleus (CN), globus pallidus (GP), internal capsule (IC), putamen (PUT), septal nuclei (SN).

Table 4.1 Trimming of 3rd slab according to sampling recommendations

**Placement of the slab**	**View / specimen**	**Landmarks and cutting levels**	**Target structures**	**Slab size**	**Difficulty**
Caudal cutting surface[6]	Top view	Horizontal line at lateral angle of lateral ventricles<br><br>Vertical line at midline for separation of hemispheres to adjust to maximum slab size	• caudate nucleus<br>• putamen<br>• globus pallidus<br>• internal capsule<br>• septal nuclei<br>• frontotemporal cortex	maximum 4cm x 5.5cm (herein)	Easy

[6] use the side/surface better exposing the target structures resp. the lesion

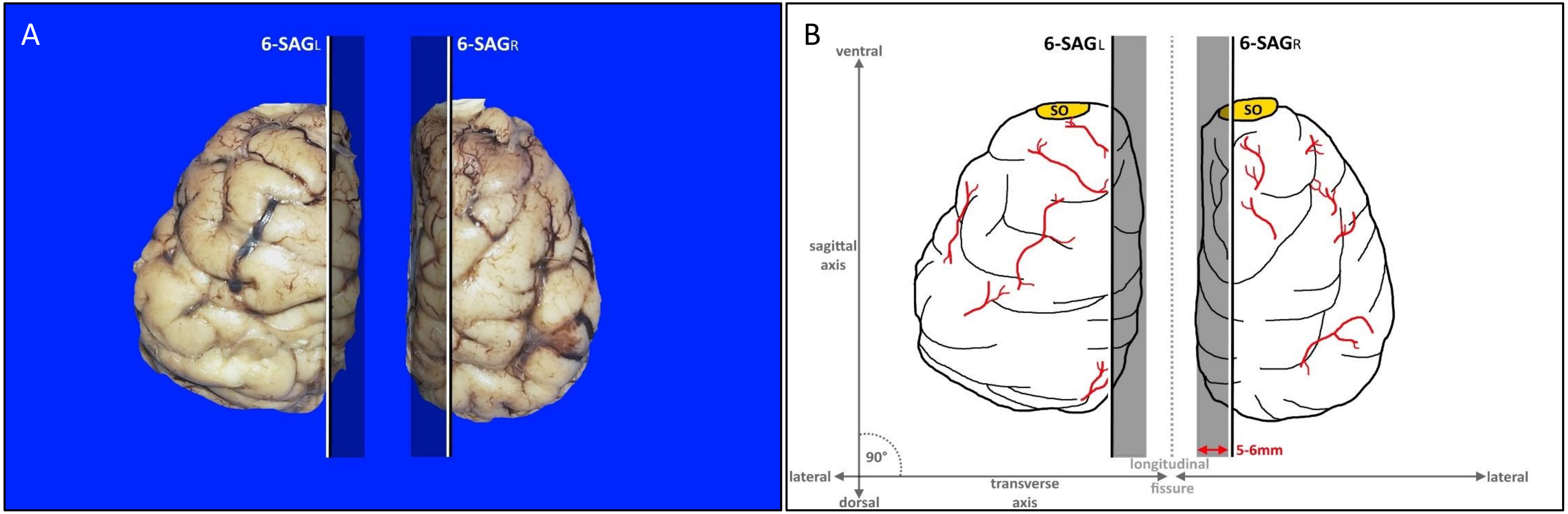

Supplementary Figure 11. Landmark for 6-SAG$_{L}$ and 6-SAG$_{R}$: stria olfactoria (SO). Sagittal section (SAG). Blood vessels (red), pertinent landmark (yellow), requested slab (graying). (A) Fixed brain. (B) Schematic illustration.

Supplementary Table 5 Instruction for obtainment of 4th slab out of Block B (Supplementary Figure 11)

**Step**	**Positioning and Preparation**	**View / specimen**	**Landmarks and cutting levels**	**Orientation of sections**	**Exposed structures**	**Difficulty**
**6-SAG$_{L}$** and **6-SAG$_{R}$**	Place the block with its caudal cutting surface on the worktop<br><br>The ventral aspect of the block is facing away from the investigator<br><br>Separate the hemispheres at longitudinal fissure	Rostro-caudal view	Stria olfactoria<br><br>Sagittal line through frontoparietal lobe 5-6mm lateral to midline	*2D knife axis:* dorsoventral<br><br>*Plane:* sagittal (SAG)<br><br>*Inclination:* orthogonal to worktop<br><br>*Blade movement:* rostrocaudal	• caudate nucleus<br>• lateral ventricle<br>• frontoparietal medial cortex	Easy

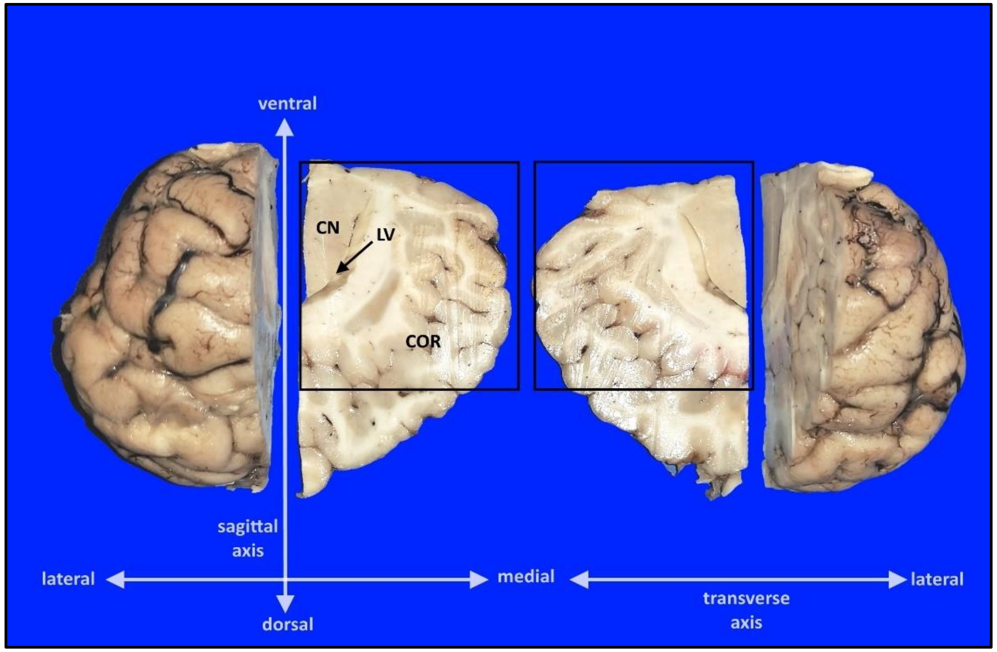

Supplementary Figure 11.1 Top view. 4th obtained slab of both hemispheres out of Block B. Axes (white) refer to brain blocks. Proposed area for histoprocessing (black frame). Pertinent landmarks for sampling: caudate nucleus (CN), frontoparietal cortex (COR), lateral ventricle (LV), white matter (WM).

Supplementary Table 5.1 Trimming of 4th slab according to sampling recommendations

Placement of the slab	View / specimen	Landmarks and cutting levels	Target structures	Slab size	Difficulty
Place the slab on its medial surface	Top view	Adjust to maximum slab size	subcortical white matter of gyrus marginalis/ cinguli (precruciate part)	maximum 4cm x 5.5cm (herein)	Easy

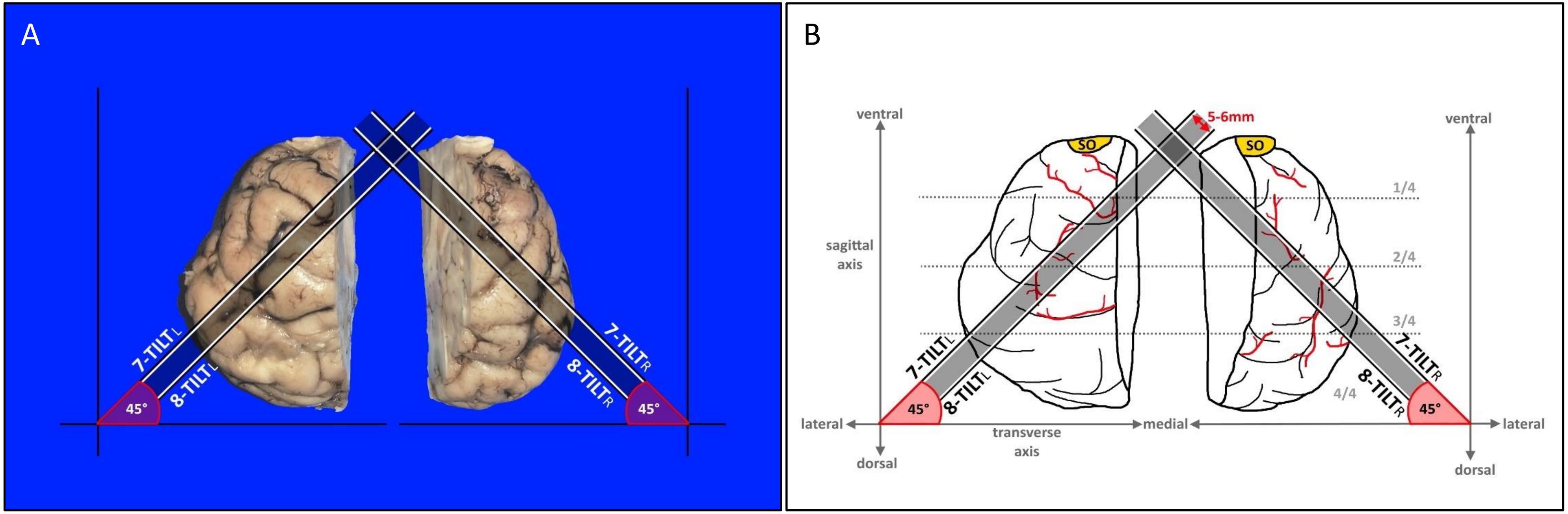

Supplementary Figure 12. Landmark for 7-TILT$_{L/R}$ and 8-TILT$_{L/R}$: stria olfactoria (SO). Tilted section (TILT). Blood vessels (red), pertinent landmark (yellow), requested slab (graying). (A) Fixed brain. (B) Schematic illustration.

Supplementary Table 6 Instructions for obtainment of 5th slab out of Block B (Supplementary Figure 12)

Step	Positioning and Preparation	View / specimen	Landmarks and cutting levels	Orientation of sections	Exposed structures	Difficulty
**7-TILT**$_L$ and **7-TILT**$_R$	Maintain placement as stated above	as above	Stria olfactoria<br><br>Tilted line through frontal lobe decussating an imaginary border to the most ventral ¼ of the block	*2D knife axis:* laterodorsal to medioventral<br><br>*Plane:* tilted (TILT) (45°) to dorsoventral axis<br><br>*Inclination:* orthogonal to worktop<br><br>*Blade movement:* rostrocaudal	• caudate nucleus<br>• lateral ventricles<br>• white matter tracts<br>• frontal (motor) cortex	Requires some practice

**8-TILT**$_L$ and **8-TILT**$_R$	Maintain placement as stated above	as above	Tilted line through frontal lobe parallel to and at 5-6 mm interslice distance dorsal to 7-TILT$_{L/R}$	as above	as above	Requires some practice

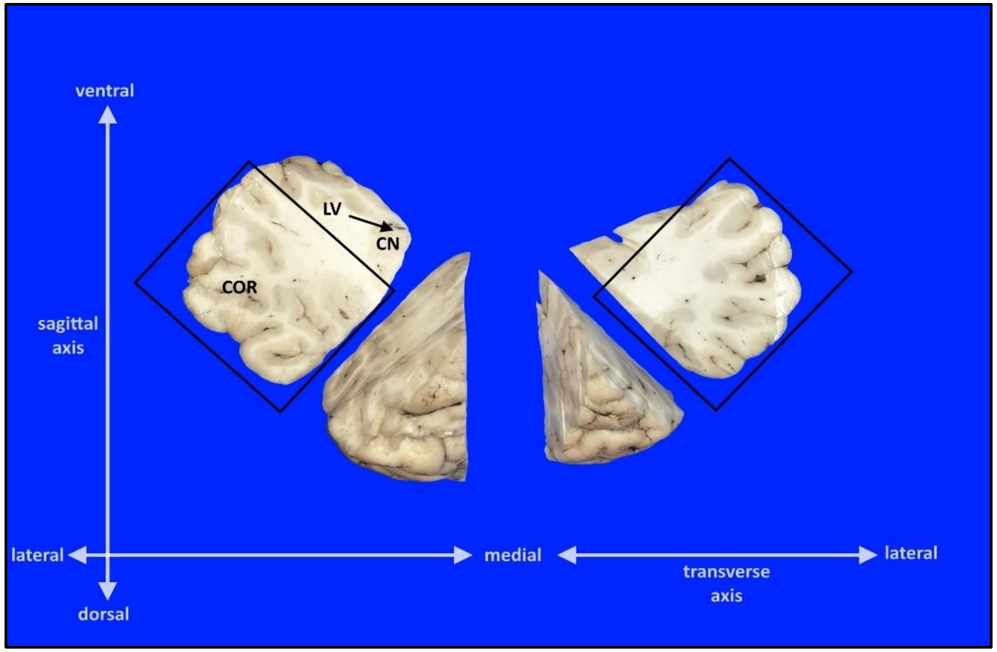

Supplementary Figure 12.1. Top view. 5th obtained slab of both hemispheres out of Block B. Axes (white) refer to brain blocks. Proposed area for histoprocessing (black frame). Pertinent landmarks for sampling: caudate nucleus (CN), cortex (COR), lateral ventricle (LV).

Supplementary Table 6.1 Trimming of 5th slab according to sampling recommendations

**Placement of the slab**	**View / specimen**	**Landmarks and cutting levels**	**Target structures**	**Slab size**	**Difficulty**
Ventrolateral cutting surface[7]	Top view	Adjust to maximum slab size	• rostral composite gyrus<br>• Betz cells (histological confirmation)	maximum 4cm x 5.5cm (herein)	Easy

[7] use the side/surface better exposing the target structures resp. the lesion

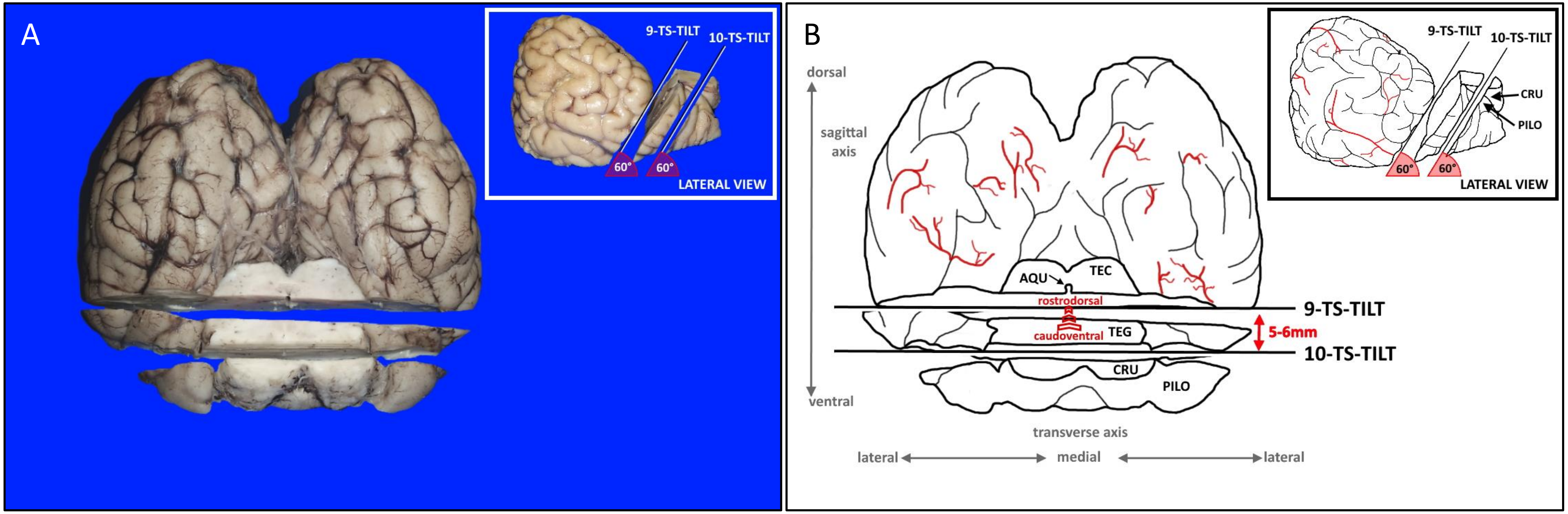

Supplementary Figure 13. Landmarks for 9-TS-TILT and 10-TS-TILT: mesencephalic aqueduct (AQU), cerebral crus (CRU), piriform lobe (PILO), midbrain tectum (TEC), midbrain tegmentum (TEG). Transverse-tilted section (TS-TILT). Blood vessels (red), pertinent landmark (yellow), requested slab (graying). (A) Fixed brain. (B) Schematic illustration.

Supplementary Table 7 Instructions for obtainment of 6th slab out of Block C (Supplementary Figure 13)

Step	Positioning and Preparation	View / specimen	Landmarks and cutting levels	Orientation of sections	Exposed structures	Difficulty
**9-TS-TILT**	Place the block with its rostral cutting surface on the worktop<br><br>The dorsal aspect of the block is facing away from the investigator	Caudo-rostral view	Transverse-tilted line through piriform lobe and midbrain tectum/ tegmentum<br><br>Cutting guide directly ventral of/ resp. through mesencephalic aqueduct	*2D knife axis:* laterolateral<br><br>*Plane:* tilted to PILO surface<br><br>*Inclination:* tilted (TILT) (60°) to worktop<br>➔ please note inlet for elucidation of approach<br><br>*Blade movement:* caudoventral to rostrodorsal	• brain stem nuclei<br>• lateral geniculate nucleus<br>• temporoventral body of the hippocampus<br>• cortex (temporal)	Requires some practice

**10-TS-TILT**	Maintain placement as stated above	as above	Transverse-tilted line through piriform lobe and midbrain tegmentum/ cerebral crus<br><br>Parallel to and at 5-6mm interslice distance ventral to 9-TS-TILT	as above	as above	Requires some practice

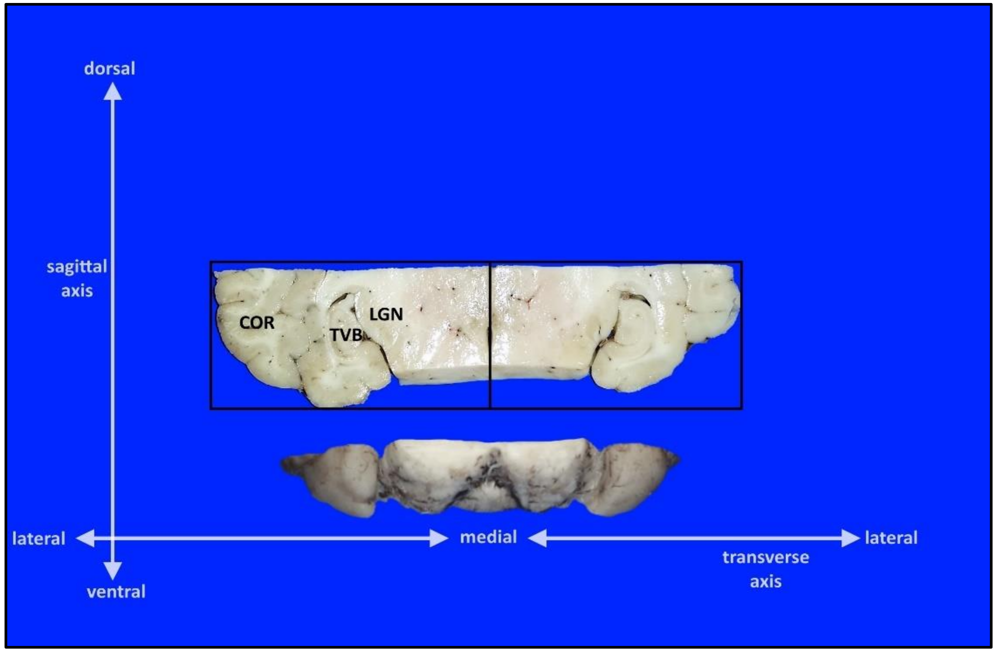

Supplementary Figure 13.1. Top view. 6th obtained slab out of Block C. Axes (white) refer to brain block. Proposed area for histoprocessing (black frame). Pertinent landmarks for sampling: cortex (COR), lateral geniculate nucleus (LGN), temporoventral body (TVB) of hippocampus.

Supplementary Table 7.1 Trimming of 6th slab according to sampling recommendations

**Placement of the slab**	**View / specimen**	**Landmarks and cutting levels**	**Target structures**	**Slab size**	**Difficulty**
Ventral cutting surface[8]	Top view	Vertical line at midline to adjust to maximum slab size	• cortex (temporal)<br>• temporoventral body of the hippocampus<br>• lateral geniculate nucleus<br>• midbrain nuclei	maximum 4cm x 5.5cm (herein)	Easy

[8] use the side/surface better exposing the target structures resp. the lesion

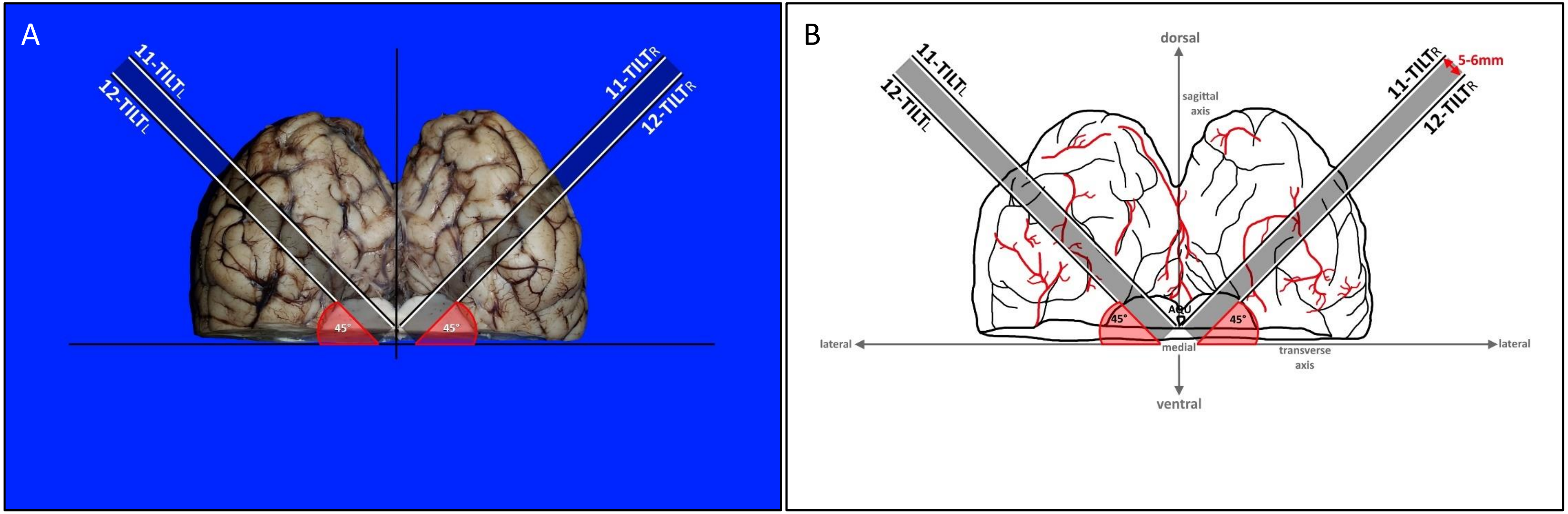

Supplementary Figure 14. Landmarks for 11-TILT$_{L/R}$ and 12-TILT$_{L/R}$. Mesencephalic aqueduct (AQU). Tilted section (TILT). Blood vessels (red), pertinent landmark (yellow), requested slab (graying). (A) Fixed brain. (B) Schematic illustration.

Supplementary Table 8 Instructions for obtainment of 7th slab out of Block C (Supplementary Figure 14)

Step	Positioning and Preparation	View / specimen	Landmarks and cutting levels	Orientation of sections	Exposed structures	Difficulty
**11-TILT$_L$** and **11-TILT$_R$**	Maintain placement as stated above	as above	Tilted line through occipital lobe and mesencephalic stump decussating the mesencephalic aqueduct	*2D knife axis:* medioventral to laterodorsal<br><br>*Plane:* tilted (TILT) (45°)<br><br>*Inclination:* orthogonal to worktop<br><br>*Blade movement:* caudorostral	• brain stem nuclei<br>• optic radiations<br>• main visual cortex<br>• occipital vertex of the hippocampus and associated parahippocampal gyrus	Requires some practice

**12-TILT**$_L$ and **12-TILT**$_R$	Maintain placement as stated above	as above	Tilted line through occipital lobe and mesencephalic stump at 5-6 mm interslice distance ventrolateral to 11-TILT$_{L/R}$	as above	as above	Requires some practice

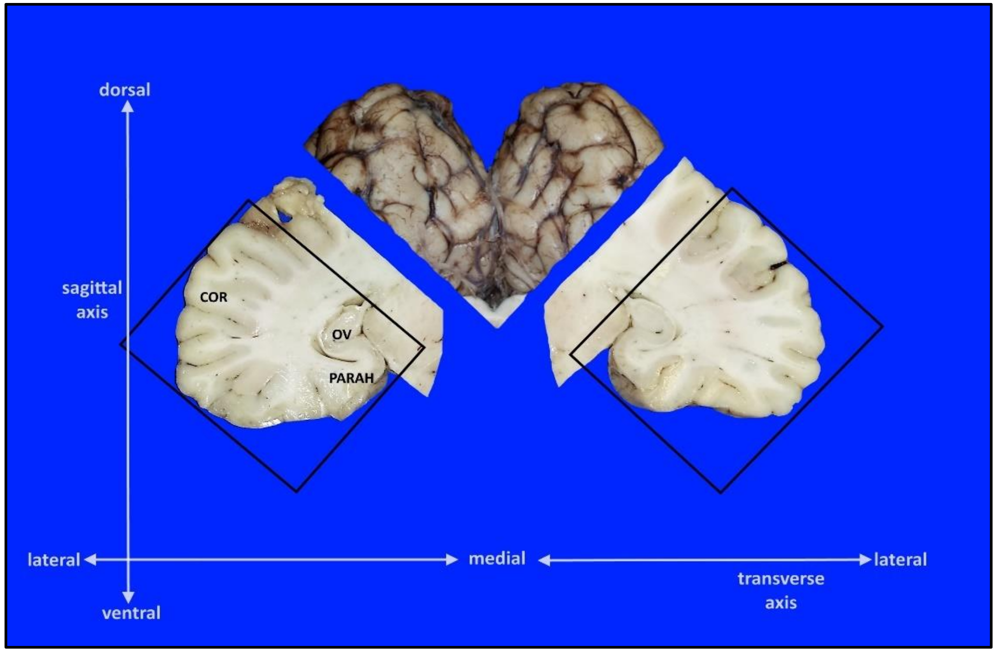

Supplementary Figure 14.1. Top view. 7th obtained slab of both hemispheres out of Block C. Axes (white) refer to brain block. Proposed area for histoprocessing (black frame). Pertinent landmarks for sampling: cortex (COR), occipital vertex (OV) of hippocampus, parahippocampal gyrus (PARAH).

SupplementaryTable 8.1 Trimming of 7th slab according to sampling recommendations

Placement of the slab	View / specimen	Landmarks and cutting levels	Target structures	Slab size	Difficulty
Ventrolateral cutting surface[9]	Top view	Adjust to maximum slab size	• optic radiations<br>• main visual cortex<br>• occipital vertex of the hippocampus<br>• occipital apex of parahippocampal gyrus	maximum 4cm x 5.5cm (herein)	Easy

[9] use the side/surface better exposing the target structures resp. the lesion

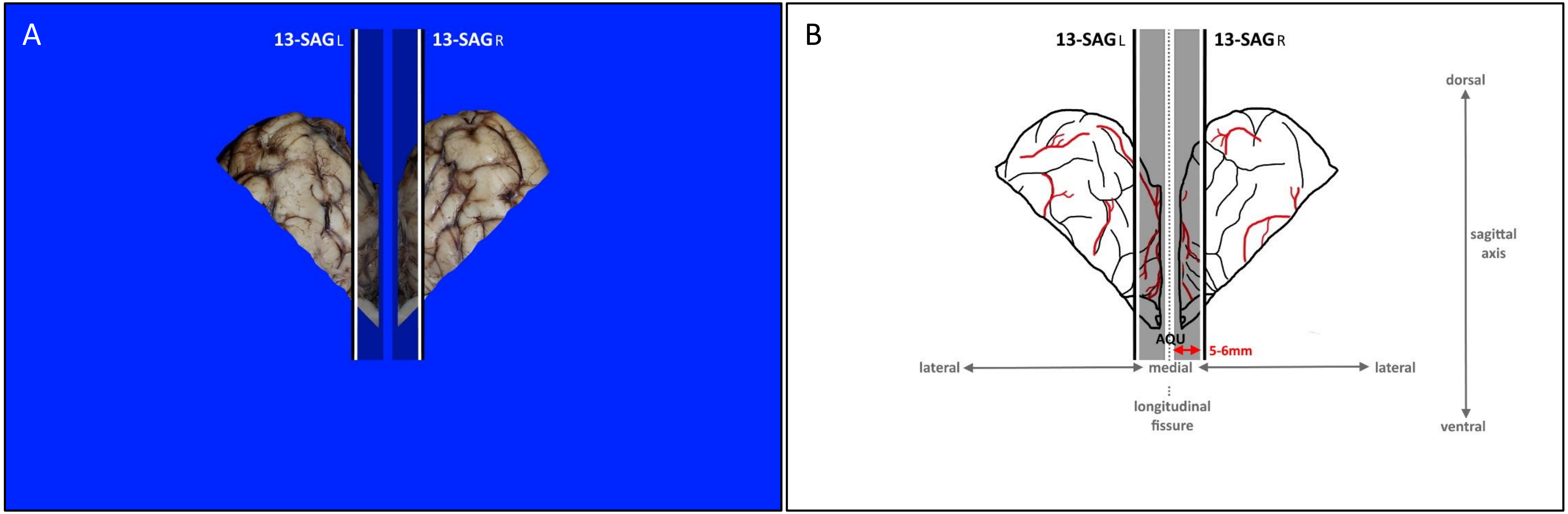

Supplementary Figure 15. Landmarks for 13-SAG$_{L/R}$: mesencephalic aqueduct (AQU). Sagittal section (SAG). Blood vessels (red), pertinent landmark (yellow), requested slab (graying). (A) Fixed brain. (B) Schematic illustration.

Supplementary Table 9 Instructions for obtainment of 8th slab out of Block C (Supplementary Figure 15)

Step	Positioning and Preparation	View / specimen	Landmarks and cutting levels	Orientation of sections	Exposed structures	Difficulty
**13-SAG$_L$** and **13-SAG$_R$**	Maintain placement as stated above<br><br>Separation of hemispheres at longitudinal fissure	as above	Sagittal line through occipital lobe and midbrain tectum 5-6mm lateral to midline after separation of hemispheres at longitudinal fissure	*2D knife axis:* ventrodorsal<br><br>*Plane:* sagittal (SAG)<br><br>*Inclination:* orthogonal to worktop<br><br>*Blade movement:* caudorostral	• subcortical white matter of gyrus marginalis/cingula (postcruciate part)<br>• hippocampal alveus<br>• longitudinal view of splenial sulcus	Easy

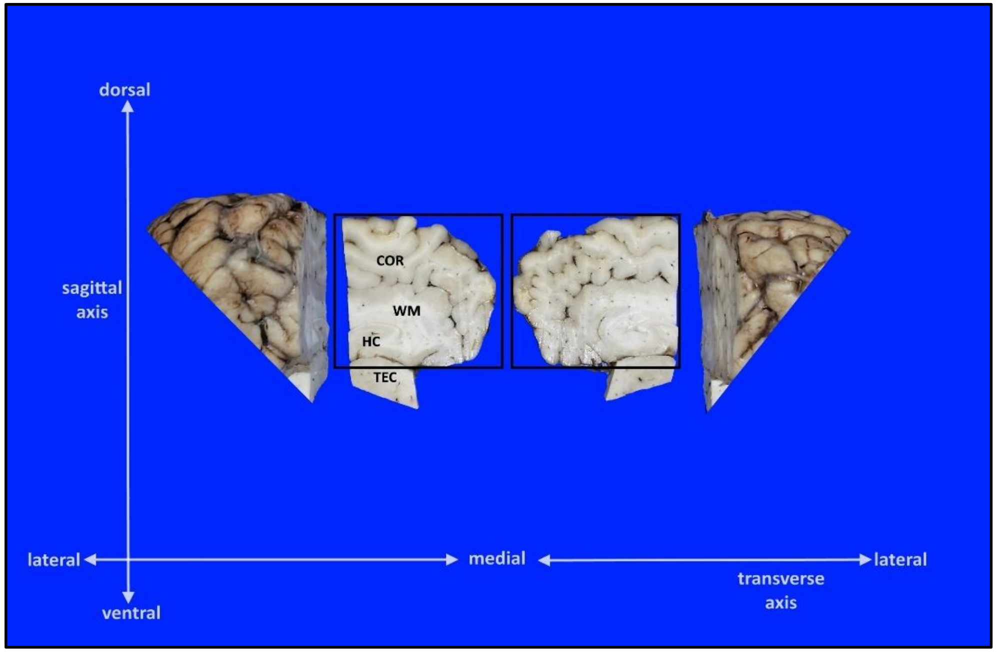

Supplementary Figure 15.1. Top view. 8th obtained slab of both hemispheres out of Block C. Axes (white) refer to brain blocks. Proposed area for histoprocessing (black frame). Pertinent landmarks for sampling: occipital cortex (COR), alveus of hippocampus (HC), midbrain tectum (TEC), white matter (WM).

Supplementary Table 9.1 Trimming of 8th slab according to sampling recommendations

Placement of the slab	View / specimen	Landmarks and cutting levels	Target structures	Slab size	Difficulty
Place the slab on its medial surface	Top view	Adjust to maximum slab size if necessary	• subcortical white matter of gyrus marginalis/cingula (postcruciate part)<br>• hippocampal alveus<br>• paramedial occipital cortex (splenial sulcus)	maximum 4cm x 5.5cm (herein)	Easy

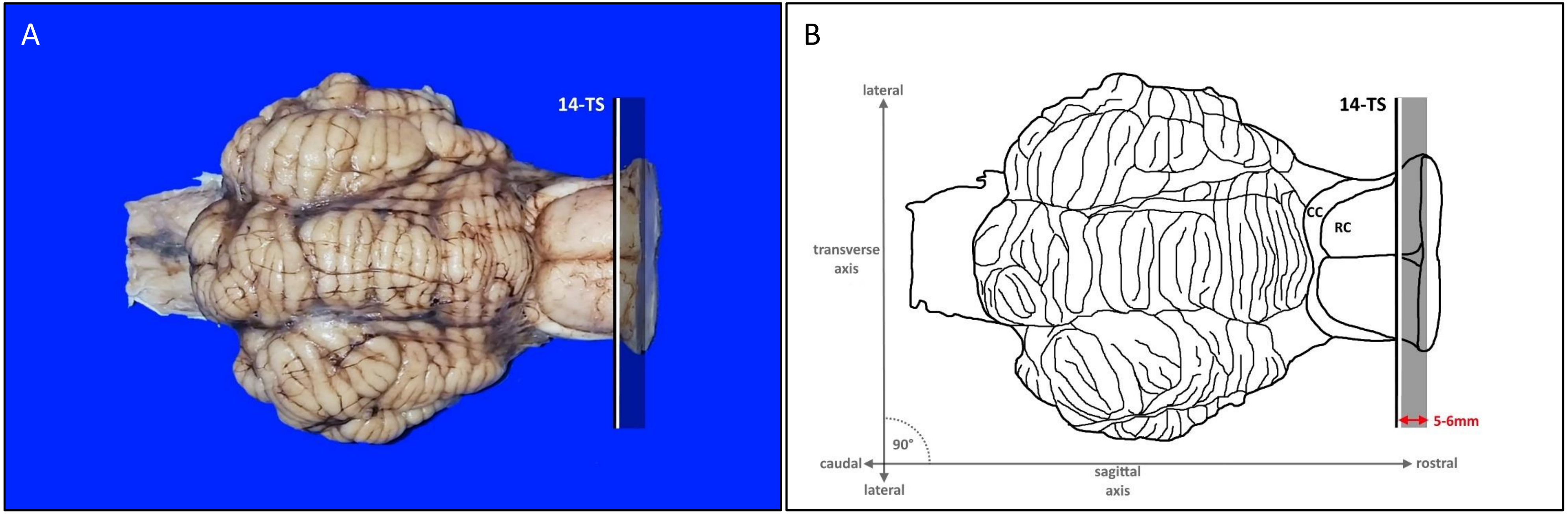

Supplementary Figure 16. Landmarks for 14-TS: caudal colliculi (CC), rostral coliculi (RC). Transverse section (TS). Requested slab (graying). (A) Fixed brain. (B) Schematic illustration.

Supplementary Table 10 Instruction for obtainment of 9th slab out of Block D (Supplementary Figure 16)

Step	Positioning and Preparation	View / specimen	Landmarks and cutting levels	Orientation of sections	Exposed structures	Difficulty
**14-TS**	Place the block with its ventral surface on the worktop	Dorsal view of the midbrain	Transverse line through rostral colliculi<br><br>resp. 5-6mm caudal to 1-TS[10]	*2D knife axis:* laterolateral<br><br>*Plane:* transverse (TS)<br><br>*Inclination:* orthogonal to worktop<br><br>*Blade movement:* dorsoventral	• geniculate nuclei<br>• midbrain tectum<br>• midbrain tegmentum<br>• aqueduct<br>• cerebral crus	Easy

[10] 1-TS = 1st cut (pre-step)

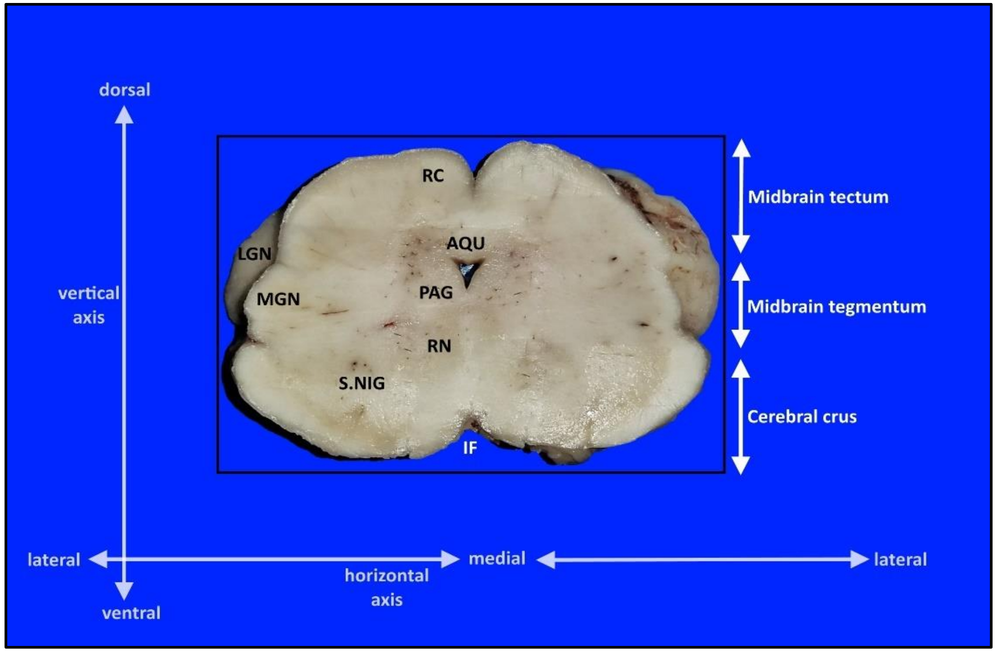

Supplementary Figure 16.1. Top view. 9th obtained slab out of Block D. Proposed area for histoprocessing (black frame). The slab is placed on its rostral cutting surface[11].Pertinent landmarks for sampling: mesenephalic aqueduct (AQU), intercrural fossa (IF); lateral geniculate nucleus (LGN), medial geniculate nucleus (MGN), periaqueductal gray matter (PAG), rostral colliculi (RC), red nucleus (RN), substantia nigra (S.NIG).

[11] use the side/surface better exposing the target structures resp. the lesion

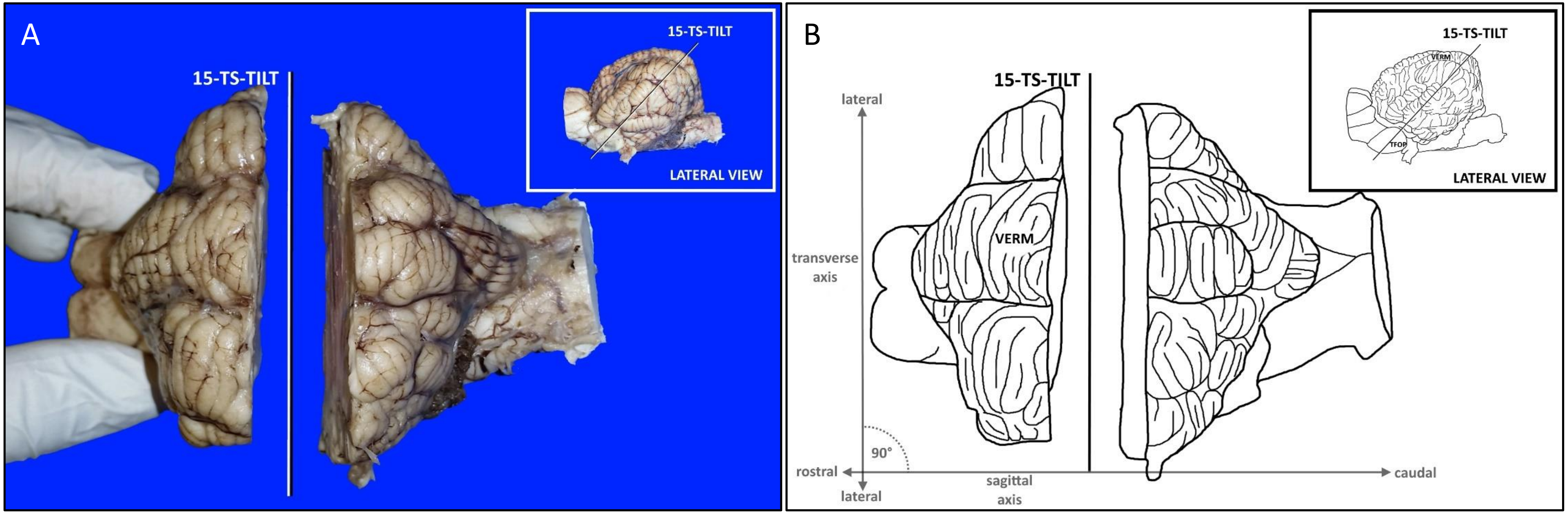

Supplementary Figure 17. Landmarks for 15-TS-TILT: transverse fibres of pons (TFOP), vermis (VERM). Transverse-tilted section (TS-TILT). (A) Fixed brain. (B) Schematic illustration.

Supplementary Table 11 Instruction for obtainment of 10th slab out of Block D (Supplementary Figure 17)

**Step**	**Positioning and Preparation**	**View / specimen**	**Landmarks and cutting levels**	**Orientation of sections**	**Exposed structures**	**Difficulty**
**15-TS-TILT**	Place the block with its ventral surface on the worktop	Dorsal view of the cerebellum	Transverse tilted line at widest lateral extension of the cerebellum through cerebellum and pons (TFOP)	*2D knife axis:* laterolateral<br><br>*Plane:* transverse (TS), tilted (TILT)<br><br>*Inclination:* Inclination required to meet the pons (TFOP)<br>➔ please note inlet for elucidation of approach<br><br>*Blade movement:* dorsocaudal to ventrorostral	• cerebellar cortex, nuclei and peduncles<br>• reticular formation<br>• 4th ventricle<br>• transverse fibres of pons	Requires some practice

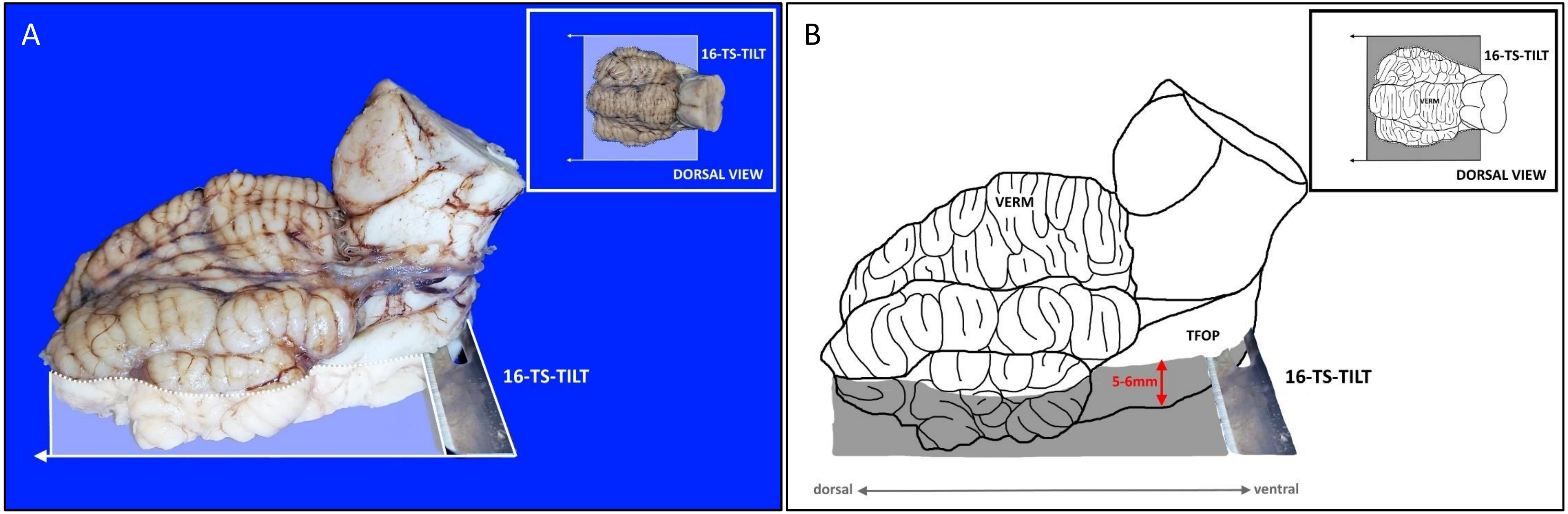

Supplementary Figure 18. Landmarks for 16-TS-TILT: transverse fibres of pons (TFOP), vermis (VERM). Transverse-tilted section (TS-TILT). Requested slab (graying). (A) Fixed brain. (B) Schematic illustration.

Supplementary Table 12 Instruction for obtainment of 10th slab out of Block D (Supplementary Figure 18)

Step	Positioning and Preparation	View / specimen	Landmarks and cutting levels	Orientation of sections	Exposed structures	Difficulty
**16-TS-TILT**	Place the rostral cerebellar block with its caudal cutting surface on the worktop	Lateral view of the rostral cerebellar block	Horizontal line 5-6mm rostral and parallel to 15-TS-TILT	*2D knife axis:* lateral laterolateral<br><br>*Plane:* transverse (TS), tilted (TILT)<br><br>*Inclination:* parallel to worktop<br>➔ please note inlet for elucidation of approach<br><br>*Blade movement:* ventrorostral to dorsocaudal	as 15-TS-TILT	Requires some practice

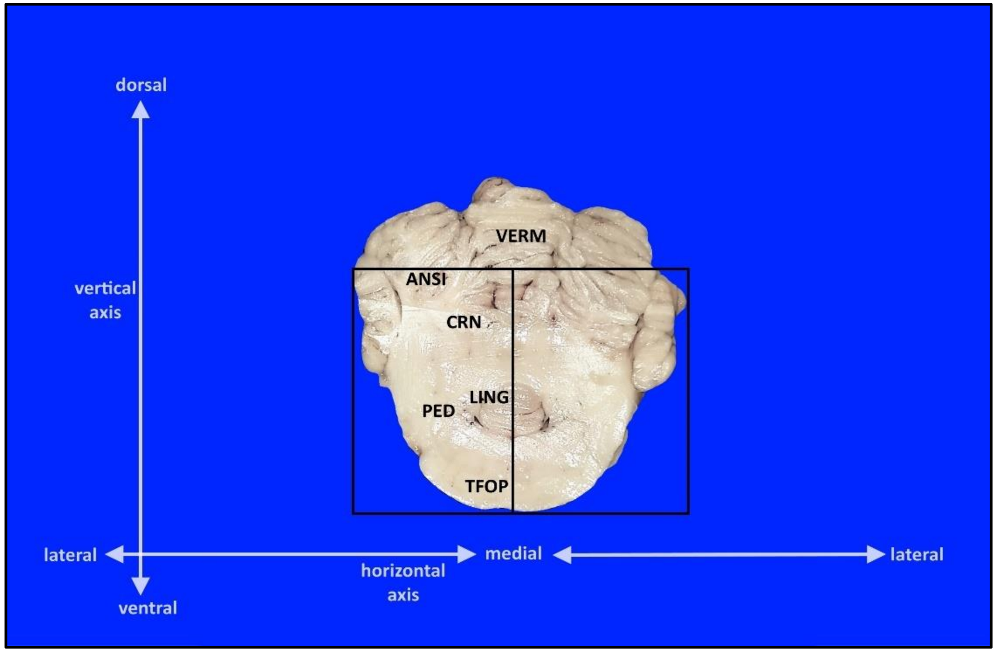

Supplementary Figure 18.1. Top view. 10th obtained slab out of block D. Proposed area for histoprocessing (black frame). Pertinent landmarks for sampling: ansiform lobule (ANSI), cerebellar roof nuclei (CRN), lingula (LING), cerebellar peduncles (PED), transverse fibres of ponst (TFOP), vermis (VERM).

Supplementary Table 12.1 Trimming of 10th slab according to sampling recommendations

Placement of the slab	View / specimen	Landmarks and cutting levels	Target structures	Slab size	Difficulty
Rostral cutting surface[12]	Top view	Adjust to maximum slab size	• cerebellar roof nuclei<br>• cerebellar peduncles<br>• transverse fibres of pons<br>• lingula	maximum 4cm x 5.5cm (herein)	Easy

[12] use the side/surface better exposing the target structures resp. the lesion

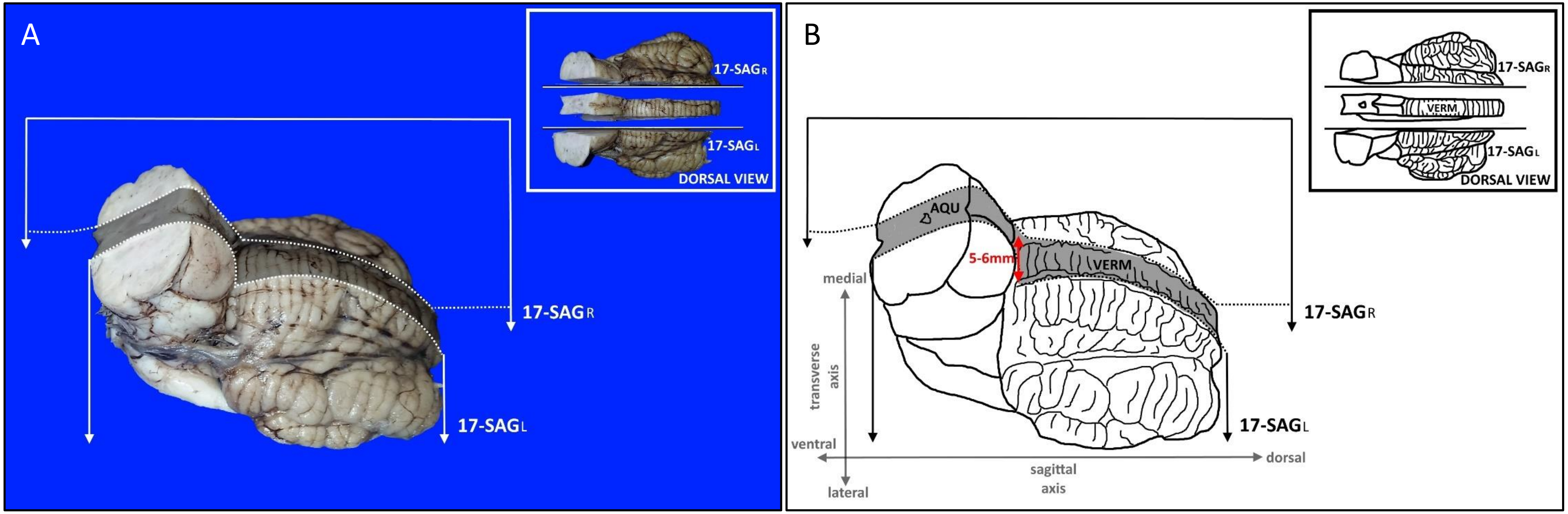

Supplementary Figure 19. Landmarks for 17-SAG$_{L/R}$: mesencephalic aqueduct (AQU), vermis (VERM). Sagittal section (SAG). Requested slab (graying). (A) Fixed brain. (B) Schematic illustration.

Supplementary Table 13 Instruction for obtainment of 11th slab out of Block D (Supplementary Figure 19)

Step	Positioning and Preparation	View / specimen	Landmarks and cutting levels	Orientation of sections	Exposed structures	Difficulty
**17-SAG$_L$** and **17-SAG$_R$**	Place the rostral cerebellar block with its caudal cutting surface on the worktop	Dorsal view of the rostral cerebellar block	Sagittal line approx. 3mm lateral to midline	*2D knife axis:* dorsoventral<br><br>*Plane:* sagittal (SAG)<br><br>*Inclination:* orthogonal to worktop<br><br>*Blade movement:* rostrocaudal	• sagittal paravermis<br>• sagittal midbrain	Easy

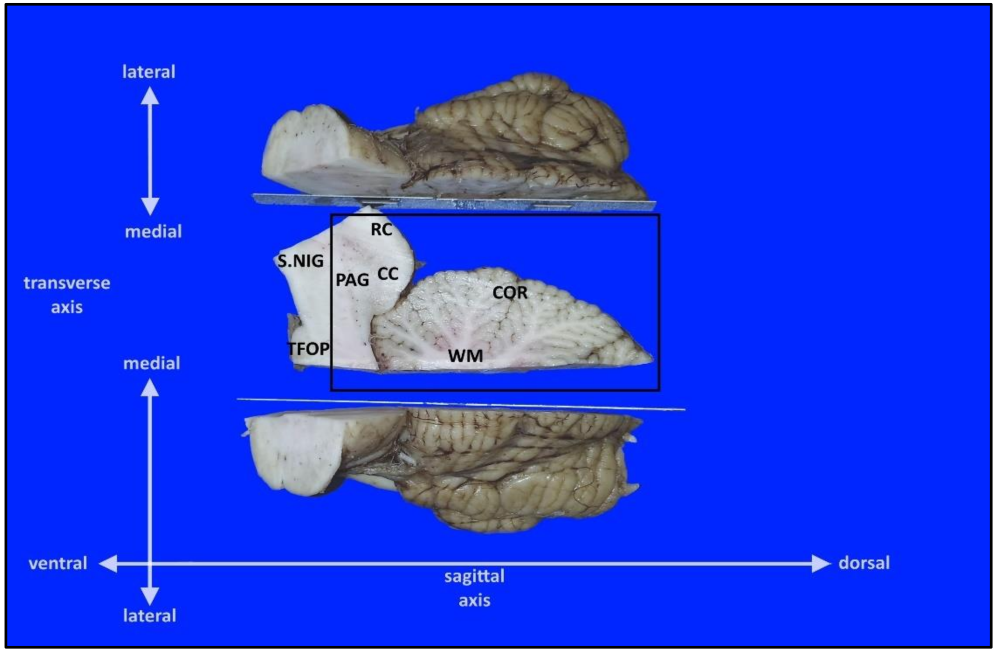

Supplementary Figure 19.1. Top view. 11th obtained slab out of Block D. Axes (white) refer to brain blocks. Proposed area for histoprocessing (black frame). Pertinent landmarks for sampling: caudal colliculi (CC), cerebellar cortex (COR), periaqueductal gray matter (PAG), rostral colliculi (RC), substantia nigra (S.NIG), transverse fibres of pons (TFOP), white matter (WM).

Supplementary Table 13.1 Trimming of 11th slab according to sampling recommendations

Placement of the slab	View / specimen	Landmarks and cutting levels	Target structures	Slab size	Difficulty
Place the slab on its medial or lateral cutting surface[13]	Top view	Adjust to maximum slab size	sagittal paravermis	maximum 4cm x 5.5cm (herein)	Easy

[13] use the side/surface better exposing the target structures resp. the lesion

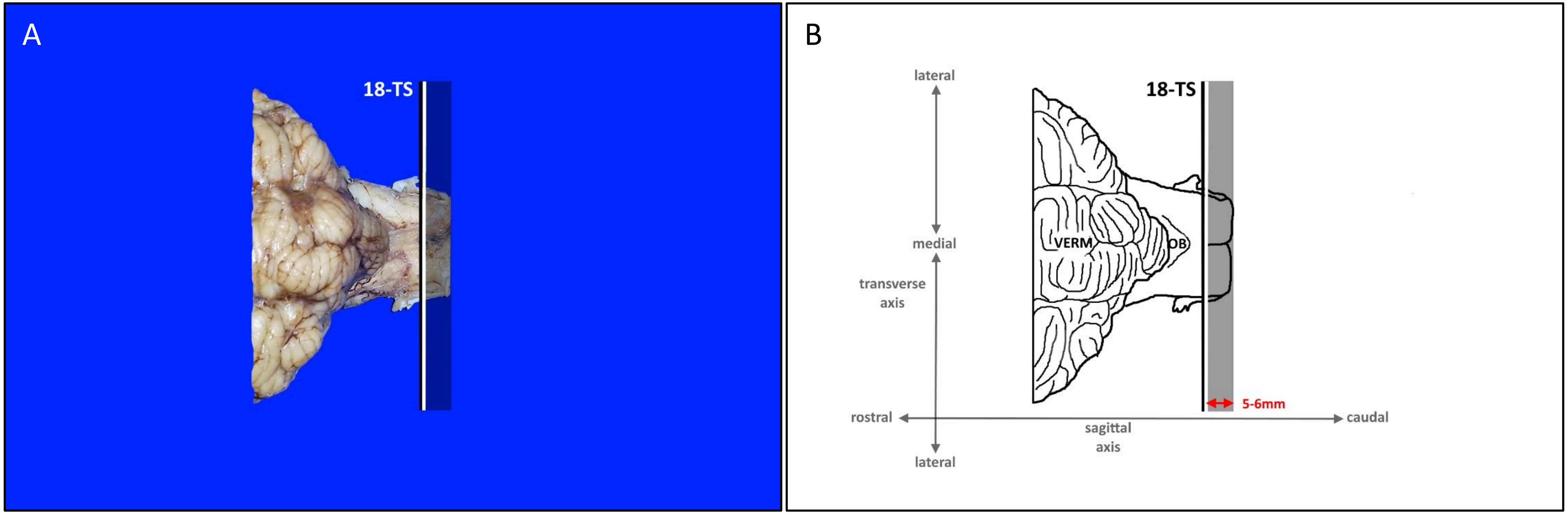

Supplementary Figure 20. Landmarks for 18-TS: obex (OB: obex), vermis (VERM). Transverse section (TS). Requested slab (graying). (A) Fixed brain. (B) Schematic illustration.

Supplementary Table 14 Instruction for obtainment of 12th slab out of Block D (Supplementary Figure 20)

**Step**	**Positioning and Preparation**	**View / specimen**	**Landmarks and cutting levels**	**Orientation of sections**	**Exposed structures**	**Difficulty**
**18-TS**	Place the caudal cerebellar block with its ventral surface on the worktop	Dorsal view of the medulla oblongata	Transverse line through medulla oblongata caudal to the obex	*2D knife axis:* laterolateral<br><br>*Plane:* transverse (TS)<br><br>*Inclination:* orthogonal to worktop<br><br>*Blade movement:* dorsoventral	• Medulla oblongata	Easy

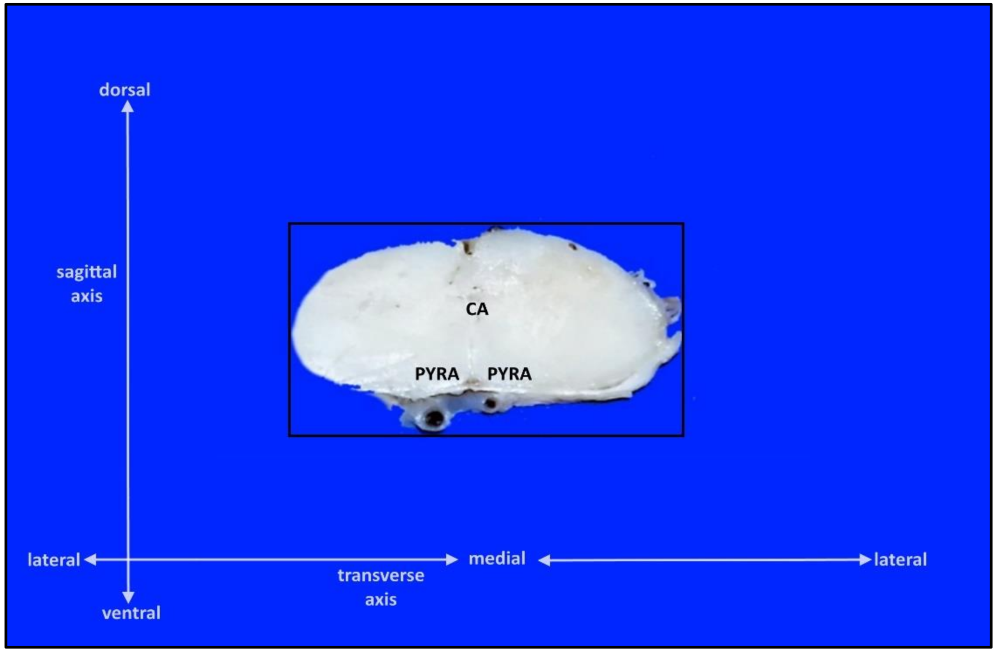

Supplementary Figure 20.1: Top view. The slab is placed on its rostral cutting surface[14]. 12th obtained slab out of Block D. Proposed area for histoprocessing (black frame). Pertinent landmarks for sampling: central canal (CA), pyramids (PYRA).

[14] use the side/surface better exposing the target structures resp. the lesion

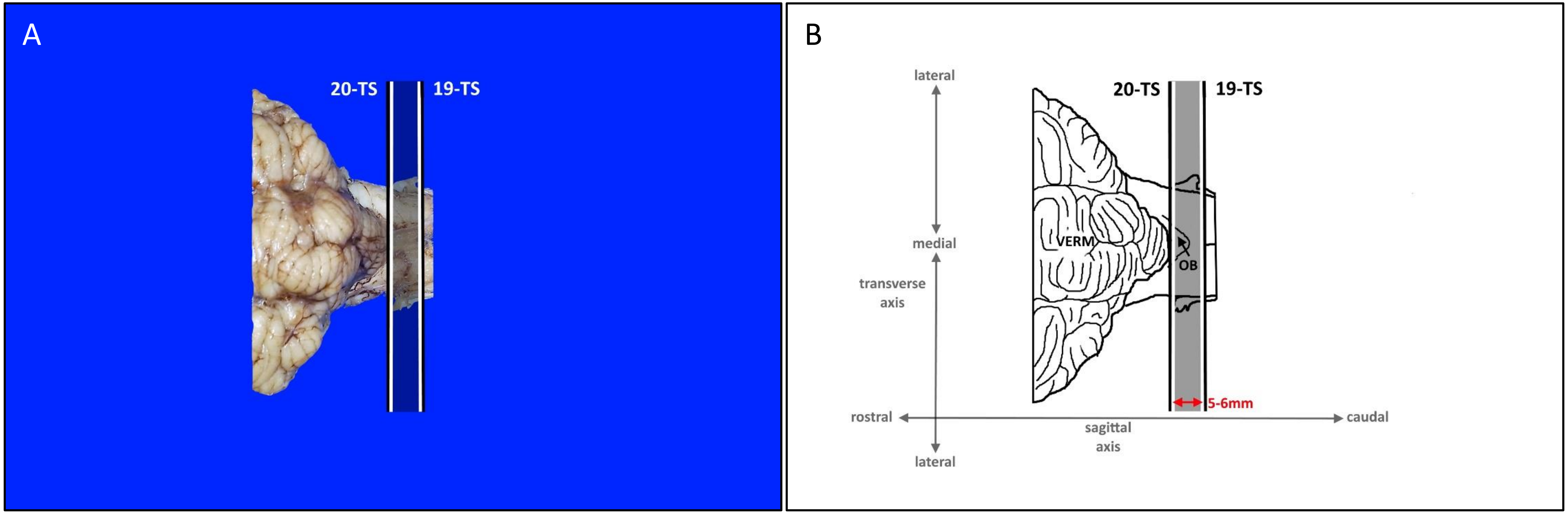

Supplementary Figure 21. Landmarks for 19-TS and 20-TS: obex (OB), vermis (VERM). Transverse section (TS). Requested slab (graying). (A) Fixed brain. (B) Schematic illustration.

Supplementary Table 15 Instruction for obtainment of 13th slab out of Block D (Supplementary Figure 21)

Step	Positioning and Preparation	View / specimen	Landmarks and cutting levels	Orientation of sections	Exposed structures	Difficulty
**19-TS**	Maintain placement as stated above	Dorsal view of the hindbrain	Transverse line near the obex	*2D knife axis:* laterolateral<br><br>*Plane:* transverse (TS)<br><br>*Inclination:* orthogonal to worktop<br><br>*Blade movement:* dorsoventral	• area postrema<br>• spinal tracts<br>• decussation of sensory fibres<br>• vagal and associated nuclei<br>• proprioceptive nuclei	Easy
**20-TS**	Maintain placement as stated above	as above	Transverse line 5-6 mm rostral and parallel to 19-TS	as above	as above	Easy

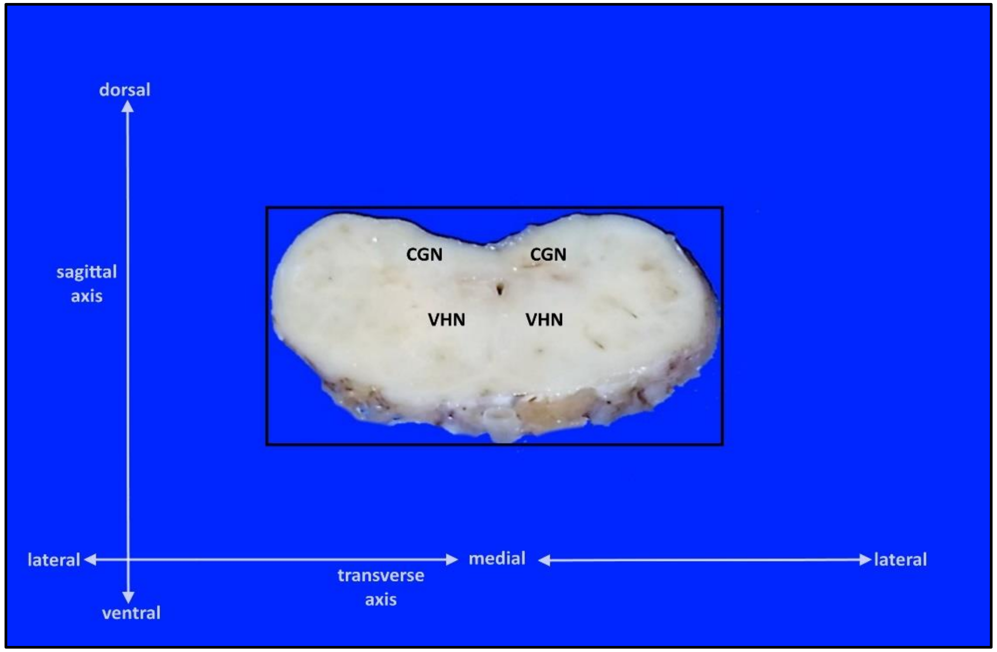

Supplementary Figure 21.1. Top view. The slab is placed on its rostral cutting surface[15]. 13th obtained slab out of Block D. Proposed area for histoprocessing (black frame). Pertinent landmarks for sampling: cuneate and gracile nuclei (CGN), vagal and hypoglossal nuclei (VHN).

[15] use the side/surface better exposing the target structures resp. the lesion

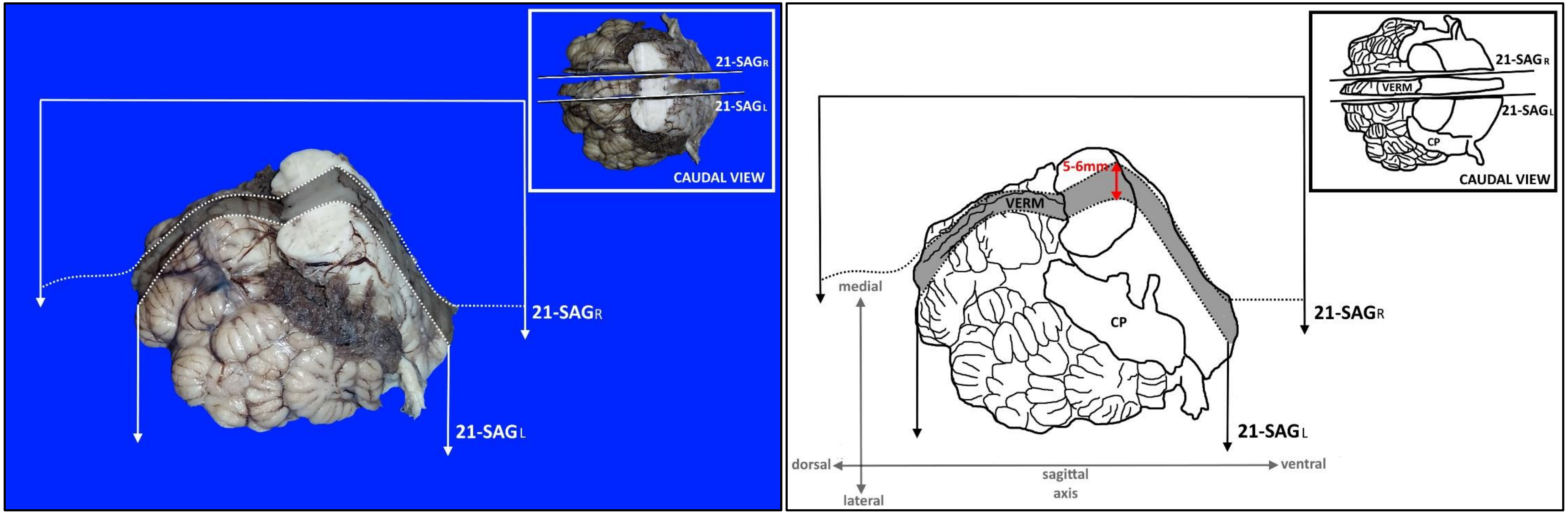

Supplementary Figure 22. Landmarks for 21-SAG$_{L/R}$: central canal (CA), choroid plexus (CP), vermis (VERM). Sagittal section (SAG). Requested slab (graying). (A) Fixed brain. (B) Schematic illustration.

Supplementary Table 16 Instruction for obtainment of 14th slab out of Block D (Supplementary Figure 22)

Step	Positioning and Preparation	View / specimen	Landmarks and cutting levels	Orientation of sections	Exposed structures	Difficulty
**21-SAG$_L$** and **21-SAG$_R$**	Place the caudal cerebellar block with its rostral cutting surface on the worktop	Caudal view of caudal cerebellar block	Sagittal line approx. 3mm lateral to midline	*2D knife axis:* ventrodorsal<br><br>*Plane:* sagittal (SAG)<br><br>*Inclination:* orthogonal to worktop<br><br>*Blade movement:* caudorostral	• sagittal mid-uvula<br>• brainstem nuclei<br>• medial wall of area postrema	Easy

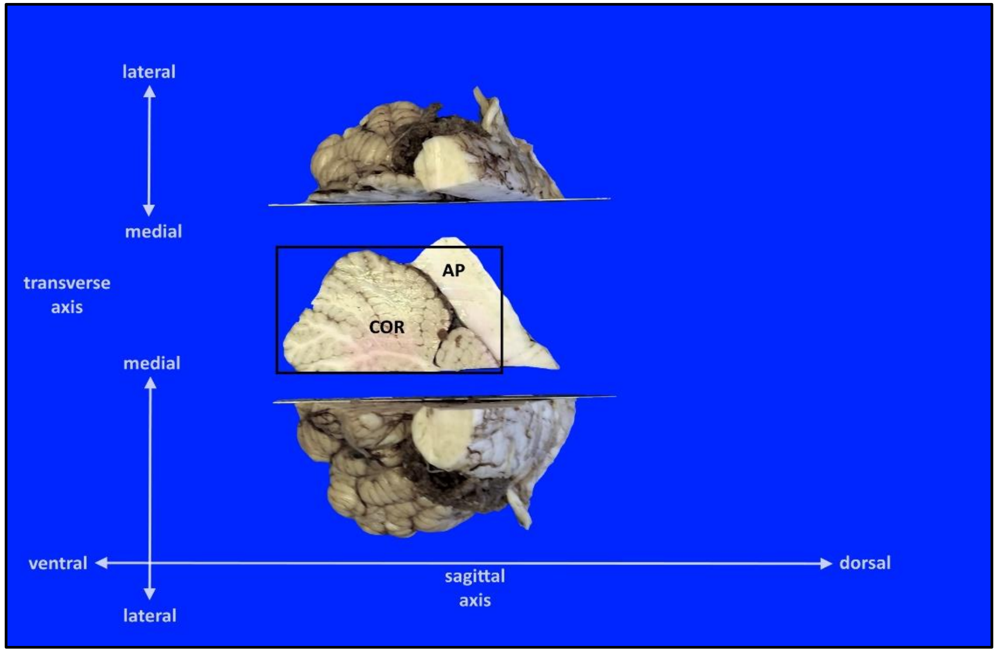

Supplementary Figure 22.1. Top view. 14th obtained slab out of Block D. Axes (white) refer to brain blocks. Proposed area for histoprocessing (black frame). Pertinent landmarks for sampling: area postrema (AP), cortex (COR).

Supplementary Table 16.1 Trimming of 14th slab according to sampling recommendations

Placement of the slab	View / specimen	Landmarks and cutting levels	Target structures	Slab size	Difficulty
Place the slab on its medial or lateral cutting surface[16]	Top view	Adjust to maximum slab size	• sagittal mid-uvula<br>• brainstem nuclei<br>• medial wall of area postrema	maximum 4cm x 5.5cm (herein)	Easy

[16] use the side/surface better exposing the target structures resp. the lesion

Abbreviations

I. Planes/ Orientation

L: left
R: right
SAG: sagittal plane/section
TILT: lateral-tilted plane/section
TS: transverse plane/section
TS-TILT: transverse-tilted plane/section

II. Landmarks

III VENT: 3rd ventricle
AN: amygdaloid nucleus
ANSI: ansiform lobule
AP: area postrema
AQU: mesencephalic aqueduct
CA: central canal
CC: caudal colliculi
CER: cerebellum
CGN: cuneate and gracile nuclei
CING: cingulate gyrus
CN: caudate nucleus
COCA: corpus callosum
COR: cortex
CP: choroid plexus
CRN: cerebellar roof nuclei
CRU: cerebral crus
FORN: fornix
GP: globus pallidus
HC: hippocampus
HICO: hippocampal commissure
IC: internal capsule
INSC: insular cortex
LING: lingula
LGN: lateral geniculate nucleus
LOF: longitudinal fissure
LV: lateral ventricle
MAM: mammillary bodies
MGN: medial geniculate nucleus
MOB: medulla oblongata
OB: obex
OC: optic chiasm
ON: oculomotor nerve
OLF: olfactory tubercle
OT: optic tract
OV: occipital vertex of hippocampus
PAG: periaqueductal gray matter
PARAH: parahippocampal gyrus
PED: cerebellar peduncles
PILO: piriform lobe

PIT: pituitary stalk/ infundibular recess
PUT: putamen
PVZ: periventricular zone of the hypothalamus
PYRA: pyramids (decussation)
RC: rostral colliculi
RN: red nucleus
S.NIG: substantia nigra
SN: septal nuclei
SO: stria olfactoria
TEC: midbrain tectum
TEG: midbrain tegmentum
TFOP: transverse fibres of pons
THAL: thalamus
TVB: temporoventral body of the hippocampus
VAN: ventral anterior nucleus
VERM: vermis
VHN: vagal and hypoglossal nuclei
WM: white matter
